# Supplementary material for: Exploring polygenic‐environment and residual‐environment interactions for depressive symptoms within the UK Biobank
Source: Genet Epidemiol. 2022 Apr 19;46(5-6):219–33. doi: 10.1002/gepi.22449 (PMC9541465; doi:10.1002/gepi.22449)
Supplement: Supplementary file 1 — Supporting information. [file GEPI-46-219-s001.pdf]

Supplementary Materials

Alexandra C. Gillett Bradley S. Jermy S. Hong Lee Oliver Pain
David M. Howard Saskia P. Hagenaars Ken B. Hanscombe
Jonathan R. I. Coleman Cathryn M. Lewis

Contents

1 Supplementary Methods **4**
1.1 depSympt: understanding the outcome trait 4
1.2 Phenotype adjustment 13
1.3 Multivariate reaction norm model 16
1.3.1 Model introduction 16
1.3.2 Model definition 17
1.4 Meta-analysis methods 24
1.4.1 Likelihood ratio test 24
1.4.2 Variance-covariance model parameters: estimates and standard errors 24
1.4.3 Genetic, residual and total variance components for standardised residual depSympt as a function of the covariate trait: estimate and SEs 25
1.4.4 Re-scaling the proportion of variability in depSympt attributable to C_j: estimates and standard errors 27

2 Supplementary Tables **31**
2.1 Covariate traits 31
2.2 Fractional polynomial interaction models 36
2.3 Notes on Tables 15 and 16 43

3 Supplementary Figures **45**
3.1 Covariate traits distributions 45
3.1.1 Covariates in Analysis group 1 45
3.1.2 Covariates in Analysis group 2 56
3.1.3 Covariates in Analysis group 3 60
3.1.4 Covariates in Analysis group 4 61
3.1.5 Covariates in Analysis group 5 62
3.1.6 Biomarker distribution plots: untransformed compared to log transformed 63

3.2 Fractional polynomial model results 66

List of Tables

1 The 18 original symptom variables selected from UKB to be used in the factor analysis which created depSympt. 5
4 The relationship between depSympt and the current depressive symptoms. 7
2 Proportion of variation in liability to depression explained by 6 latent depression symptom scores, including dedpSympt, within current UKB study sample 10
3 Correlation matrix for the six latent depression symptom scores. 10
5 Additional variables used in fixed effects adjustment of depSympt. 15
6 Characteristics of depSympt and the covariate traits in available UK Biobank sample. 31
7 Linear fixed effects model summary. 32
8 Comparison of the proportion of variability in depSympt explained by each covariate trait: main effects only versus fractional polynomials 33
9 Fractional polynomial fixed effects model summary. 34
10 Likelihood ratio test statistics and p-values comparing the full versus the null multivariate reaction norm models. 36
11 LRT statistics and p-values: all covariates, all subgroups 37
12 Likelihood ratio test statistics and p-values when comparing models with and without interactions, using untransformed depSympt and RINT depSympt (sensitivity analysis). 38
13 Percentage of variability in depSympt attributable to each covariate trait. 39
14 Percentage of variability in *residual* depSympt attributable to genotype-covariate (G-C) and residual-covariate (R-C) interactions. 40
15 Percentage of variability in residual depSympt[a] attributable to polygenic and residual variation[b]. 41
16 Percentage of variability in depSympt[a] attributable to the fixed effects[b] and to polygenic and residual variation[c]. 42

List of Figures

1 Visualisation for the model of depression symptom scores from Jermy et al. [2020] 11
2 depSympt density plot by major depressive disorder (MDD) status with descriptive statistics. 12
3 Histograms of standardised BMI 46
4 Histograms of the standardised childhood trauma summary variable . . . 47
5 Histograms of standardised MET total 48
6 Histograms of standardised MET walk 49

7 Histograms of standardised MET moderate 50
8 Histograms of standardised MET vigorous 51
9 Histograms of standardised average sleep duration 52
10 Histograms of standardised TDI 53
11 Histograms of standardised waist circumference 54
12 Histograms of standardised waist-to-hip ratio 55
13 Histograms of standardised log-CRP 56
14 Histograms of standardised log-Triglycerides 57
15 Histograms of standardised log-Vitamin D 58
16 Histograms of standardised LDL 59
17 Histograms of standardised log-HDL 60
18 Histograms of standardised Neuroticism score 61
19 Histograms of standardised pack-years smoking as a proportion of age . . 62
20 Histograms of standardised CRP and log-CRP 63
21 Histograms of standardised Triglycerides and log-Triglycerides 63
22 Histograms of standardised Vitamin D and log-Vitamin D 64
23 Histograms of standardised LDL and log-LDL 64
24 Histograms of standardised HDL and log-HDL 65
25 Forest-plot of the proportion of variation in depSympt attributable to: (a) a genotype-covariate interaction, and (b) a residual-covariate interaction, with 95% confidence intervals, for all 3 subgroups & the meta-analysis. . . 67
26 Forest-plot of the proportion of variation in depSympt attributable to: (a) a genotype-covariate interaction, and (b) a residual-covariate interaction, with 95% confidence intervals, for all 3 subgroups & the meta-analysis. All covariate traits. 68
27 Variance components for Y (residualised depSympt) against residualised and standardised neuroticism score. 69
28 Variance components for Y (residualised depSympt) against residualised and standardised childhood trauma summary variable. 70
29 Variance components for Y (residualised depSympt) against residualised and standardised average sleep duration. 71
30 Variance components for Y (residualised depSympt) against residualised and standardised BMI. 72
31 Variance components for Y (residualised depSympt) against residualised and standardised waist circumference. 73
32 Variance components for Y (residualised depSympt) against residualised and standardised smoking. 74
33 Variance components for Y (residualised depSympt) against residualised and standardised waist to hip ratio. 75
34 Variance components for Y (residualised depSympt) against residualised and standardised MET total. 76
35 Variance components for Y (residualised depSympt) against residualised and standardised MET walk. 77

36 Variance components for Y (residualised depSympt) against residualised and standardised MET moderate. 78
37 Variance components for Y (residualised depSympt) against residualised and standardised TDI. 79
38 Proportion of the total variance component for Y (residualised depSympt) against residualised and standardised average sleep duration. 80
39 Plots of the variance components for normalised depSympt across normalised MET (total) 81
40 Plots of the variance components for normalised depSympt across normalised MET (moderate) 82

1 Supplementary Methods

1.1 depSympt: understanding the outcome trait

depSympt was created by Jermy et al. [2020]. Using depression-related symptom data from the Mental Health Questionnaire (MHQ) within the UK Biobank (UKB), the authors performed a factor analysis to identify latent continuous factors that could be driving the observed symptoms. A hierarchical model with five first-order factors and one second-order factor was identified. Please see Table (1) for the inputted symptom data, and Figure (1) for a visualisation of the identified hierarchical model.

The first-order factors can be labelled by the group of symptoms that they capture: 1. Mood (capturing depressed thoughts, anhedonia and suicidal thoughts), 2. Anxiety (capturing symptoms related to anxiety, nervousness, worry, foreboding and restlessness), 3. Subjective well-being (related to general wellbeing, belief in meaningfulness of own life and suicidal thoughts), 4. Psychomotor Cognitive factor (capturing symptoms for impaired concentration, restlessness and psychomotor retardation or agitation), and, 5. Neuro-vegetative factor (capturing changes in appetite, energy and sleep).

The second order factor, which we call the depSympt, can be thought of as a continuous depression score involved in driving all of the identified first-order factors. As such, it is highly correlated with the five first-order factors, with correlations ranging between 0.73 and 0.96 within the MHQ sample used by Jermy et al. [2020] ($n = 148,957$), and ranging between 0.84 and ≈ 1 (see Tables (2) and (3)) within the reduced sample with genetic data available used in this work ($n = 119,690$).

Due to these high correlations, we selected the depSympt to be the outcome trait when investigating genotype-covariate (G-C) and residual-covariate (R-C) interactions for a depressive symptom trait. A low depSympt score is associated with having low severity or no depression-related symptoms at the time of taking the MHQ. Conversely, a high depSympt score is associated with having an increased number of symptoms, with an increased severity level. For details please see Table (4), which provides mean depSympt

across severity levels for each of the 15 symptoms included in the final factor analysis model.

Table (3) shows that 11.06% of the variability in liability to (prevalent) depression is attributable to depSympt (this is the highest of all of the created latent factors). Figure (2) presents a density plot for depSympt within the available UKB study population, grouped by MDD status, demonstrating that the average depSympt value for MDD cases is larger than that for controls (0.35 compared to -0.18). Therefore, although depSympt is a continuous summary variable capturing current depressive symptoms, it is also associated with being a prevalent MDD case. Interactions identified within this study would warrant investigation using case-control depression phenotypes.

Table 1: The 18 original symptom variables selected from UKB to be used in the factor analysis which created depSympt. Table taken from the Supplementary Materials from Jermy et al. [2020].

**Field**	**Symptom Class**	**Symptom**	**Question**
20510	Depressive Symptoms	Depressed mood	Over the last 2 weeks, how often have you been bothered by any of the following problems? Feeling down, depressed, or hopeless
20514	Depressive Symptoms	Anhedonia	Over the last 2 weeks, how often have you been bothered by any of the following problems? Little interest or pleasure in doing things
20511	Depressive Symptoms	Appetite loss or gain	Over the last 2 weeks, how often have you been bothered by any of the following problems? Poor appetite or overeating
20517	Depressive Symptoms	Insomnia or hypersomnia	Over the last 2 weeks, how often have you been bothered by any of the following problems? Trouble falling or staying asleep, or sleeping too much
20518	Depressive Symptoms	Psychomotor agitation or retardation	Over the last 2 weeks, how often have you been bothered by any of the following problems? Moving or speaking so slowly that other people could have noticed? Or the opposite- being so fidgety or restless that you have been

Continued on next page

Table 1 – continued from previous page

Field	Symptom Class	Symptom	Question
			moving around a lot more than usual
20519	Depressive Symptoms	Fatigue or loss of energy	Over the last 2 weeks, how often have you been bothered by any of the following problems? Feeling tired or having little energy
20507*	Depressive Symptoms	Feelings of inadequacy	Over the last 2 weeks, how often have you been bothered by any of the following problems? Feeling bad about yourself or that you are a failure or have let yourself or your family down
20508	Depressive Symptoms	Impaired ability to think, concentrate	Over the last 2 weeks, how often have you been bothered by any of the following problems? Trouble concentrating on things, such as reading the newspaper or watching television
20513	Depressive Symptoms	Recurrent thoughts of death or suicide ideation, plan for committing suicide	Over the last 2 weeks, how often have you been bothered by any of the following problems? Thoughts that you would be better off dead or of hurting yourself in some way
20506	Anxiety Symptoms	Nervous, anxious or on edge	Over the last 2 weeks, how often have you been bothered by any of the following problems? Feeling nervous, anxious or on edge
20509	Anxiety Symptoms	Uncontrollable worrying	Over the last 2 weeks, how often have you been bothered by any of the following problems? Not being able to stop or control worrying
20515*	Anxiety Symptoms	Trouble relaxing	Over the last 2 weeks, how often have you been bothered by any of the following problems? Trouble relaxing
20505*	Anxiety Symptoms	Irritable	Over the last 2 weeks, how often have you been bothered by any

Continued on next page

Table 1 – continued from previous page

Field	Symptom Class	Symptom	Question
			of the following problems? Becoming easily annoyed or irritable
20520	Anxiety Symptoms	Worrying about different things	Over the last 2 weeks, how often have you been bothered by any of the following problems? Worrying too much about different things
20512	Anxiety Symptoms	Foreboding	Over the last 2 weeks, how often have you been bothered by any of the following problems? Feeling afraid as if something awful might happen
20516	Anxiety	Restlessness	Over the last 2 weeks, how often have you been bothered by any of the following problems? Being so restless that it is hard to sit still
20458	Happiness and subjective well-being	General Happiness	In general, how happy are you?
20460	Happiness and subjective well-being	Belief that own life is meaningful	To what extent do you feel your life to be meaningful?

* 3 symptom variables excluded from the final factor analysis model

Table 4: The relationship between depSympt and the current depressive symptoms variables used in its creation by Jermy et al. [2020].

Field	Symptom	depSympt mean	depSympt sd	p-value
20458	General happiness			< 2.2e-16
	Extremely happy	-1.17	0.75	
	Very happy	-0.39	0.68	
	Moderately happy	0.51	0.74	
	Moderately unhappy	1.64	0.71	
	Very unhappy	2.21	0.91	
	Extremely unhappy	2.80	0.97	

Continued on next page

Table 4 – continued from previous page

Field	Symptom	depSympt mean	depSympt sd	p-value
20460	Life feels meaningful			< 2.2e-16
	Not at all	1.33	1.31	
	A little	1.17	0.95	
	A moderate amount	0.52	0.85	
	Very much	-0.26	0.81	
	An extreme amount	-0.68	0.94	
20506	Nervousness/ anxiety			< 2.2e-16
	Not at all	-0.37	0.80	
	Several days	0.81	0.70	
	> 1/2 the days	1.56	0.80	
	Nearly every day	1.99	0.94	
20508	Trouble concentrating			< 2.2e-16
	Not at all	-0.27	0.81	
	Several days	1.10	0.62	
	> 1/2 the days	1.89	0.68	
	Nearly every day	2.31	0.89	
20509	Uncontrolled worrying			< 2.2e-16
	Not at all	-0.33	0.80	
	Several days	0.93	0.67	
	> 1/2 the days	1.61	0.74	
	Nearly every day	2.03	0.89	
20510	Feelings of depression			< 2.2e-16
	Not at all	-0.38	0.71	
	Several days	1.20	0.45	
	> 1/2 the days	2.14	0.43	
	Nearly every day	2.77	0.58	
20511	Under or over eating			< 2.2e-16
	Not at all	-0.24	0.85	
	Several days	0.89	0.72	
	> 1/2 the days	1.50	0.79	
	Nearly every day	1.91	0.96	
20512	Feelings of foreboding			< 2.2e-16
	Not at all	-0.22	0.87	
	Several days	0.95	0.74	
	> 1/2 the days	1.62	0.83	
	Nearly every day	2.04	0.95	
20513	Suicidal/ self-harming thoughts			< 2.2e-16
	Not at all	-0.08	0.92	
	Several days	1.75	0.65	
	> 1/2 the days	2.51	0.63	

Continued on next page

Table 4 – continued from previous page

Field	Symptom	depSympt mean	depSympt sd	p-value
	Nearly every day	2.99	0.76	
20514	Anhedonia			< 2.2e-16
	Not at all	-0.32	0.75	
	Several days	1.26	0.46	
	> 1/2 the days	2.04	0.52	
	Nearly every day	2.52	0.79	
20516	Restlessness			< 2.2e-16
	Not at all	-0.15	0.91	
	Several days	1.01	0.77	
	> 1/2 the days	1.70	0.91	
	Nearly every day	1.80	1.11	
20517	Sleep problems			< 2.2e-16
	Not at all	-0.55	0.79	
	Several days	0.37	0.74	
	> 1/2 the days	0.88	0.85	
	Nearly every day	1.21	1.02	
20518	Movement and/or speaking changes			< 2.2e-16
	Not at all	-0.09	0.93	
	Several days	1.44	0.72	
	> 1/2 the days	2.19	0.82	
	Nearly every day	2.27	1.12	
20519	Fatigue			< 2.2e-16
	Not at all	-0.66	0.68	
	Several days	0.46	0.66	
	> 1/2 the days	1.23	0.73	
	Nearly every day	1.66	0.92	
20520	Changes in worry			< 2.2e-16
	Not at all	-0.42	0.78	
	Several days	0.75	0.69	
	> 1/2 the days	1.53	0.77	
	Nearly every day	1.96	0.91	

sd = standard deviation. P-value is from a likelihood ratio test comparing generalised linear models for depSympt with and without the symptom included.
Symptom variables are current symptoms at the time of taking the MHQ.

Table 2: Proportion of variation in liability to depression* explained by six latent depression symptom scores [Jermy et al., 2020], and the correlation of these scores with depSympt. (*depression here is defined using data fields `20446` and `20441`. A case will answer yes to at least one of the following: 1 (`20446`). 'Ever had prolonged feelings of sadness or depression?', and/or, 2 (`20441`). 'Ever had prolonged loss of interest in normal activities?'. $p(\text{case} \mid \text{MHQ responder} + \text{within sample}) = 0.5610$; calculated using sample size $n = 119,690$.

Covariate	Proportion of variation in liability to depression explained (%)	Correlation with depSympt
depSympt	11.06	1.000
Depression	10.72	0.995
Anxiety	9.56	0.883
Subjective wellbeing	8.07	0.842
Psychomotor cognitive	10.44	0.976
Neurovegetative	10.16	0.955

Table 3: Correlation matrix for the six latent depression symptom scores of Jermy et al. [2020] ($n = 119,690$).

	depSympt	Depression	Anxiety	Wellbeing[a]	Psychomotor[b]	Neurovegetative
depSympt	1.000	0.995	0.883	0.842	0.976	0.955
Depression	0.995	1.000	0.863	0.830	0.961	0.939
Anxiety	0.883	0.863	1.000	0.699	0.849	0.808
Wellbeing[a]	0.842	0.830	0.699	1.000	0.798	0.747
Psychomotor[b]	0.976	0.961	0.849	0.798	1.000	0.927
Neurovegetative	0.955	0.939	0.808	0.747	0.927	1.000

depSympt is our selected outcome trait. All depression scores are highly correlated with depSympt.

a: Subjective wellbeing factor of depressive symptoms, defined in main text.

b: Psychomotor cognitive factor of depressive symptoms, defined in main text.

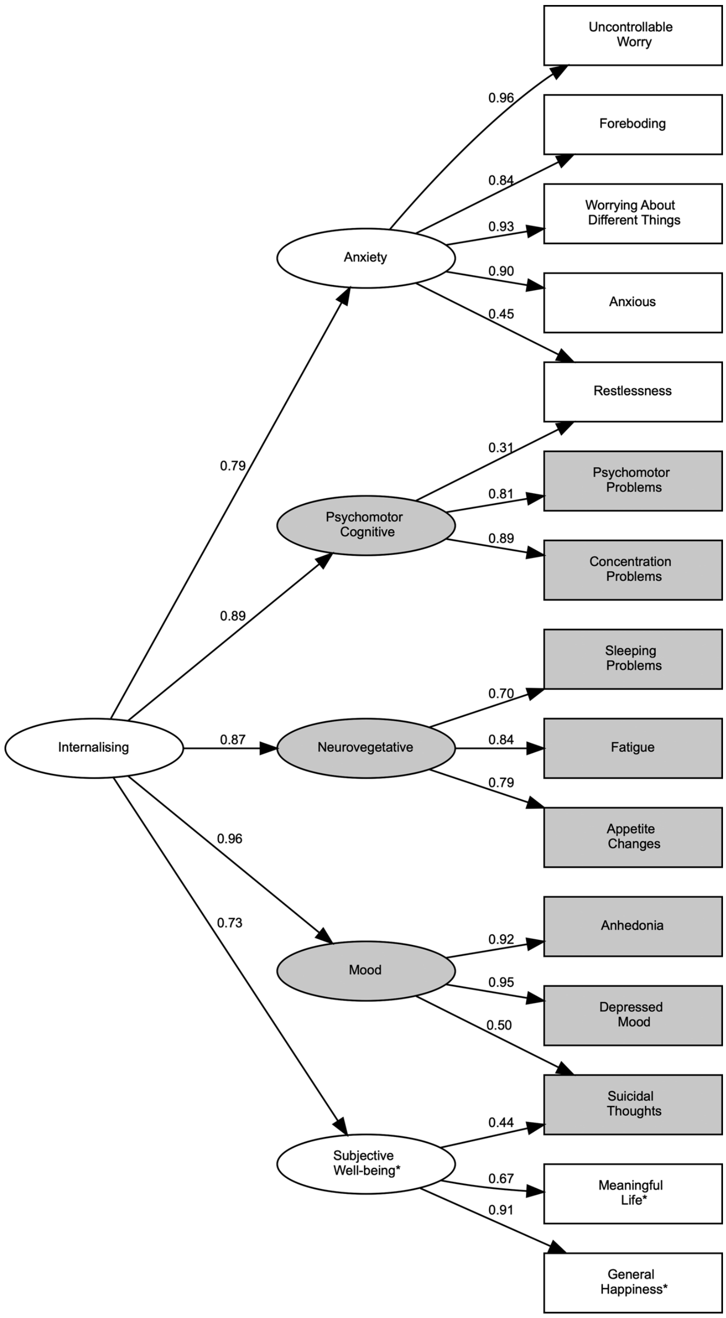

Figure 1: Visualisation for the model of depression symptom scores from Jermy et al. [2020]; exact copy of Figure 2. Note: 'internalising' factor in this plot is called depSympt in this work. Original caption reads: Factor model used to derive the dimensional phenotypes. As is customary in structural equation modelling graphs, circles are factors and squares are the self-reported symptoms. Shaded areas relate to either core MDD symptoms or factors containing a majority of MDD symptoms. Arrows pointing from either one factor to a symptom or a factor to another factor represent the factor loadings. *The items 'General Happiness' and 'Meaningful Life' have been reverse coded such that they explore 'general unhappiness' or 'lack of meaning in one's life'. Subjective well-being, therefore, also corresponds to a 'subjective lack of well-being'. Nomenclature has been retained for the brevity of the labelling.

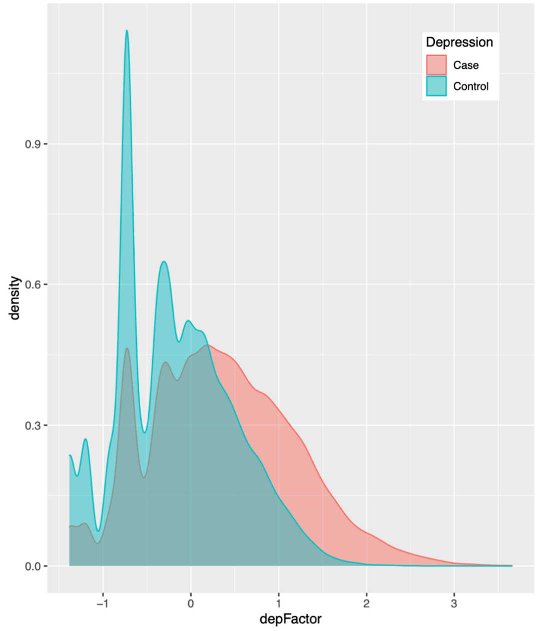

	depFactor			
	N	Mean	SD	Median
MDD status				
Case	67,144	0.35	0.84	0.30
Control	52,545	-0.18	0.66	-0.25
Overall	119,689	0.12	0.81	0.06

Figure 2: depSympt density plot by major depressive disorder (MDD) status with descriptive statistics. Permutation-based hypothesis tests were performed for all listed depSympt descriptive statistics, testing the null of case-control equivalence. $100,000$ permutations were used. Empirical p-values for mean, median and standard deviation (SD) were all 0, meaning under the null no case-control differences as extreme as those observed occurred from $100,000$ samples.

1.2 Phenotype adjustment

In each of the 17 interaction analyses, the outcome trait is adjusted for the fixed effects of the following variables: age (at interview) (data field 21003), sex (data field 31), year of birth (data field 34), assessment centre at which participant consented (data field 54), genotype batch (data field 22000), principal components 1 to 15, Townsend deprivation index at recruitment (TDI, data field 189), average sleep duration (data field 1160), childhood trauma (as a continuous summary variable created by Pitharouli et al. [2021]), 11 variables from the MHQ relating to traumatic and stressful events (not captured by the childhood trauma variable), one measure of body composition and one measure of activity level. The outcome trait is also further adjusted for the fixed effects of the covariate trait under investigation (if it is not listed above).

The 11 variables from the MHQ relating to traumatic and stressful events are: 'Been in a confiding relationship as an adult' (data field 20522), 'Physical violence by partner or ex (adult)' (data field 20523), 'Belittlement by partner or ex (adult)' (data field 20521), 'Sexual interference partner or ex (adult)' (data field 20524), 'Able to pay rent/mortgage as an adult' (data field 20525), 'Victim of sexual assault' (data field 20531), 'Victim of physically violent crime' (data field 20529), 'Been in serious accident believed to be life threatening?' (data field 20526), 'Witnessed sudden violent death' (data field 20530), 'Diagnosed with life-threatening illness' (data field 20528) and 'Been involved in combat or in a war-zone' (data field 20527). The variables capture events occurring in adulthood or events that are not captured by the childhood trauma summary variable.

When the covariate trait is a measure of body composition (BMI, waist circumference and waist to hip ratio) it is the one measure of body composition that is adjusted for. When the covariate trait is not a measure of body composition, BMI is adjusted for.

When the covariate trait is a measure of activity level (MET walk, MET moderate, MET vigorous and MET total) it is the one measure of activity level that is adjusted for. When the covariate trait not a measure of activity level, MET total is adjusted for.

The UK Biobank data fields the body composition and activity level variables are as follows: BMI (21001), waist circumference (48), waist to hip ratio is waist circumference (48) divided by hip circumference (49), MET walk (22037), MET moderate (22038), MET vigorous (22039) and MET total (22040).

Finally, when the covariate trait is a biomarker there are additional adjustments in the depSympt fixed effects model. When the covariate trait was C-reactive protein (CRP-data field 30710), LDL (data field 30780), Triglycerides (data field 30870) or vitamin D (data field 30890) we adjusted for all other biomarkers except for HDL (data field 30760), and when HDL was the covariate trait we adjusted for all biomarkers. HDL had more missing data than CRP, LDL, Triglycerides and vitamin D. To maximise the available sample for the interaction analyses of CRP, LDL, Triglycerides and vitamin D

we therefore decided not to adjust for HDL, putting them into a different analysis group (with n=83,489) to HDL (n=76,246).

Table 5 describes the variables included in the fixed effects model for depSympt as part of the interaction analysis with each covariate trait in turn. The covariate is adjusted for all the same variables as depSympt, except for itself.

Average sleep duration is known to have a non-linear relationship with depression symptoms, with both too little and too much sleep being symptoms of depression. It is possible that other continuous traits also have a non-linear relationship with depSympt. Therefore, with the exception of the principal components, all continuous fixed effects variables used are allowed to have a non-linear relationship with depSympt and the covariate traits by using fractional polynomials (FPs) [Royston and Altman, 1994]. To do this we used the `R` package `mfp` [Benner and Ambler, 2015] within a generalised linear model (`stats::glm` [R Core Team, 2020]). When specified, this package explores the relationship between an outcome and a continuous covariate by testing for suitable (power and log based) transformations of the covariate that best explain the relationship between this variable and the outcome. We allow up to two fractional polynomial terms (transformations of each continuous covariate) to be included. It is possible for the `mfp` package to select no relationship between a variable and the outcome. If this occurs we still include the untransformed variable as a linear term in the final fixed effects model. See Benner and Ambler [2015] for full details on which power transformations are tested for, and how, when using fractional polynomials.

Prior to transformation via fractional polynomials, all biomarkers except LDL were log-transformed. Log-transforming biomarkers is typically done and after inspecting the distribution plots of the untransformed and log-transformed biomarkers (see Supplementary Figures 20 - 24) we concluded that only LDL had a distribution obviously closer to normality on the untransformed scale.

Table 5: Additional variables used in fixed effects adjustment of depSympt for all interaction analyses (defined by the covariate trait).

Covariate trait	Additional variables for depSympt fixed effects models†
BMI	BMI, MET (total)
TDI	BMI, MET (total)
Sleep	BMI, MET (total)
Childhood trauma	BMI, MET (total)
MET (total)	BMI, MET (total)
MET (walk)	BMI, MET (walk)
MET (mod)	BMI, MET (mod)
MET (vig)	BMI, MET (vig)
Waist circumference	Waist circumference, MET (total)
Waist to hip ratio	Waist to hip ratio, MET (total)
log-CRP	log-CRP, LDL, log-triglycerides, log-vitamin D, BMI, MET (total)
LDL	log-CRP, LDL, log-triglycerides, log-vitamin D, BMI, MET (total)
log-Triglycerides	log-CRP, LDL, log-triglycerides, log-vitamin D, BMI, MET (total)
log-Vitamin D	log-CRP, LDL, log-triglycerides, log-vitamin D, BMI, MET (total)
log-HDL	log-HDL, log-CRP, LDL, log-triglycerides, log-vitamin D, BMI, MET (total)
Neuroticism	Neuroticism, BMI, MET (total)
Smoking	Smoking, BMI, MET (total)

†Variables adjusted for in addition to: age, sex, year of birth, assessment centre, genotype batch, principal components 1 to 15, TDI, average sleep duration, childhood trauma and 11 adult trauma and stressful life event items from the MHQ.

Sleep = average sleep duration. MET (total) = Summed MET minutes per week all activities. MET (walk) = Summed MET minutes per week walking. MET (mod) = Summed MET minutes per week moderate. MET (vig) = Summed MET minutes per week vigorous. CRP = C-reactive protein.

1.3 Multivariate reaction norm model

1.3.1 Model introduction

Developed within studies of ecology and agriculture, the reaction norm (RN) is a function characterising phenotypic plasticity; that is, how the observed phenotype of a given genotype (individual) changes when moving along an environmental gradient. Non-parallel RNs indicate the presence of genotype-environment interactions. Population properties can be studied using a collection, or bundle, of RNs via a RN model (RNM). RNMs estimate: 1. the average outcome trait value for a given covariate trait value (the estimated trend between trait and environment regardless of genotype via a fixed effects model), and 2. the residual outcome trait variability for each environmental value allowing investigation of genotype-environment interactions via a random effects model (non-parallel RNs due to the presence of gene-environment interactions will produce heterogeneity in outcome variance across the environmental gradient). RNMs are therefore a type of mixed effects model capturing average trend via the fixed effects model and residual heterogeneity via the random effects model.

RNs can be obtained experimentally within many plant and animal studies. This is not generally possible for human studies; we do not typically observe an individuals phenotypic response to varying levels of an environmental exposure, whilst controlling for confounders. Instead, a single point on each individuals RN is typically available (we observe one outcome value and one covariate trait for each individual in the study population). However, a RNM can still be constructed using estimated genetic similarities from genome-wide SNP data within a random regression model [Schaeffer, 2004, Jarquin et al., 2014, Ni et al., 2019].

In this approach, after adjusting for average trends in the outcome trait across all genotypes using a linear regression model (the fixed effects model), the variance of the outcome trait (Y) is decomposed into genetic and residual components. The genetic component captures the proportion of variability in Y attributable to the measured genetic variables. This will be the SNP heritability, which is the correlation between the estimated genetic sharing (defined by the genetic relationship matrix) and phenotypic sharing [Hall and Bush, 2016]. In the RNM these variance components are further decomposed such that they are functions of the covariate trait (C), allowing the SNP heritability and the residual variance component for outcome Y to vary with respect to C, thereby incorporating a genome-wide genotype-covariate (G-C) and residual-covariate (R-C) interaction.

Like in the RNMs applied to animal/plant studies, RNMs within human GWAS are looking at the average trend and the trend in outcome variability across a covariate. In controlled experiments we can be certain that the cause of the trend in phenotypic variability is due to G-C interactions. In observational data, where we cannot control for other sources of variation within the study design, this is not certain. Therefore, we need to estimate what proportion of this trend is attributable to genetic and non-genetic

sources by using a measure of genetic similarity.

In the MRNM the correlation between the outcome and covariate trait is modelled. This is done by incorporating a second random effects model for the covariate trait, C. The term multivariate therefore refers to two random effects models being considered jointly; one for Y and one for C. As before we: 1. adjust C for average trends using a fixed effects model, and, 2. decompose the residual variation into genetic and residual components. Unlike for outcome trait Y we do not further decompose these random components, and are therefore estimating the SNP heritability and residual variance component for C. We will discuss in the next section, which mathematically defines the model, how using a random effects models for both Y and C can estimate and control for genetic and residual correlations between the two traits.

1.3.2 Model definition

Focusing on the random effects model, because this is where the interactions are modelled, we assume that Y (C) refers to the outcome (covariate) trait that has been pre-adjusted for fixed effects using a linear model, and then standardised. For completeness, that is for each individual i:

$$Y_i = \frac{Y_i^o - E[Y_i^o|\underline{X}_i = \underline{x}_i, C_i^o = c_i^o]}{\sqrt{Var[Y_i^o|\underline{X}_i = \underline{x}_i, C_i^o = c_i^o]}} \sim N(0,1) \tag{1}$$

and:

$$C_i = \frac{C_i^o - E[C_i^o|\underline{X}_i = \underline{x}_i]}{\sqrt{Var[C_i^o|\underline{X}_i = \underline{x}_i]}} \sim N(0,1) \tag{2}$$

where:

- Y_i^o (C_i^o) is the original outcome (covariate) trait, prior to fixed effects adjustment, for individual i,
- $\underline{X}_i$ is a vector of random variables selected for inclusion in the fixed effects model for both Y and C,
- $\underline{x}_i$ is a vector of observed variables for individual i,
- $E[Y_i^o|\underline{X}_i = \underline{x}_i, C_i^o = c_i^o]$ is the estimated value of Y_i^o from a linear model including predictors $\underline{X}_i$ and C_i^o,
- $Var[Y_i^o|\underline{X}_i = \underline{x}_i, C_i^o = c_i^o]$ is the residual variation from this fixed effects model,
- $E[C_i^o|\underline{X}_i = \underline{x}_i]$ is the estimated value of C_i^o from a linear model including predictors $\underline{X}_i$, and,
- $Var[C_i^o|\underline{X}_i = \underline{x}_i]$ is the residual variation from this fixed effects model.

Why do we use standardised traits? In this paper we have multiple covariate traits, whose G-C and R-C interactions with respect to the outcome trait are explored in separate MRNMs. Standardising the covariate traits allows us to compare their relative importance in explaining the variability in the outcome trait across models. Standardising the outcome trait allows the comparison of the impact of covariate traits across outcome traits. Here, we only have one outcome trait (depSympt), but: 1. results from the model using the standardised outcome trait allows comparison across studies, and, 2. the proportion of the variability in raw depSympt attributable to changes in the standard deviation of the covariate trait can be obtained from a model using standardised depSympt as the outcome (see Supplementary Section 1.2 for details).

For each individual i in a sample of size N, we define the (random effects part of the) MRNM as:

$$\begin{bmatrix} Y_i|C_i = c_i \\ C_i \end{bmatrix} = \begin{bmatrix} \alpha_{0i} + \alpha_{1i}c_i \\ \beta_{0i} \end{bmatrix} + \begin{bmatrix} \tau_{0i} + \tau_{1i}c_i \\ \epsilon_{0i} \end{bmatrix} \tag{3}$$

where:

- $\alpha_{0i} \sim N(0, \sigma^2_{\alpha_0})$ is the random effect coefficient describing the random genetic intercept for Y_i. It is a random variable for the main genetic effect for individual i, describing the relationship between the measured genetic variables for this individual and their standardised deviation from the expected outcome trait value, which does not change with C_i.
- $\alpha_{1i} \sim N(0, \sigma^2_{\alpha_1})$ is the random effect coefficient describing a random genetic slope across C_i for individual i. This is the G-C interaction term, and is a random variable describing the relationship between the measured genetic variables for this individual and their standardised deviation from the expected outcome trait value, which can vary across C_i.
- $\tau_{0i} \sim N(0, \sigma^2_{\tau_0})$ is the random effect coefficient describing the residual random intercept for Y_i. It is a random variable describing the standardised residual deviation from the expected value for this individual, that is not accounted for by the measured genetic variables and which does not vary with C_i.
- $\tau_{1i} \sim N(0, \sigma^2_{\tau_1})$ is the random effect coefficient describing the residual random slope for individual i. It is a random variable describing the standardised residual deviation from the expected value for individual i, that is not accounted for by the measured genetic variables but which does vary with C_i. This is the R-C interaction.
- $\beta_{0i} \sim N(0, \sigma^2_{\beta_0})$ is the random effect coefficient describing the random genetic intercept for C_i. It is a random variable describing the relationship between the measured genetic variables for individual i and their standardised deviation for the covariate trait. The population distribution parameter $\sigma^2_{\beta_0}$ is the SNP heritability for the covariate trait given the fixed effects model.

- $\epsilon_{0i} \sim N(0, \sigma^2_{\epsilon_0})$ is the random effect coefficient describing the residual random intercept for C_i. It is a random variable describing the standardised deviation of the covariate trait for individual i that is not explained by their measured genetic variables.

Note, this is Equation (1) in the main paper.

Here, unlike in a fixed effects model, each individual in the sample has his or her own set of random variables (random effects) to capture heterogeneity. Although each individual has their own random effects, these are assumed to follow the same *population* distribution. Here this distribution is assumed to be multivariate normal with mean equal to zero. Therefore, to parameterise the random effects model, we need to estimate the unknown variance-covariance parameters for the random effects. We shall now show this explicitly by writing the model within the study population. The above random effects model can be written in matrix form for the complete sample as follows:

$$\begin{bmatrix} \underline{Y}|\underline{C} = \underline{c} \\ \underline{C} \end{bmatrix} = \begin{bmatrix} \underline{\alpha}_0 + \underline{\alpha}_1 \underline{c} \\ \underline{\beta}_0 \end{bmatrix} + \begin{bmatrix} \underline{\tau}_0 + \underline{\tau}_1 \underline{c} \\ \underline{\epsilon}_0 \end{bmatrix}$$

such that:

$$\begin{bmatrix} \underline{Y}|\underline{C} = \underline{c} \\ \underline{C} \end{bmatrix} \sim N\Big(\begin{bmatrix} \underline{0} \\ \underline{0} \end{bmatrix}, \Sigma \Big)$$

where:

$$\Sigma = \begin{bmatrix} \Sigma_{\underline{Y}} & \Sigma_{\underline{Y},\underline{C}} \\ \Sigma_{\underline{C},\underline{Y}} & \Sigma_{\underline{C}} \end{bmatrix}$$

As you can see, for a random effects model, estimating the unknown population model parameters are contained within the covariance matrix Σ, which defines the variance-covariance of the conditional outcome trait and the covariate trait for the N individuals in the study population.

Functions defining the covariance matrix

1. Defining $\Sigma_{\underline{Y}}$

$\Sigma_{\underline{Y}}$ is the variance-covariance matrix for $\underline{Y}|\{\underline{C} = \underline{c}\}$, describing the covariance between the standardised residual outcome trait for all individuals, where the ith row and j^{th} column is defined as:

$$\begin{aligned} \Sigma_{\underline{Y}}(i,j) &= Cov[Y_i|\{C_i = c_i\}, Y_j|\{C_j = c_j\}] \\ &= \underline{A}(i,j)\sigma_{g_{Y,i},g_{Y,j}} + \underline{I}(i,j)\sigma_{e_{Y,i},e_{Y,j}} \end{aligned}$$

where $\sigma_{g_{Y,i},g_{Y,j}}$ is the covariance between the random genetic components of trait Y for two values of the covariate trait denoted by c_i and c_j which is:

$$\sigma_{g_{Y,i},g_{Y,j}} = \sigma^2_{\alpha_0} + (c_i + c_j)\sigma_{\alpha_0,\alpha_1} + c_i c_j \sigma^2_{\alpha_1}$$

Similarly, $\sigma_{e_{Y,i},e_{Y,j}}$ is the covariance between the random residual components of trait Y for two values of the covariate trait denoted by c_i and c_j which is:

$$\sigma_{e_{Y,i},e_{Y,j}} = \sigma^2_{\tau_0} + (c_i + c_j)\sigma_{\tau_0,\tau_1} + c_i c_j \sigma^2_{\tau_1}$$

$\underline{A}(i,j)$ the value contained within the ith row and j^{th} column of the GRM, corresponding to the average (measured) genetic sharing between individuals i and j. $\underline{A}(i,i) = 1$. $\underline{I}(i,j)$ is the value in the ith row and j^{th} column of the identity matrix. This will be 0 when $i \neq j$, meaning we assume there is no environmental sharing between individuals in this model.

We note that the above leads to a conditional variance estimate for trait Y_i of:

$$Var[Y_i|C_i = c_i] = \sigma^2_{\alpha_0} + 2c_i\sigma_{\alpha_0,\alpha_1} + c_i^2\sigma^2_{\alpha_1} + \sigma^2_{\tau_0} + 2c_i\sigma_{\tau_0,\tau_1} + c_i^2\sigma^2_{\tau_1}$$

and, assuming $E[C_i] = 0$ and $Var[C_i] = 1$, an unconditional variance estimate of:

$$Var[Y_i] = \sigma^2_{\alpha_0} + \sigma^2_{\alpha_1} + \sigma^2_{\tau_0} + \sigma^2_{\tau_1}$$

for all individuals in the population.

Since $Var[Y_i] = 1$, the variance estimates for $\sigma^2_{\alpha_0}$, $\sigma^2_{\alpha_1}$, $\sigma^2_{\tau_0}$ and $\sigma^2_{\tau_1}$ represent a measure of the importance for that variance component (main genetic, G-C, main residual and R-C) in explaining the variability of the outcome trait in the population.

2. Defining $\Sigma_{\underline{Y},\underline{C}}$

$\Sigma_{\underline{Y},\underline{C}} = \Sigma^T_{\underline{C},\underline{Y}}$ is the covariance matrix for $\underline{Y}|\{\underline{C} = \underline{c}\}$ and $\underline{C}$, where the ith row and j^{th} column is defined as:

$$\begin{aligned}\Sigma_{\underline{Y},\underline{C}}(i,j) &= Cov[Y_i|\{C_i = c_i\}, C_j] \\ &= \underline{A}(i,j)\Big(\sigma_{\alpha_0,\beta_0} + c_i\sigma_{\alpha_1,\beta_0}\Big) + \underline{I}(i,j)\Big(\sigma_{\tau_0,\epsilon_0} + c_i\sigma_{\tau_1,\epsilon_0}\Big)\end{aligned}$$

$\Sigma_{\underline{Y},\underline{C}}(i,j)$ contains the covariance between the conditional outcome trait for individual i (given we observe their covariate trait) and the covariate trait for individual j. It is the sum of the genetic and the residual covariances between the traits when $i = j$. It is assumed that when individual $i \neq j$, $\Sigma_{\underline{Y},\underline{C}}(i,j)$ is equal to the genetic covariance between the traits only. That is, residual variation is explained by independent variables for each individual, and there are no un-modelled correlations, within the environment for example, between individuals that explain any covariation between the outcome of one individual and the covariate of another (we note that there is a modelled relationship between the variance of Y_i and C_i).

The genetic covariance is a function of the measured trait value (c_i), the measured genetic sharing and the population random effects covariances $\sigma_{\alpha_0,\beta_0}$ and $\sigma_{\alpha_1,\beta_0}$ (which are to be estimated). $\sigma_{\alpha_0,\beta_0}$ defines how the main genetic effect for $Y|C = c$ covaries with the main genetic effect of C, and $\sigma_{\alpha_1,\beta_0}$ defines how the G-C interaction effect for $Y|C = c$ covaries with the main genetic effect of C.

The residual covariance, only used within an individual, is a function of the measured trait value (c_i) and the population random effects covariances $\sigma_{\tau_0,\epsilon_0}$ and $\sigma_{\tau_1,\epsilon_0}$ (which are to be estimated). $\sigma_{\tau_0,\epsilon_0}$ defines how the main residual random effect for $Y|C = c$ covaries with the main residual effect of C, and $\sigma_{\tau_1,\epsilon_0}$ defines how the R-C interaction random effect for $Y|C = c$ covaries with the main genetic effect of C, but only within an individual (not between individuals). Estimating these parameters allows for residual covariance between outcome and the covariate trait that is otherwise un-modelled. If these covariances were not included, genetic interaction variances may be inflated.

3. Defining $\Sigma_{\underline{C}}$

$\Sigma_{\underline{C}}$ is the variance-covariance matrix for $\underline{C}$, where the ith row and j^{th} column is defined as:

$$\begin{aligned}\Sigma_{\underline{C}}(i,j) &= Cov[C_i, C_j] \\ &= \underline{A}(i,j)\sigma^2_{\beta_0} + \underline{I}(i,j)\sigma^2_{\epsilon_0}\end{aligned}$$

These variance-covariance matrices defining Σ can also be written in matrix form. Please see Ni et al. [2019], the methods paper which first described the MRNM within human GWAS, for details of this. For information about the (restricted) maximum likelihood estimation process for these covariance parameters please see Lee and van der Werf [2016], which describes the `mtg2` software package and its corresponding manual found here: `https://sites.google.com/site/honglee0707/mtg2`.

Here, we just note that the MRNM is parameterised by estimating the following covariance matrix between the random effects (RE), which represent sources of (co)variation for $\underline{Y}|\underline{C} = \underline{c}$ and $\underline{C}$:

$$\Sigma_{RE} = \begin{bmatrix} \sigma^2_{\alpha_0} & \sigma_{\alpha_0,\alpha_1} & \sigma_{\alpha_0,\tau_0} & \sigma_{\alpha_0,\tau_1} & \sigma_{\alpha_0,\beta_0} & \sigma_{\alpha_0,\epsilon_0} \\ \sigma_{\alpha_0,\alpha_1} & \sigma^2_{\alpha_1} & \sigma_{\alpha_1,\tau_0} & \sigma_{\alpha_1,\tau_1} & \sigma_{\alpha_1,\beta_0} & \sigma_{\alpha_1,\epsilon_0} \\ \sigma_{\alpha_0,\tau_0} & \sigma_{\alpha_1,\tau_0} & \sigma^2_{\tau_0} & \sigma_{\tau_0,\tau_1} & \sigma_{\tau_0,\beta_0} & \sigma_{\tau_0,\epsilon_0} \\ \sigma_{\alpha_0,\tau_1} & \sigma_{\alpha_1,\tau_1} & \sigma_{\tau_0,\tau_1} & \sigma^2_{\tau_1} & \sigma_{\tau_1,\beta_0} & \sigma_{\tau_1,\epsilon_0} \\ \sigma_{\alpha_0,\beta_0} & \sigma_{\alpha_1,\beta_0} & \sigma_{\tau_0,\beta_0} & \sigma_{\tau_1,\beta_0} & \sigma^2_{\beta_0} & \sigma_{\beta_0,\epsilon_0} \\ \sigma_{\alpha_0,\epsilon_0} & \sigma_{\alpha_1,\epsilon_0} & \sigma_{\tau_0,\epsilon_0} & \sigma_{\tau_1,\epsilon_0} & \sigma_{\beta_0,\epsilon_0} & \sigma^2_{\epsilon_0} \end{bmatrix}$$

$$= \begin{bmatrix} \sigma^2_{\alpha_0} & \sigma_{\alpha_0,\alpha_1} & 0 & 0 & \sigma_{\alpha_0,\beta_0} & 0 \\ \sigma_{\alpha_0,\alpha_1} & \sigma^2_{\alpha_1} & 0 & 0 & \sigma_{\alpha_1,\beta_0} & 0 \\ 0 & 0 & \sigma^2_{\tau_0} & \sigma_{\tau_0,\tau_1} & 0 & \sigma_{\tau_0,\epsilon_0} \\ 0 & 0 & \sigma_{\tau_0,\tau_1} & \sigma^2_{\tau_1} & 0 & \sigma_{\tau_1,\epsilon_0} \\ \sigma_{\alpha_0,\beta_0} & \sigma_{\alpha_1,\beta_0} & 0 & 0 & \sigma^2_{\beta_0} & 0 \\ 0 & 0 & \sigma_{\tau_0,\epsilon_0} & \sigma_{\tau_1,\epsilon_0} & 0 & \sigma^2_{\epsilon_0} \end{bmatrix}$$

Although some of these model parameters are variances, and so should be > 0, the algorithm estimating these parameters does not know that they should be constrained. Negative variance estimates are therefore possible. Typically this is just an underestimation of a small, or zero, variance. We therefore calculate confidence intervals for variance parameters. A variance estimate with a 95% confidence interval that overlaps with zero indicates a lack of confidence that the random effect it corresponds to is useful in explaining phenotypic variation.

The variance-covariance random effects parameters

In this work some covariance parameters are assumed to be zero, as indicated in the definition of Σ_{RE} above. In particular, within each trait and between traits, we assume that the genetic random effects are uncorrelated with the residual random effects.

$\sigma^2_{\beta_0}$ ($= Var[\beta_0]$) is the SNP heritability for the standardised covariate trait, C. $\sigma^2_{\epsilon_0}$ is the proportion of variability in C not captured by the measured genetic variables; it is residual.

Using the above MRNM, the (measured) genetic and residual variance components for Y_i are a function of C_i, such that:

$$Var[Y_i|C_i = c_i] = V_{G_i|C_i=c_i} + V_{R_i|C_i=c_i} \tag{4}$$

$$V_{G_i|C_i=c_i} = \sigma^2_{\alpha_0} + 2c_i\sigma_{\alpha_0,\alpha_1} + c_i^2\sigma^2_{\alpha_1}$$
$$V_{R_i|C_i=c_i} = \sigma^2_{\tau_0} + 2c_i\sigma_{\tau_0,\tau_1} + c_i^2\sigma^2_{\tau_1}$$

for all individuals in the population. $V_{G_i|C_i=c_i}$ ($V_{R_i|C_i=c_i}$) is the genetic (residual) variance component for $Y_i|\{C_i = c_i\}$. The MRNM in Equation (3) specifies that the phenotypic variability in Y_i is a degree 2 polynomial function, which will equal $\sigma^2_{\alpha_0} + \sigma^2_{\tau_0}$ when $c_i = 0$ (the mean). Therefore, $\sigma^2_{\alpha_0}$ can be thought of as the polygenic variance component of Y for individuals with the covariate trait equal to the expected from the fixed effects model. $\sigma^2_{\alpha_1}$ and $\sigma_{\alpha_0,\alpha_1}$ determine the change in $V_{G_i|C_i=c_i}$ for different values of C_i with larger (absolute) values for these variance-covariance model parameters indicating a larger differences in the polygenic variance component of Y for larger deviations in the covariate trait from its mean.

$\sigma^2_{\alpha_1}$ in particular is an important measure for strength of the G-C interaction, highlighted by its role in the unconditional variance of Y_i defined as:

$$Var[Y_i] = V_{G_i} + V_{R_i} = 1$$

$$V_{G_i} = \sigma^2_{\alpha_0} + \sigma^2_{\alpha_1}$$
$$V_{R_i} = \sigma^2_{\tau_0} + \sigma^2_{\tau_1}$$

(Note- this is Equation 2 in the main paper.)
$\sigma^2_{\alpha_1}$ is part of the SNP heritability for Y determined by variation in C. The larger the $\sigma^2_{\alpha_1}$ value, the more important the G-C interaction is in explaining the variability observed in the standardised outcome trait.

Similarly, $V_{R_i|C_i=c_i}$ is the residual variance component for $Y_i|\{C_i = c_i\}$, which will equal $\sigma^2_{\tau_0}$ when $C = 0$. The variance-covariance parameters $\sigma^2_{\tau_1}$ and σ_{τ_0,τ_1} determine the change in $V_{R_i|C_i=c_i}$ for changes in C_i, with $\sigma^2_{\tau_1}$ being a measure of the importance of the R-C interaction in explaining the variability in Y_i.

Correlation between Y_i and C_i is incorporated into the model by allowing non-zero covariance parameters between the genetic components of the 2 traits ($\sigma_{\alpha_0,\beta_0}$ and $\sigma_{\alpha_1,\beta_0}$), and the residual components of the two traits ($\sigma_{\tau_0,\epsilon_0}$ and $\sigma_{\tau_1,\epsilon_0}$). This is an important advantage of the MRNM because not accounting for genetic and residual correlations between traits could lead to an inflation in the strength of the interactions [Ni et al., 2019].

For completeness, the variance-covariance matrix for $\{Y_i|C_i = c_i\}$ and C_i, denoted by Σ_i, is defined as:

$$\Sigma_i = \tag{5}$$
$$\begin{bmatrix} \sigma^2_{\alpha_0} + 2c_i\sigma_{\alpha_0\alpha_1} + c_i^2 + \sigma^2_{\alpha_1} + \sigma^2_{\tau_0} + 2c_i\sigma_{\tau_0\tau_1} + c_i^2\sigma^2_{\tau_1} & \sigma_{\alpha_0\beta_0} + c_i\sigma_{\alpha_1\beta_0} + \sigma_{\tau_0\epsilon_0} + c_i\sigma_{\tau_1\epsilon_0} \\ \sigma_{\alpha_0\beta_0} + c_i\sigma_{\alpha_1\beta_0} + \sigma_{\tau_0\epsilon_0} + c_i\sigma_{\tau_1\epsilon_0} & \sigma^2_{\beta_0} + \sigma^2_{\epsilon_0} \end{bmatrix}$$

with $\{Y_i|C_i = c_i\}$ and C_i described using the bivariate mixed model:

$$\begin{bmatrix} Y_i|C_i = c_i \\ C_i \end{bmatrix} = \begin{bmatrix} \alpha_{0i} + \alpha_{1i}c_i \\ \beta_{0i} \end{bmatrix} + \begin{bmatrix} \tau_{0i} + \tau_{1i}c_i \\ \epsilon_{0i} \end{bmatrix}$$

(as given in Equation 3), where:

$$\begin{bmatrix} Y_i|C_i = c_i \\ C_i \end{bmatrix} \sim N\left(\begin{bmatrix} 0 \\ 0 \end{bmatrix}, \ \Sigma_i \right)$$

1.4 Meta-analysis methods

1.4.1 Likelihood ratio test

Let p_{jk} be the likelihood ratio p-value for the jth covariate trait ($j = 1, 2, ..., 25$) and the kth subset of data ($k = 1, 2, 3$), when testing the hypothesis:

$$H_0: \sigma^2_{\alpha_{1jk}} = \sigma^2_{\tau_{1jk}} = \sigma_{\alpha_{0jk},\alpha_{1jk}} = \sigma_{\tau_{0jk},\tau_{1jk}} = \sigma_{\alpha_{1jk},\beta_{0jk}} = \sigma_{\tau_{1jk},\epsilon_{0jk}} = 0$$
$$H_1: \text{otherwise}$$

Using Fishers method [Evangelou and Ioannidis, 2013], the combined/ meta test statistic for covariate trait j is:

$$\chi^2_j = -2\sum_{k=1}^{K} log(p_{jk})$$

where $K = 3$, and is the number of datasets to combine, and degrees of freedom, df, equals $2K$. The meta p-value is then calculated using the χ^2 distribution. An example of the `R` code is:

```
pchisq(q = ts_j, df=6, lower.tail=F)
```

where `ts_j` equals χ^2_j above.

1.4.2 Variance-covariance model parameters: estimates and standard errors

The random effects part of the MRNM is parameterised by variance-covariance parameters that are estimated in each data subset, along with standard errors. To combine these parameter estimates and standard errors to obtain meta-analysed estimates we used the following method. Taking $\sigma^2_{\alpha_{0j}}$ as an example, where j denotes the jth covariate trait, we use:

$$\hat{\sigma}^2_{\alpha_{0j}} = \frac{\sum_{k=1}^{K} \hat{\sigma}^2_{\alpha_{0jk}} SE(\hat{\sigma}^2_{\alpha_{0jk}})^{-1}}{\sum_{k=1}^{K} SE(\hat{\sigma}^2_{\alpha_{0jk}})^{-1}}$$

to obtain the meta-analysed model parameter estimate, and:

$$SE(\hat{\sigma}_{\alpha_{0j}}) = \frac{1}{\sum_{k=1}^{K} SE(\hat{\sigma}^2_{\alpha_{0jk}})^{-1}}$$

to obtain the meta-analysed standard error for the model parameter. This is a fixed effects meta-analysis method used by Ni et al. [2019]. It provides a weighted mean

of the parameter estimates giving more weight to data subsets with smaller standard error estimates [Hedges and Vevea, 1998]. Wald confidence intervals are presented (i.e. using a normal distribution) as is done in meta-analysis packages such as `R::metafor` [Viechtbauer, 2010].

1.4.3 Genetic, residual and total variance components for standardised residual depSympt as a function of the covariate trait: estimate and SEs

Assume that we have adjusted both depSympt and the jth covariate trait for their respective fixed effects model, and then standardised, such that for an individual i:

- the outcome trait we are considering is Y_i as defined in Equation (1), and,
- the covariate trait we are considering is C_{ij} as defined in Equation (2).

For a given individual i, it is useful to understand the estimated relationship between expected variability in standardised residual depSympt (Y_i) and the standardised residual covariate trait (C_{ij}), and to break this relationship into the genetic variance component and the residual variance component, as well as obtain standard errors (SEs) for these variance components across C_{ij}. Plotting these relationships (as we have done in Figure 3 presented within the main text for average sleep duration, or for the other covariates considered in Supplementary Figures 27 - 37), allows researchers to better understand the MRNM output, including the relative contributions to total variation from the genetic component compared to the residual, how this changes across the covariate trait and if confidence intervals overlap with each other, or with 0.

Focusing on the part of the MRNM considered here which defined Y_i, we recall that:

$$Y_i|\{C_{ij} = c_{ij}\} = \alpha_{0i} + \alpha_{1i}c_{ij} + \tau_{0i} + \tau_{1i}c_{ij}$$

where the random effects follow a multivariate normal distribution, with zero-mean and covariances between genetic and nongenetic random effects fixed at 0. Using this we can write the following equation for the variance of $Y_i|\{C_{ij} = c_{ij}\}$:

$$\begin{aligned} V_{Y_i|C_{ij}=c_{ij}} &= Var[Y_i|C_{ij} = c_{ij}] \\ &= E[(Y_i|C_{ij} = c_{ij})^2] - (E[Y_i|C_{ij} = c_{ij}])^2 \\ &= E[(Y_i|C_{ij} = c_{ij})^2] \\ &= V_{G_i|C_{ij}=c_{ij}} + V_{R_i|C_{ij}=c_{ij}} \end{aligned}$$

where:

$$V_{G_i|C_{ij}=c_{ij}} = \sigma^2_{\alpha_0} + 2c_{ij}\sigma_{\alpha_0,\alpha_1} + c^2_{ij}\sigma^2_{\alpha_1}$$

is the genetic variance component for conditional Y_i, and:

$$V_{R_i|C_{ij}=c_{ij}} = \sigma^2_{\tau_0} + 2c_{ij}\sigma_{\tau_0,\tau_1} + c^2_{ij}\sigma^2_{\tau_1}$$

is the residual variance component for conditional Y_i (as defined in the MRNM definition section). We note the above is not considering correlation between individuals under-study, and rather focuses within a given individual. An estimate for the total, genetic and residual variance components will be obtained using: $[\hat{\sigma}^2_{\alpha_0}, \hat{\sigma}_{\alpha_0,\alpha_1}, \hat{\sigma}^2_{\alpha_1}, \hat{\sigma}^2_{\tau_0}, \hat{\sigma}_{\tau_0,\tau_1}, \hat{\sigma}^2_{\tau_1}]$, which are variance component estimates outputted by the MRNM (mtg2 package [Lee and van der Werf, 2016]).

Additionally, the inverse Fisher information matrix is outputted by mtg2. This matrix is used to estimate the standard errors of the estimated variance components. Extracting the elements from this matrix relating to the variance components required, and using the delta method, the standard error for the genetic variance component for Y_i given $C_{ij} = c_{ij}$ is given by:

$$SE(\hat{V}_{G_i|C_{ij}=c_{ij}}) = \nabla^T_{V_{G_i|C_{ij}=c_{ij}}} \hat{\Sigma}_{V_{G|C_j=c_j}} \nabla_{V_{G_i|C_{ij}=c_{ij}}}$$

where:

$$\nabla_{V_{G_i|C_{ij}=c_{ij}}} = \begin{bmatrix} 1 \\ 2c_{ij} \\ c^2_{ij} \end{bmatrix}$$

and:

$$\hat{\Sigma}_{V_{G|C_j=c_j}} = \begin{bmatrix} Var[\hat{\sigma}^2_{\alpha_0}] & Cov[\hat{\sigma}^2_{\alpha_0}, \hat{\sigma}_{\alpha_0,\alpha_1}] & Cov[\hat{\sigma}^2_{\alpha_0}, \hat{\sigma}^2_{\alpha_1}] \\ Cov[\hat{\sigma}_{\alpha_0,\alpha_1}, \hat{\sigma}^2_{\alpha_0}] & Var[\hat{\sigma}_{\alpha_0,\alpha_1}] & Cov[\hat{\sigma}_{\alpha_0,\alpha_1}, \hat{\sigma}^2_{\alpha_1}] \\ Cov[\hat{\sigma}^2_{\alpha_1}, \hat{\sigma}^2_{\alpha_0}] & Cov[\hat{\sigma}^2_{\alpha_1}, \hat{\sigma}_{\alpha_0,\alpha_1}] & Var[\hat{\sigma}^2_{\alpha_1}] \end{bmatrix}$$

Similarly, the standard error for the residual variance component for Y_i given $C_{ij} = c_{ij}$ is given by:

$$SE(\hat{V}_{R_i|C_{ij}=c_{ij}}) = \nabla^T_{V_{R_i|C_{ij}=c_{ij}}} \hat{\Sigma}_{V_{R|C_j=c_j}} \nabla_{V_{R_i|C_{ij}=c_{ij}}}$$

where:

$$\nabla_{V_{R_i|C_{ij}=c_{ij}}} = \begin{bmatrix} 1 \\ 2c_{ij} \\ c^2_{ij} \end{bmatrix}$$

and:

$$\hat{\Sigma}_{V_{R|C_j=c_j}} = \begin{bmatrix} Var[\hat{\sigma}^2_{\tau_0}] & Cov[\hat{\sigma}^2_{\tau_0}, \hat{\sigma}_{\tau_0,\tau_1}] & Cov[\hat{\sigma}^2_{\tau_0}, \hat{\sigma}^2_{\tau_1}] \\ Cov[\hat{\sigma}_{\tau_0,\tau_1}, \hat{\sigma}^2_{\tau_0}] & Var[\hat{\sigma}_{\tau_0,\tau_1}] & Cov[\hat{\sigma}_{\tau_0,\tau_1}, \hat{\sigma}^2_{\tau_1}] \\ Cov[\hat{\sigma}^2_{\tau_1}, \hat{\sigma}^2_{\tau_0}] & Cov[\hat{\sigma}^2_{\tau_1}, \hat{\sigma}_{\tau_0,\tau_1}] & Var[\hat{\sigma}^2_{\tau_1}] \end{bmatrix}$$

The standard error for the total variability for Y_i given $C_{ij} = c_{ij}$ is given by:

$$SE(\hat{V}_{Y_i|C_{ij}=c_{ij}}) = \nabla^T_{V_{Y_i|C_{ij}=c_{ij}}} \hat{\Sigma}_{V_{Y|C_j=c_j}} \nabla_{V_{Y_i|C_{ij}=c_{ij}}}$$

where:

$$\nabla_{V_{Y_i|C_{ij}=c_{ij}}} = \begin{bmatrix} 1 \\ 2c_{ij} \\ c_{ij}^2 \\ 1 \\ 2c_{ij} \\ c_{ij}^2 \end{bmatrix}$$

and:

$$\hat{\Sigma}_{V_{Y|C_j=c_j}} = \begin{bmatrix} \hat{\Sigma}_{V_{G|C_j=c_j}} & \hat{\Sigma}_{V_{G|C_j=c_j},V_{R|C_j=c_j}} \\ \hat{\Sigma}^T_{V_{G|C_j=c_j},V_{R|C_j=c_j}} & \hat{\Sigma}_{V_{R|C_j=c_j}} \end{bmatrix}$$

with $\hat{\Sigma}_{V_{G|C_j=c_j}}$ and $\hat{\Sigma}_{V_{R|C_j=c_j}}$ as defined above, and:

$$\hat{\Sigma}_{V_{G|C_j=c_j},V_{R|C_j=c_j}} = \begin{bmatrix} Cov[\hat{\sigma}^2_{\alpha_0}, \hat{\sigma}^2_{\tau_0}] & Cov[\hat{\sigma}^2_{\alpha_0}, \hat{\sigma}_{\tau_0,\tau_1}] & Cov[\hat{\sigma}^2_{\alpha_0}, \hat{\sigma}^2_{\tau_1}] \\ Cov[\hat{\sigma}_{\alpha_0,\alpha_1}, \hat{\sigma}^2_{\tau_0}] & Cov[\hat{\sigma}_{\alpha_0,\alpha_1}, \hat{\sigma}_{\tau_0,\tau_1}] & Cov[\hat{\sigma}_{\alpha_0,\alpha_1}, \hat{\sigma}^2_{\tau_1}] \\ Cov[\hat{\sigma}^2_{\alpha_1}, \hat{\sigma}^2_{\tau_0}] & Cov[\hat{\sigma}^2_{\alpha_1}, \hat{\sigma}_{\tau_0,\tau_1}] & Cov[\hat{\sigma}^2_{\alpha_1}, \hat{\sigma}^2_{\tau_1}] \end{bmatrix}$$

As previously noted, $\hat{\Sigma}_{V_{G|C_j=c_j}}$, $\hat{\Sigma}_{V_{R|C_j=c_j}}$ and $\hat{\Sigma}_{V_{G|C_j=c_j},V_{R|C_j=c_j}}$ can be extracted from the inverse Fisher information outputted by mtg2.

Due to the large sample size available within the UK Biobank, in this analysis we split our study sample in three subgroups and ran MRNMs within each group. Therefore we estimated $V_{G_i|C_{ij}=c_{ij}}$, $V_{R_i|C_{ij}=c_{ij}}$ and $V_{Y_i|C_{ij}=c_{ij}}$ and obtained their respective SEs, over a range of c_{ij} values, within each subgroup and then meta-analysed the results.

1.4.4 Re-scaling the proportion of variability in depSympt attributable to C_j: estimates and standard errors

Heritability estimates for an outcome are typically present with/ after adjustment for age, sex, batch effects and PCs. To ensure the proportion of variability in depSympt attributable the considered interaction effects are comparable to heritability estimates, and other variance components estimates in the literature, we transform our interaction variance component estimates to this scale to. These estimates are presented in Table 1 and Figure 2 in the main text. Here we provide the method for obtaining these estimates from MRNM/mtg2 outputs.

Recall that for an individual i, the standardised residual outcome used in the random effects model of the MRNM is defined by:

$$Y_i = \frac{Y_i^o - E[Y_i^o|\underline{X}_i = \underline{x}_i, C_i^o = c_i^o]}{\sqrt{Var[Y_i^o|\underline{X}_i = \underline{x}_i, C_i^o = c_i^o]}} \sim N(0,1)$$

and:

$$Y_i = \alpha_{0i} + \alpha_{1i}C_i + \tau_{0i} + \tau_{1i}C_i$$

where:

- Y_i^o is the original (non-residualised) depSympt random variable,
- $\underline{X}_i$ ($\underline{x}_i$) is a random (observed) vector for variables contained in the fixed effects model,
- C_i^o (c_i^o) is the unadjusted random (observed) covariate trait,
- $E[Y_i^o|\underline{X}_i = \underline{x}_i, C_i^o = c_i^o]$ is the expected value for depSympt from the fixed effects model adjusting for $\{\underline{X}_i = \underline{x}_i, C_i^o = c_i^o\}$,
- $Var[Y_i^o|\underline{X}_i = \underline{x}_i, C_i^o = c_i^o]$ is the variance of the residual random variable in the above mentioned fixed effects model,
- $C_i \sim N(0,1)$ is the standardised residual covariate trait random variable, after fixed effects adjustment, and,
- $\{\alpha_{0i}, \alpha_{1i}, \tau_{0i}, \tau_{1i}\}$ is an individual-specific random effect corresponding to a main genetic effect, and G-C interaction effect, a main residual effect and a R-C effect respectively.

Let us change notation to:

$$E[Y_i^o|\underline{X}_i = \underline{x}_i, C_i^o = c_i^o] = \mu_{Y_i^o|\underline{X}_i=\underline{x}_i, C_i^o=c_i^o}$$

and:

$$Var[Y_i^o|\underline{X}_i = \underline{x}_i, C_i^o = c_i^o] = \sigma^2_{Y^o|\underline{X}=\underline{x}, C^o=c^o}$$

then, re-arranging the above to make Y_i^o the subject of the formula gives:

$$\begin{aligned} Y_i^o &= \mu_{Y_i^o|\underline{X}_i=\underline{x}_i, C_i^o=c_i^o} + \sigma_{Y^o|\underline{X}=\underline{x}, C^o=c^o} Y_i \\ &= \mu_{Y_i^o|\underline{X}_i=\underline{x}_i, C_i^o=c_i^o} + \sigma_{Y^o|\underline{X}=\underline{x}, C^o=c^o} \big(\alpha_{0i} + \alpha_{1i}C_i + \tau_{0i} + \tau_{1i}C_i\big) \end{aligned}$$

Let the variables used within fixed effects adjustment be split into 2 groups:

$$\underline{X}_i = \begin{bmatrix} \underline{X}_{core,i} \\ \underline{X}_{other,i} \end{bmatrix}$$

where age, sex, batch effects and PCs for individual i are contained within $\underline{X}_{core,i}$. Then we can write the fixed effects model as:

$$\mu_{Y_i^o|\underline{X}_i=\underline{x}_i, C_i^o=c_i^o} = \beta_0 + \underline{\beta}_{core}^T \underline{x}_{core,i} + \underline{\beta}_{other}^T \underline{x}_{other,i} + \beta_C c_i^o$$

Then:

$$Y_i^o = \beta_0 + \underline{\beta}_{core}^T \underline{x}_{core,i} + \underline{\beta}_{other}^T \underline{x}_{other,i} + \beta_C c_i^o + \sigma_{Y^o|\underline{X}=\underline{x}, C^o=c^o} \big(\alpha_{0i} + \alpha_{1i}C_i + \tau_{0i} + \tau_{1i}C_i\big)$$

Note, Y_i^o here is technically $Y_i^o|\{\underline{X}_i = \underline{x}_i, C_i^o = c_i^o\}$, and therefore $Y_i^o|\{\underline{X}_{core,i} = \underline{x}_{core,i}\}$ can be approximated by:

$$
\begin{aligned}
Y_i^o &= \beta_0^{'} + \underline{\beta}_{core}^T \underline{x}_{core,i} + \underline{\beta}_{other}^T \underline{X}_{other,i} + \beta_C C_i^o + \sigma_{Y^o|\underline{X}=\underline{x},C^o=c^o} \big(\alpha_{0i} + \alpha_{1i} C_i + \tau_{0i} + \tau_{1i} C_i\big) \\
&\approx \beta_0^{'} + \underline{\beta}_{core}^T \underline{x}_{core,i} + \epsilon_i
\end{aligned}
$$

where $\beta_0^{'}$ is an updated intercept term defined as:

$$
\beta_0^{'} = \beta_0 + \underline{\beta}_{other}^T E[\underline{X}_{other,i}]
$$

and ϵ_i is the residual random variable from the fixed effects model for depSympt only adjusting for the core variables (age, sex, batch effects and PCs), such that:

$$
\begin{aligned}
\epsilon_i &\approx \underline{\beta}_{other}^T \big(\underline{X}_{other,i} - E[\underline{X}_{other,i}]\big) + \beta_C C_i^o + \sigma_{Y^o|\underline{X}=\underline{x},C^o=c^o} \big(\alpha_{0i} + \alpha_{1i} C_i + \tau_{0i} + \tau_{1i} C_i\big) \\
&\sim N(0, \sigma^2_{Y^o|\underline{X}_{core}=\underline{x}_{core}})
\end{aligned}
$$

This means that:

$$
\begin{aligned}
&Var[Y_i^o|\underline{X}_{core,i} = \underline{x}_{core,i}] \\
&= \sigma^2_{Y^o|\underline{X}_{core}=\underline{x}_{core}} \\
&\approx Var[\underline{\beta}_{other}^T \big(\underline{X}_{other,i} - E[\underline{X}_{other,i}]\big)] + \beta_C^2 + \sigma^2_{Y^o|\underline{X}=\underline{x},C^o=c^o} \big(\sigma^2_{\alpha_0} + \sigma^2_{\alpha_1} + \sigma^2_{\tau_0} + \sigma^2_{\tau_1}\big)
\end{aligned}
$$

and therefore, the proportion of variability in depSympt (adjusted for age, sex, batch effects and PCs) attributable to:

- the main genetic effect $= \frac{\sigma^2_{Y^o|\underline{X}=\underline{x},C^o=c^o}}{\sigma^2_{Y^o|\underline{X}_{core}=\underline{x}_{core}}} \sigma^2_{\alpha_0}$,
- the G-C interaction effect $= \frac{\sigma^2_{Y^o|\underline{X}=\underline{x},C^o=c^o}}{\sigma^2_{Y^o|\underline{X}_{core}=\underline{x}_{core}}} \sigma^2_{\alpha_1}$,
- the main residual effect $= \frac{\sigma^2_{Y^o|\underline{X}=\underline{x},C^o=c^o}}{\sigma^2_{Y^o|\underline{X}_{core}=\underline{x}_{core}}} \sigma^2_{\tau_0}$, and,
- the R-C interaction effect $= \frac{\sigma^2_{Y^o|\underline{X}=\underline{x},C^o=c^o}}{\sigma^2_{Y^o|\underline{X}_{core}=\underline{x}_{core}}} \sigma^2_{\tau_1}$.

These are approximations, since the derivation ignores correlation between C and C^o, but they do provide estimates on a scale more akin to those typically seen in the literature. The maths looks messy/ complex, but the routine is simple:

- store the estimate for $\sigma^2_{Y^o|\underline{X}=\underline{x},C^o=c^o}$; the variance estimate for the residual noise from the fixed effects model adjusting for $\underline{X} = \underline{x}, C^o = c^o$,
- store the estimate for $\sigma^2_{Y^o|\underline{X}_{core}=\underline{x}_{core}}$; the variance estimate for the residual noise from the fixed effects model adjusting for $\underline{X}_{core} = \underline{x}_{core}$,
- extract the required variance component estimate from the mtg2 output, and,

- input into the above equations.

Standard errors are calculated using the SE of the variance component on the scale, for example, for the G-C interaction effect:

$$SE\Big(\frac{\sigma^2_{Y^o|\underline{X}=\underline{x},C^o=c^o}}{\sigma^2_{Y^o|\underline{X}_{core}=\underline{x}_{core}}}\sigma^2_{\alpha_1}\Big) = \frac{\sigma^2_{Y^o|\underline{X}=\underline{x},C^o=c^o}}{\sigma^2_{Y^o|\underline{X}_{core}=\underline{x}_{core}}}SE\big(\sigma^2_{\alpha_1}\big)$$

where $SE\big(\sigma^2_{\alpha_1}\big)$ is part of the output from mtg2. Again, these need to be calculated within each subgroup within our analysis and then meta-analysed to obtain presented results.

2 Supplementary Tables

2.1 Covariate traits

Table 6: Characteristics of depSympt and the covariate traits in available UK Biobank sample ($N = 119,690$). Note: all biomarkers are on their untransformed scale.

	Missing data (%)	Mean	SD	Median
depSympt	0.00	0.12	0.81	0.06
Neuroticism	15.27	3.82	3.14	3.00
Childhood trauma	1.85	1.21	1.93	0.00
Sleep	0.21	7.18	0.97	7.00
BMI	0.19	26.74	4.53	26.06
Waist circ	0.11	88.46	13.10	88.00
Smoking	32.59	0.19	0.34	0.00
WTH ratio	0.12	0.86	0.09	0.86
MET total	13.94	2425.87	2373.38	1693.00
MET walk	13.94	951.04	992.99	594.00
MET mod	13.94	837.75	1120.50	400.00
TDI	0.12	-1.78	2.79	-2.49
MET vig	13.94	637.08	1030.63	240.00
LDL	5.00	3.58	0.84	3.54
Triglycerides	4.88	1.66	0.97	1.41
Vitamin D	8.77	49.93	20.65	48.40
CRP	4.98	2.25	3.95	1.14
HDL	13.00	1.49	0.38	1.44

Table 7: Linear fixed effects model summary.

	% phenotypic variance explained	Beta	SE	p-value
Neuroticism	21.55	0.46	2.78E-03	0.000E+00
Childhood trauma	5.20	0.23	2.80E-03	0.000E+00
Sleep	0.87	-0.09	2.84E-03	7.927E-228
BMI	1.05	0.10	2.86E-03	3.162E-277
Waist circ	1.23	0.13	3.26E-03	0.000E+00
Smoking	1.54	0.13	3.59E-03	1.311E-274
WTH ratio	0.75	0.12	3.88E-03	3.283E-198
MET total	0.16	-0.04	3.07E-03	9.692E-39
MET walk	0.08	-0.03	3.07E-03	3.132E-19
MET mod	0.04	-0.02	3.09E-03	5.054E-10
TDI	0.72	0.08	2.87E-03	8.502E-190
MET vig	0.21	-0.05	3.08E-03	1.119E-48
LDL	0.00	-0.01	2.94E-03	7.361E-02
log-Triglycerides	0.40	0.06	3.01E-03	3.915E-102
log-Vitamin D	0.51	-0.07	2.99E-03	1.554E-122
log-CRP	0.22	0.05	2.92E-03	1.955E-56
log-HDL	0.38	-0.07	3.39E-03	1.913E-87

Output from a linear model for (standardised) depSympt against each standardised covariate in turn (main effect only). All models adjust for: sex, age, genotype batch and principal components 1-15.

Only LDL has a p-value $> 0.05/17$

Table 8: Comparison of the proportion of variability in depSympt explained by each covariate trait: main effects only versus fractional polynomials

	Main effects only		Fractional polynomials	
	% of variance explained	p-value	% of variance explained	p-value
Neuroticism	21.55	0.000E+00	21.61	0.000E+00
Childhood trauma	5.20	0.000E+00	5.38	0.000E+00
Sleep	0.87	7.927E-228	2.24	0.000E+00
BMI	1.05	3.162E-277	1.25	1.355E-315
Waist circ	1.23	0.000E+00	1.30	0.000E+00
Smoking	1.54	1.311E-274	1.57	3.261E-275
WTH ratio	0.75	3.283E-198	0.79	1.207E-200
MET total	0.16	9.692E-39	0.58	9.833E-126
MET walk	0.08	3.132E-19	0.28	1.965E-62
MET mod	0.04	5.054E-10	0.30	2.254E-66
TDI	0.72	8.502E-190	0.74	4.715E-187
MET vig	0.21	1.119E-48	0.68	5.247E-148
LDL†	0.00	7.361E-02	0.04	1.868E-10
log-Triglycerides	0.40	3.915E-102	0.49	2.793E-117
log-Vitamin D	0.51	1.554E-122	0.58	1.056E-133
log-CRP	0.22	1.955E-56	0.49	4.365E-117
log-HDL	0.38	1.913E-87	0.47	1.143E-104

Main effects only = a linear model for (standardised) depSympt against each standardised covariate in turn. Fractional polynomial = a linear model for (standardised) depSympt against each standardised covariate in turn allowing for transformations of the covariate traits using fractional polynomials (FPs). All models adjust for: sex, age, genotype batch and principal components 1-15. Note: the FP fixed effects models are not those used in the final interaction analysis, which adjust for more fixed effects.

† The effect of LDL on depSympt is non-significant in the main effects model ($p > 0.05/17$) but significant in the FP model

Note: The FP model presented in the main results allowed a non-linear relationship between age and depSympt. Here the FP model adjusted for age as a main effect only. Differences in % of depSympt variance explained for these FP models are due to this.

Table 9: Fractional polynomial fixed effects model summary.

	% phenotypic variance explained	Beta	SE	p-value
**Neuroticism**	21.61			**0.00E+00**
I((X + 1.6)^0.5)		0.92	1.19E-02	0.00E+00
I((X + 1.6)^3)		0.01	3.18E-04	4.89E-122
**Childhood trauma**	5.38			**0.00E+00**
log((X + 1.2))		0.28	6.95E-03	0.00E+00
I((X + 1.2)^2)		0.01	8.75E-04	1.08E-45
**Sleep**	2.24			**0.00E+00**
I(((X + 7.5)/10)^2)		-2.10	4.03E-02	0.00E+00
I(((X + 7.5)/10)^2*log(((X + 7.5)/10)))		6.52	1.46E-01	0.00E+00
**BMI**	1.25			**1.35E-315**
log(((X + 3.3)/10))		-1.65	8.53E-02	6.93E-83
I(((X + 3.3)/10)^0.5)		6.84	2.96E-01	1.65E-117
**Waist circumference**	1.30			**0.00E+00**
I(((X + 3.6)/10)^1)		-0.15	1.59E-01	3.38E-01
I(((X + 3.6)/10)^2)		1.80	1.99E-01	1.25E-19
**Smoking**	1.57			**3.26E-275**
I((X + 0.6)^-2)		-0.00	3.92E-06	3.63E-06
I((X + 0.6)^1)		0.11	5.07E-03	5.87E-106
**Waist-to-hip ratio**	0.79			**1.21E-200**
I(((X + 4.7)/10)^3)		1.07	5.35E-02	1.30E-88
I(((X + 4.7)/10)^3*log(((X + 4.7)/10)))		-1.71	1.40E-01	4.22E-34
**MET tot**	0.58			**9.83E-126**
I((X + 1.1)^-0.5)		0.15	7.02E-03	1.28E-94
I((X + 1.1)^1)		0.02	4.35E-03	5.19E-08
**MET walk**	0.28			**1.96E-62**
I((X + 1)^-2)		0.00	3.81E-05	2.06E-20
log((X + 1))		-0.02	3.69E-03	6.21E-10
**MET mod**	0.30			**2.25E-66**
I((X + 0.8)^-0.5)		0.06	4.30E-03	4.60E-48
I((X + 0.8)^0.5)		0.07	1.11E-02	4.80E-09
**TDI**	0.74			**4.71E-187**
I((X + 1.7)^1)		0.03	1.03E-02	7.98E-04
I((X + 1.7)^2)		0.01	2.25E-03	4.34E-07
**MET vig**	0.68			**5.25E-148**
log((X + 0.7))		-0.09	4.12E-03	7.11E-108
I((X + 0.7)^1)		0.05	5.18E-03	1.45E-19
**LDL**	0.04			**1.87E-10**

Continued on next page

Table 9 – continued from previous page

	% phenotypic variance explained	Beta	SE	p-value
I(((X + 3.3)/10)^0.5)		-0.76	1.13E-01	1.20E-11
I(((X + 3.3)/10)^0.5*log(((X + 3.3)/10)))		0.84	1.33E-01	2.75E-10
**log-Triglycerides**	0.49			**2.79E-117**
I((X + 1.5)^-2)		0.00	4.59E-04	4.57E-04
log((X + 1.5))		0.12	5.16E-03	2.34E-121
**log-Vitamin D**	0.58			**1.06E-133**
I((X + 2)^1)		-0.21	1.53E-02	2.45E-41
I((X + 2)^1*log((X + 2)))		0.08	8.72E-03	1.62E-19
**log-C-Reactive Protein**	0.49			**4.37E-117**
I((X + 0.6)^0.5)		0.34	1.94E-02	3.72E-68
I((X + 0.6)^1)		-0.07	7.52E-03	2.86E-23
**log-HDL**	0.47			**1.14E-104**
I(((X + 3.3)/10)^1)		-0.62	3.43E-02	1.84E-72
I(((X + 3.3)/10)^1*log(((X + 3.3)/10)))		1.52	1.50E-01	4.26E-24

Output from a linear model for depSympt against each covariate in turn (fractional polynomials used).
All models adjust for: sex, age, genotype batch and principal components 1-15.
Fractional polynomial transformations provided. X = standardised covariate trait.
These transformations correspond to the FP model summaries provided in Table 8 above.
Two p-value types presented: 1. Wald p-values corresponding to each beta estimate, and,
2. global LRT p-values (bold font) testing for inclusion of all selected FP transformations.

2.2 Fractional polynomial interaction models

Table 10: Likelihood ratio test statistics and p-values comparing the full versus the null multivariate reaction norm models.

Covariate	Test statistic (meta)	p-value (meta)
**Neuroticism**	658.69	5.06E-139
**Childhood trauma**	283.66	2.59E-58
**Sleep**	204.63	1.97E-41
**BMI**	93.26	6.36E-18
**Waist circ**	83.70	6.15E-16
**Smoking**	56.34	2.49E-10
**WTH ratio**	50.10	4.49E-09
**MET total**	41.90	1.92E-07
**MET walk**	32.83	1.13E-05
**MET mod**	23.78	5.73E-04
**TDI**	20.84	1.96E-03
MET vig	18.09	6.00E-03
LDL	12.57	5.04E-02
log-Triglycerides	11.32	7.90E-02
log-Vitamin D	5.84	4.42E-01
log-CRP	4.69	5.85E-01
log-HDL	3.52	7.42E-01

full model = both G-C and R-C interactions.
null model = no interactions.
Results are ordered by p-value.
Significance is set at $\alpha = 0.05/17 \approx 0.003$.
Significance results highlighted in bold.

Table 11: LRT statistics and p-values: all covariates, all subgroups

	Test statistic				p-value			
	Subgroup 1	Subgroup 2	Subgroup 3	Meta	Subgroup 1	Subgroup 2	Subgroup 3	Meta
Neu	243.85	220.81	247.27	658.69	8.46E-50	6.99E-45	1.57E-50	5.06E-139
Childhood trauma	125.13	123.41	78.93	283.66	1.36E-24	3.13E-24	5.94E-15	2.59E-58
Sleep	73.51	75.32	96.36	204.63	7.77E-14	3.29E-14	1.44E-18	1.97E-41
BMI	44.99	32.50	48.56	93.26	4.71E-08	1.31E-05	9.11E-09	6.36E-18
Waist circ	40.47	31.14	43.93	83.70	3.68E-07	2.38E-05	7.64E-08	6.15E-16
Smoking	17.24	57.04	6.34	56.34	8.45E-03	1.79E-10	3.86E-01	2.49E-10
WTH ratio	21.09	17.93	37.92	50.10	1.77E-03	6.41E-03	1.17E-06	4.49E-09
MET total	12.20	18.42	36.11	41.90	5.76E-02	5.27E-03	2.62E-06	1.92E-07
MET walk	9.17	13.73	32.38	32.83	1.64E-01	3.28E-02	1.38E-05	1.13E-05
MET mod	9.69	11.21	23.65	23.78	1.38E-01	8.21E-02	6.05E-04	5.73E-04
TDI	19.34	9.65	12.16	20.84	3.62E-03	1.40E-01	5.85E-02	1.96E-03
MET vig	7.03	14.65	15.62	18.09	3.18E-01	2.32E-02	1.60E-02	6.00E-03
LDL	18.37	5.81	3.21	12.57	5.37E-03	4.44E-01	7.82E-01	5.04E-02
log-Tri	16.73	2.76	6.19	11.32	1.03E-02	8.39E-01	4.02E-01	7.90E-02
log-Vit D	9.07	5.07	4.61	5.84	1.70E-01	5.35E-01	5.94E-01	4.42E-01
log-CRP	5.50	6.09	5.49	4.69	4.82E-01	4.13E-01	4.83E-01	5.85E-01
log-HDL	4.33	7.11	2.44	3.52	6.33E-01	3.11E-01	8.75E-01	7.42E-01

Meta p-values and test statistics were calculated using Fishers method. See SM Section 1.4 for details.

Table 12: Likelihood ratio test statistics and p-values when comparing models with and without interactions, using untransformed depSympt and RINT depSympt (sensitivity analysis).

	Untransformed depSympt		RINT depSympt	
	Test statistic	p-value	Test statistic	p-value
**Neuroticism**	658.69	5.06E-139	740.15	1.31E-156
**Childhood trauma**	283.66	2.59E-58	343.97	3.03E-71
**Sleep**	204.63	1.97E-41	228.33	1.74E-46
**BMI**	93.26	6.36E-18	123.71	2.71E-24
**Waist circ**	83.70	6.15E-16	101.84	1.03E-19
**Smoking**	56.34	2.49E-10	66.63	2.00E-12
**WTH ratio**	50.10	4.49E-09	61.82	1.92E-11
**MET total**	41.90	1.92E-07	36.04	2.71E-06
**MET walk**	32.83	1.13E-05	33.57	8.16E-06
**MET mod**	23.78	5.73E-04	21.13	1.74E-03
**TDI**	20.84	1.96E-03	27.35	1.25E-04
MET vig	18.09	6.00E-03	14.88	2.12E-02
LDL	12.57	5.04E-02	10.41	1.08E-01
log-Tri	11.32	7.90E-02	13.69	3.33E-02
log-Vitamin D	5.84	4.42E-01	7.61	2.68E-01
log-CRP	4.69	5.85E-01	4.61	5.95E-01
log-HDL	3.52	7.42E-01	2.64	8.53E-01

RINT = rank-based inverse normal transformation. Covariates highlighted in bold had p-values $< 0.05/17$ when using untransformed depSympt as the outcome. Covariates above the line had p-values $< 0.05/17$ when using RINT depSympt as the outcome.

Table 13: Percentage of variability in depSympt attributable to: 1. the fixed effects, 2. the G-C interaction and 3. the R-C interaction, for each covariate trait.

Covariate	Fixed effect	G-C interaction		R-C interaction	
		Estimate	95% CI	Estimate	95% CI
Neuroticism	15.15	-0.15	[-0.76, 0.46]	2.58	[1.86, 3.30]
Childhood trauma	1.51	0.59	[-0.14, 1.32]	2.98	[2.18, 3.77]
Sleep	1.17	1.22	[0.54, 1.89]	2.52	[1.78, 3.27]
BMI	0.37	-0.23	[-0.86, 0.41]	1.39	[0.68, 2.09]
Waist circ	0.40	-0.15	[-0.78, 0.48]	1.48	[0.78, 2.19]
Smoking	0.18	0.47	[-0.52, 1.46]	1.57	[0.51, 2.63]
WTH ratio	0.23	-0.33	[-0.95, 0.29]	1.03	[0.34, 1.73]
MET tot	0.32	0.23	[-0.42, 0.87]	0.53	[-0.17, 1.24]
MET walk	0.16	0.10	[-0.55, 0.74]	1.18	[0.45, 1.92]
MET mod	0.15	-0.26	[-0.87, 0.35]	-0.08	[-0.78, 0.61]
TDI	0.05	-0.19	[-0.81, 0.42]	1.67	[0.97, 2.38]
MET vig	0.35	0.51	[-0.16, 1.17]	0.26	[-0.44, 0.95]
LDL	0.01	-0.23	[-0.89, 0.44]	0.89	[0.14, 1.65]
log-Tri	0.08	0.45	[-0.22, 1.12]	0.38	[-0.37, 1.13]
log-Vitamin D	0.10	-0.17	[-0.83, 0.49]	1.11	[0.35, 1.87]
log-CRP	0.00	0.85	[0.19, 1.51]	-0.63	[-1.35, 0.10]
log-HDL	0.00	-0.62	[-1.33, 0.08]	1.05	[0.26, 1.84]

FPs used in the fixed effects model. Table is ordered by p-value.

Please see Table 10 for p-values.

Table 14: Percentage of variability in *residual* depSympt attributable to genotype-covariate (G-C) and residual-covariate (R-C) interactions.

	G-C interaction		R-C interaction	
Covariate	Estimate	95% CI	Estimate	95% CI
Neuroticism	-0.21	[-1.06, 0.64]	3.61	[2.60, 4.61]
Childhood trauma	0.68	[-0.16, 1.52]	3.43	[2.51, 4.35]
Sleep	1.41	[0.63, 2.19]	2.92	[2.05, 3.78]
BMI	-0.26	[-1.00, 0.47]	1.60	[0.79, 2.42]
Waist circ	-0.17	[-0.90, 0.56]	1.72	[0.90, 2.53]
Smoking	0.55	[-0.61, 1.71]	1.84	[0.59, 3.08]
WTH ratio	-0.38	[-1.10, 0.33]	1.19	[0.39, 1.99]
MET total	0.26	[-0.49, 1.01]	0.61	[-0.20, 1.43]
MET walk	0.11	[-0.63, 0.85]	1.37	[0.51, 2.22]
MET mod	-0.30	[-1.00, 0.40]	-0.10	[-0.89, 0.70]
TDI	-0.23	[-0.94, 0.49]	1.93	[1.12, 2.75]
MET vig	0.58	[-0.18, 1.35]	0.30	[-0.51, 1.10]
LDL	-0.26	[-1.03, 0.50]	1.03	[0.16, 1.90]
log-Tri	0.52	[-0.26, 1.30]	0.44	[-0.43, 1.31]
log-Vitamin D	-0.20	[-0.96, 0.57]	1.28	[0.41, 2.16]
log-CRP	0.99	[0.22, 1.75]	-0.72	[-1.56, 0.11]
log-HDL	-0.72	[-1.54, 0.10]	1.22	[0.30, 2.13]

FPs used in the fixed effects model. Table is ordered by p-value.
Please see Table 10 for p-values.

Table 15: Percentage of variability in residual depSympt[a] attributable to polygenic and residual variation[b].

Percentage of the variability in residual depSympt[a] attributable to the...

Covariate trait (C)	Main polygenic variance component Estimate	Main polygenic variance component 95% CI	G-C interaction variance component Estimate	G-C interaction variance component 95% CI	Main residual variance component Estimate	Main residual variance component 95% CI	R-C interaction variance component Estimate	R-C interaction variance component 95% CI
Neuroticism	2.6	[1.8, 3.4]	-0.2	[-1.1, 0.6]	94.0	[92.9, 95.1]	3.6	[2.6, 4.6]
Childhood trauma	6.3	[5.5, 7.0]	0.7	[-0.2, 1.5]	89.7	[88.8, 90.6]	3.4	[2.5, 4.4]
Sleep	6.4	[5.7, 7.1]	1.4	[0.6, 2.2]	89.3	[88.3, 90.2]	2.9	[2.1, 3.8]
BMI	6.4	[5.7, 7.2]	-0.3	[-1.0, 0.5]	92.2	[91.3, 93.1]	1.6	[0.8, 2.4]
Waist circumference	6.3	[5.6, 7.1]	-0.2	[-0.9, 0.6]	92.1	[91.2, 93.1]	1.7	[0.9, 2.5]
Smoking	5.5	[4.4, 6.5]	0.5	[-0.6, 1.7]	92.1	[90.8, 93.3]	1.8	[0.6, 3.1]
WTH ratio	6.2	[5.5, 7.0]	-0.4	[-1.1, 0.3]	92.9	[92.0, 93.9]	1.2	[0.4, 2.0]
MET total	6.3	[5.6, 7.1]	0.3	[-0.5, 1.0]	92.8	[91.8, 93.7]	0.6	[-0.2, 1.4]
MET walk	6.4	[5.6, 7.1]	0.1	[-0.6, 0.8]	92.1	[91.1, 93.1]	1.4	[0.5, 2.2]
MET mod	6.4	[5.7, 7.2]	-0.3	[-1.0, 0.4]	94.0	[93.0, 94.9]	-0.1	[-0.9, 0.7]
TDI	6.4	[5.6, 7.1]	-0.2	[-0.9, 0.5]	91.9	[91.0, 92.8]	1.9	[1.1, 2.7]
MET vig	6.3	[5.6, 7.0]			93.7	[93.0, 94.4]		
LDL	6.6	[5.8, 7.4]			93.4	[92.6, 94.2]		
Triglycerides	6.6	[5.8, 7.4]			93.4	[92.6, 94.2]		
Vitamin D	6.6	[5.8, 7.4]			93.4	[92.6, 94.2]		
CRP	6.6	[5.8, 7.4]			93.4	[92.6, 94.2]		
HDL	6.2	[5.3, 7.1]			93.8	[92.9, 94.7]		

[a] Residualised depSympt is depSympt after adjustment for the fixed effects outlined in Supplementary Methods section 1.2 (Phenotype adjustment) and Table 5. These fixed effects vary by covariate trait- residual depSympt is not the same across covariate traits.

Note: the interaction percentages do not match those presented in the main text, which have been re-scaled as described in Supplementary Methods section 1.4.4.

[b] Main polygenic variance component = homogeneous additive genetic variation (invariant to C). G-C (genotype-covariate) interaction variance component = additive genetic variation that changes with C. Main residual variance component = homogeneous residual variation. R-C (residual-covariate) interaction variance component = residual variation that changes with C.

Covariate traits below the line demonstrated no evidence of modulating the polygenic or residual effects on depSympt. Therefore estimates from the 'null' model (without interactions) are presented.

Table 16: Percentage of variability in depSympt[a] attributable to the fixed effects[b] and to polygenic and residual variation[c].

		**Percentage of the variability in depSympt[a] attributable to the...**							
		Main polygenic variance component		G-C interaction variance component		Main residual variance component		R-C interaction variance component	
Covariate trait (C)	Fixed effects[b]	Estimate	95% CI	Estimate	95% CI	Estimate	95% CI	Estimate	95% CI
Neuroticism	28.5	1.9	[1.3, 2.4]	-0.2	[-0.8, 0.5]	67.2	[66.5, 68.0]	2.6	[1.9, 3.3]
Childhood trauma	13.3	5.4	[4.8, 6.1]	0.6	[-0.1, 1.3]	77.7	[76.9, 78.5]	3.0	[2.2, 3.8]
Sleep	13.4	5.5	[4.9, 6.2]	1.2	[0.5, 1.9]	77.3	[76.5, 78.1]	2.5	[1.8, 3.3]
BMI	13.4	5.6	[4.9, 6.2]	-0.2	[-0.9, 0.4]	79.9	[79.0, 80.7]	1.4	[0.7, 2.1]
Waist circumference	13.5	5.5	[4.8, 6.1]	-0.1	[-0.8, 0.5]	79.7	[78.9, 80.6]	1.5	[0.8, 2.2]
Smoking	14.4	4.7	[3.7, 5.6]	0.5	[-0.5, 1.5]	78.8	[77.7, 79.9]	1.6	[0.5, 2.6]
WTH ratio	13.3	5.4	[4.8, 6.1]	-0.3	[-1.0, 0.3]	80.6	[79.8, 81.4]	1.0	[0.3, 1.7]
MET total	13.4	5.5	[4.8, 6.1]	0.2	[-0.4, 0.9]	80.3	[79.5, 81.1]	0.5	[-0.2, 1.2]
MET walk	13.3	5.5	[4.9, 6.2]	0.1	[-0.5, 0.7]	79.9	[79.1, 80.7]	1.2	[0.4, 1.9]
MET mod	13.3	5.6	[4.9, 6.2]	-0.3	[-0.9, 0.4]	81.5	[80.7, 82.4]	-0.1	[-0.8, 0.6]
TDI	13.4	5.5	[4.9, 6.2]	-0.2	[-0.8, 0.4]	79.6	[78.8, 80.4]	1.7	[1.0, 2.4]
MET vig	13.5	5.5	[4.8, 6.1]			81.0	[70.4, 81.7]		
LDL	13.6	5.7	[5.0, 6.4]			80.7	[80.0, 81.4]		
Triglycerides	13.6	5.7	[5.0, 6.4]			80.7	[80.0, 81.4]		
Vitamin D	13.6	5.7	[5.0, 6.4]			80.7	[80.0, 81.4]		
CRP	13.6	5.7	[5.0, 6.4]			80.7	[80.0, 81.4]		
HDL	13.6	5.4	[4.6, 6.1]			81.0	[80.3, 81.8]		

[a] depSympt is conditional on sex, age, genotype batch and principal components 1 to 15, with presented variance components re-scaled as described in Supplementary Methods section 1.4.4. Note: the interaction percentages now match those presented in the main text.

[b] Fixed effects are those presented in Supplementary section 1.2 (Phenotype adjustment)/ Table 5 *except* those listed in a.

Polygenic and residual variance components still *control* for all of the fixed effects in Supplementary section 1.2, but are re-scaled so that depSympt is only conditional on the variables listed in a. This is why the genetic variance components do not sum to the GREML SNP-based heritability estimate for depsympt provided in the paper (8.5%), which does not control for the additional fixed effects.

[c] Main polygenic variance component = homogeneous additive genetic variation (invariant to C). G-C (genotype-covariate) interaction variance component = additive genetic variation that changes with C. Main residual variance component = homogeneous residual variation. R-C (residual-covariate) interaction variance component = residual variation that changes with C.

Covariate traits below the line demonstrated no evidence of modulating the polygenic or residual effects on depSympt. Therefore estimates from the 'null' model (without interactions) are presented.

2.3 Notes on Tables 15 and 16

Table 15 presents the percentage of variability in depSympt attributable to the four variance component types explored within this study (homogeneous/ main polygenic, G-C interaction, homogeneous/ main residual and R-C interaction), given the full fixed effects models presented in Supplementary Methods section 1.2, for the 17 covariate traits.

Table 16 re-scales the variance component estimates in Table 15 such that depSympt is only conditional on a subset of the fixed effects (age, sex, genotype batch and principal components 1 to 15) because it is common in genetic studies to present SNP-heritability estimates adjusted for age, sex, genotype batch and principal components 1 to 15. However, we note that we have not re-run models excluding all the additional fixed effects. Rather, these variables are still controlled for, but we have re-scaled the variance component estimates to be on a scale more familiar with readers. Without re-scaling, the percentage of variability explained by the interaction variance components is greater than or equal to the percentages with re-scaling. Re-scaling therefore ensures that readers do not assume that the interactions have a larger effect than they do.

These tables show there is an interesting relationship between neuroticism and depSympt. Firstly, they show that including neuroticism in the model leads to a large increase in the proportion of variability in depSympt explained by the fixed effects. Table 16 shows that the fixed effects, when neuroticism is the covariate trait (and so adjusted for in the fixed effects model), explain 28.5% of the variability in (re-scaled) depSympt. The next highest percentage of depSympt explained is when smoking is the covariate trait at 14.4%.

Secondly, that there is potentially a high degree of genetic correlation between neuroticism and depSympt. The SNP-based heritability for depSympt (only adjusting for age, sex, genotype batch and principal components 1 to 15) was estimated to be 8.5% via GREML. Adjusting for additional fixed effects, provided they have some effect on expected depSympt, will reduce the amount of residual variation to be decomposed into variance components. A reduction in the contribution of the total polygenic variance component suggests there is some degree of additive genetic overlap between depSympt and the additional fixed effects. The larger the reduction, the greater the degree of genetic correlation. For most of the covariate traits considered, the percentage of variability in depSympt explained the total polygenic variance component (main polygenic + G-C interaction variance components) lie between 5% and 7%, suggesting some genetic overlap between the fixed effects and depSympt- although dissecting which fixed effects are genetically correlated, and to the extent, cannot be unpicked here. The percentage of variability in depSympt explained the total polygenic variance component when neuroticism is the covariate trait is 1.7% (main polygenic + G-C interaction variance components)- a much larger reduction than is observed for any other covariate trait. The relationship between depSympt and neuroticism is not further explored here, however it is interesting that a variable developed to measure current depressive symptoms is highly

related to neuroticism (measured 5 to 10 years prior to the depressive symptoms).

Finally, the tables highlight that the fixed effects model can remove genetic variation shared between the fixed effects and the outcome trait. The G-C interaction effects presented here are those occurring via the residual polygenic component after fixed effect adjustment. That is, this work considers the question: after considering a range of available fixed effects (such as BMI, activity level, trauma and stressful life events) is there any evidence that the polygenic effects on depSympt are modulated by a covariate trait? This is a similar approach to that taken in Ni et al. [2019] and Xuan et al. [2020].

3 Supplementary Figures

3.1 Covariate traits distributions

3.1.1 Covariates in Analysis group 1

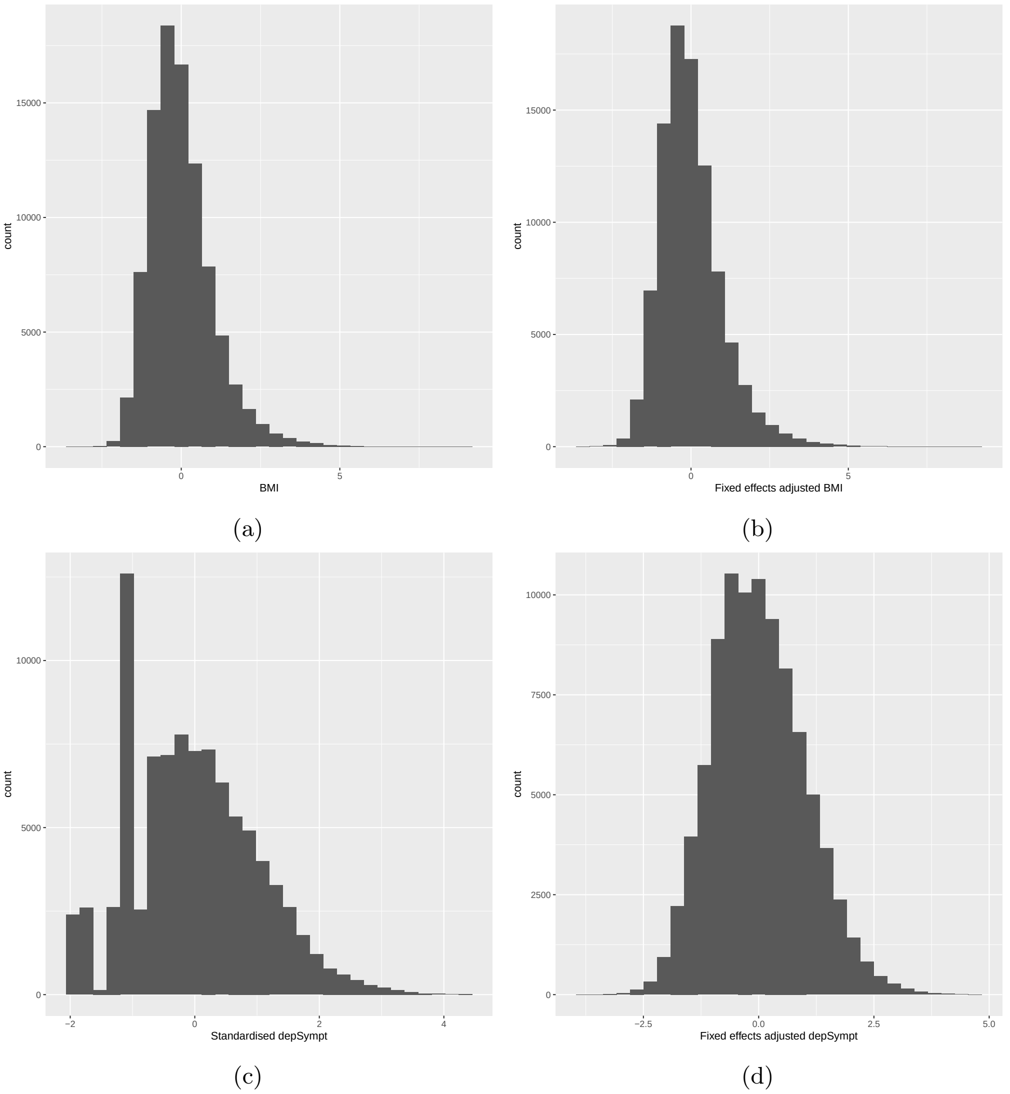

Figure 3: Histogram of standardised: (a) BMI, (b) BMI post fixed effects adjustment, (c) depSympt (Analysis group 1) and (d) depSympt post fixed effects adjustment (for use in interaction analysis with BMI), in the available UK Biobank study population

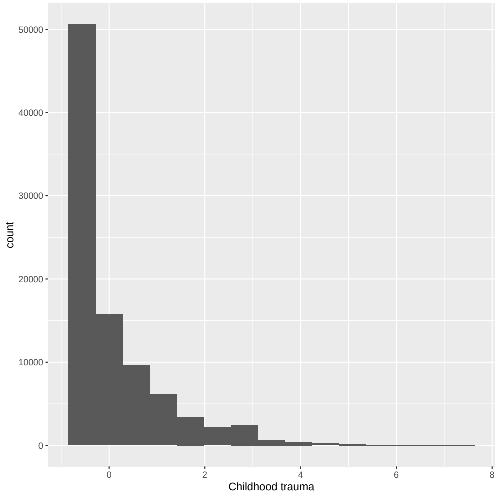

(a)

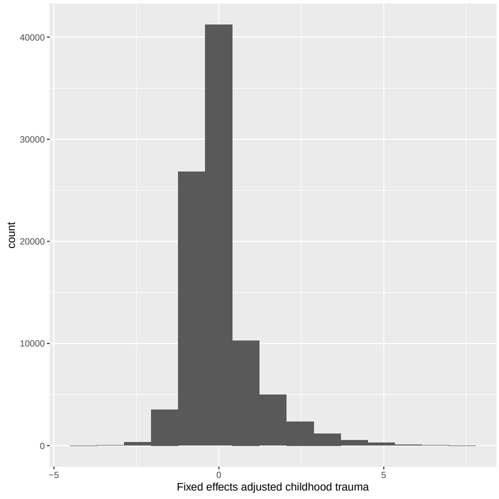

(b)

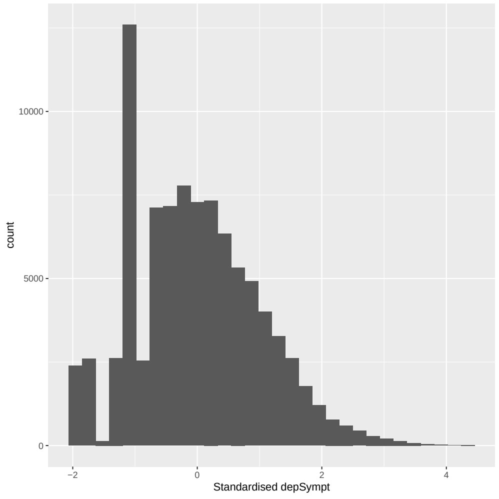

(c)

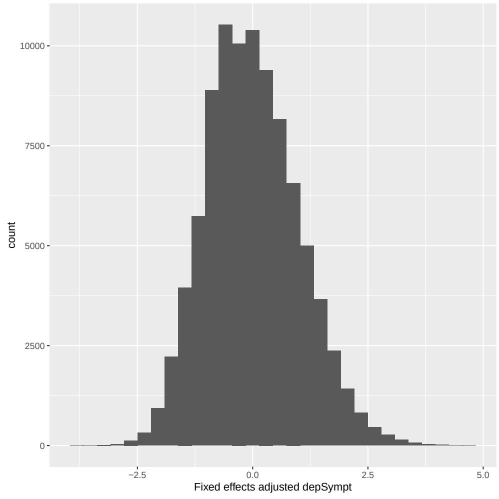

(d)

Figure 4: Histogram of standardised: (a) childhood trauma summary variable, (b) childhood trauma summary variable post fixed effects adjustment, (c) depSympt (Analysis group 1) and (d) depSympt post fixed effects adjustment (for use in interaction analysis with childhood trauma), in the available UK Biobank study population.

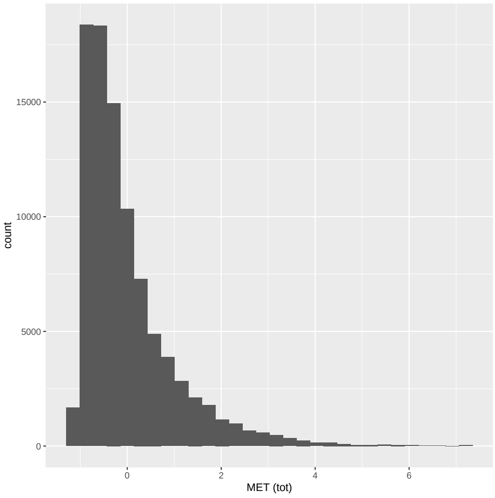

(a)

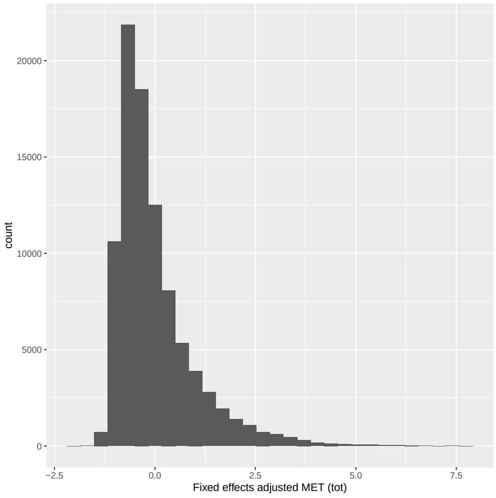

(b)

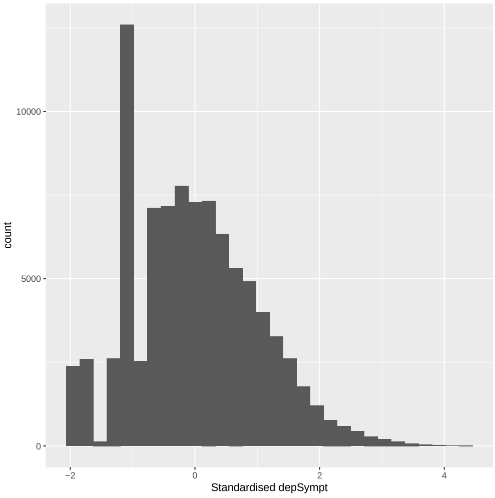

(c)

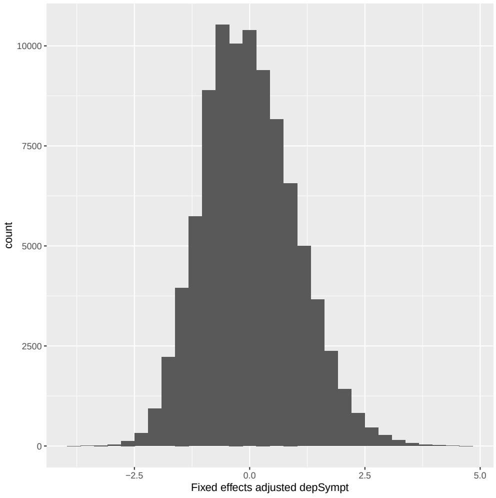

(d)

Figure 5: Histogram of standardised: (a) MET total, (b) MET total post fixed effects adjustment, (c) depSympt (Analysis group 1) and (d) depSympt post fixed effects adjustment (for use in interaction analysis with MET total), in the available UK Biobank study population.

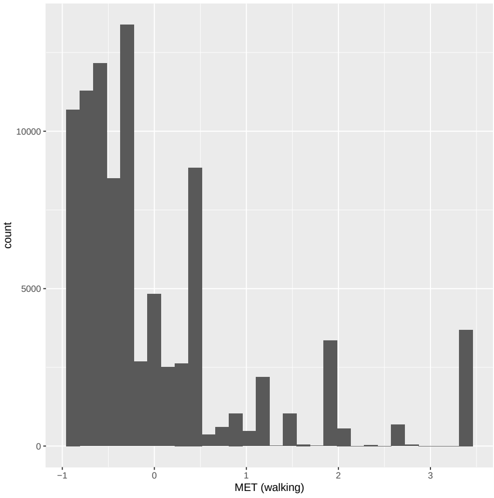

(a)

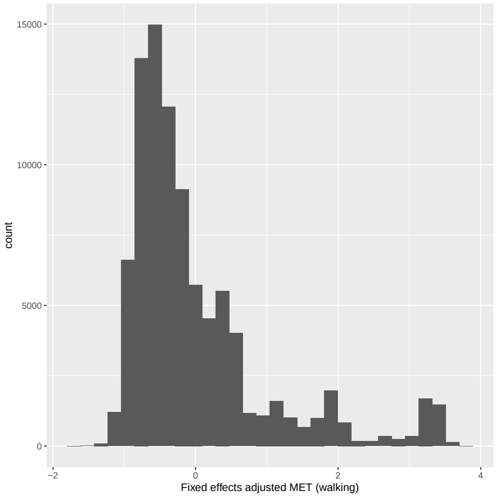

(b)

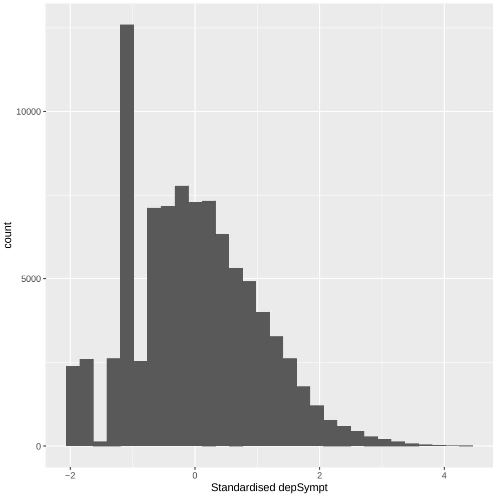

(c)

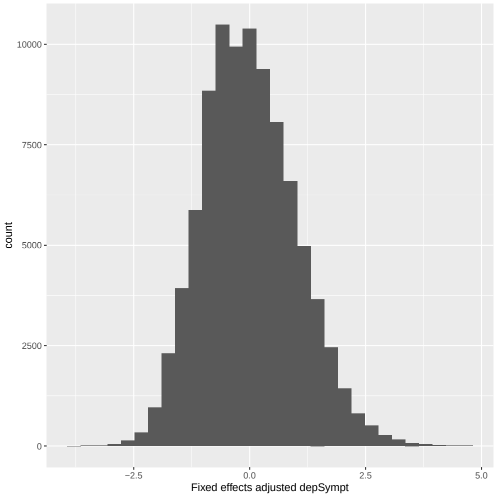

(d)

Figure 6: Histogram of standardised: (a) MET walk, (b) MET walk post fixed effects adjustment, (c) depSympt (Analysis group 1) and (d) depSympt post fixed effects adjustment (for use in interaction analysis with MET walk), in the available UK Biobank study population.

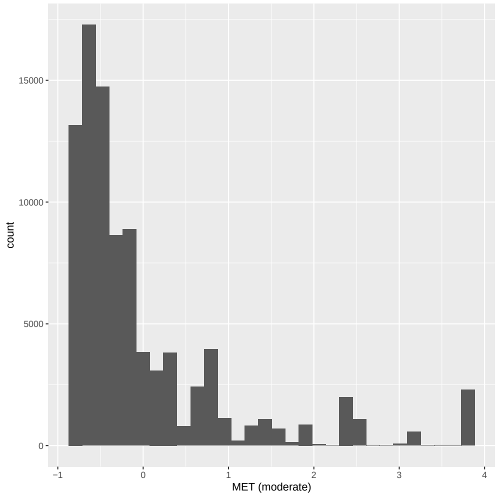

(a)

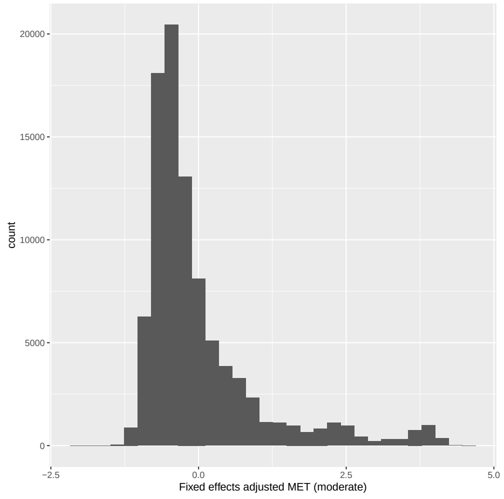

(b)

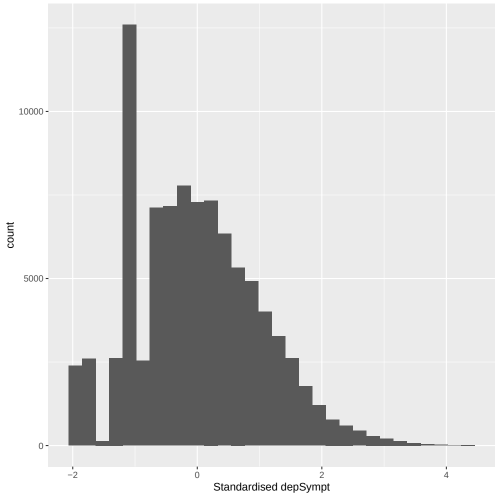

(c)

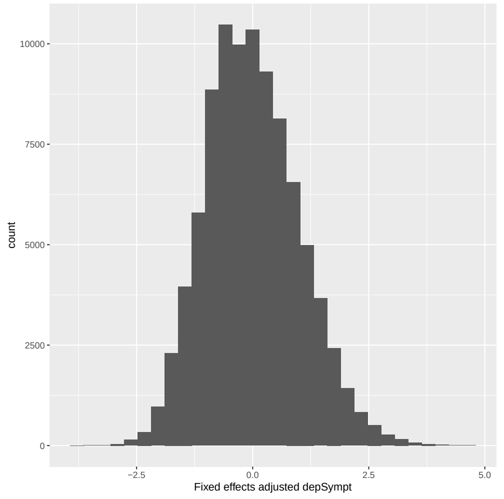

(d)

Figure 7: Histogram of standardised: (a) MET moderate, (b) MET moderate post fixed effects adjustment, (c) depSympt (Analysis group 1) and (d) depSympt post fixed effects adjustment (for use in interaction analysis with MET moderate), in the available UK Biobank study population.

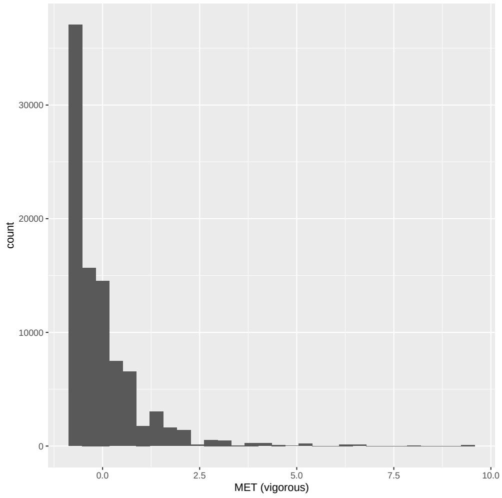

(a)

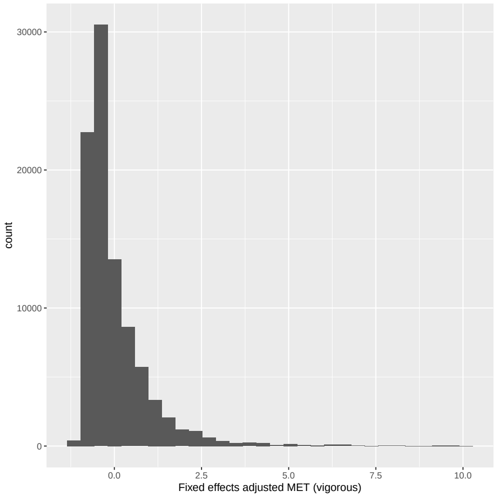

(b)

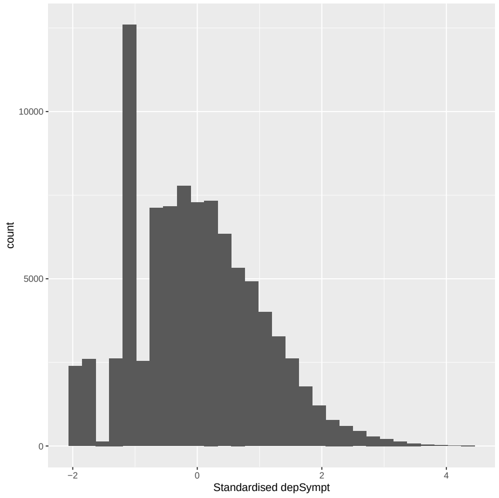

(c)

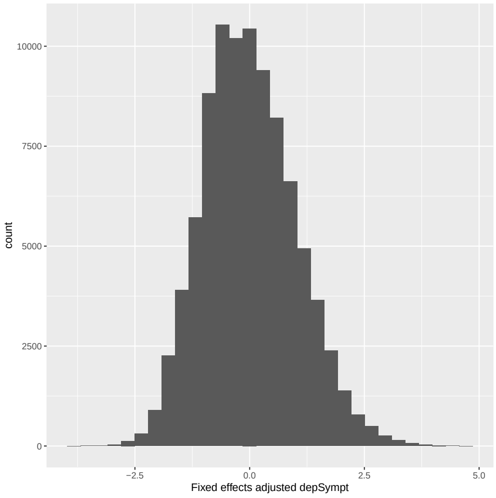

(d)

Figure 8: Histogram of standardised: (a) MET vigorous, (b) MET vigorous post fixed effects adjustment, (c) depSympt (Analysis group 1) and (d) depSympt post fixed effects adjustment (for use in interaction analysis with MET vigorous), in the available UK Biobank study population.

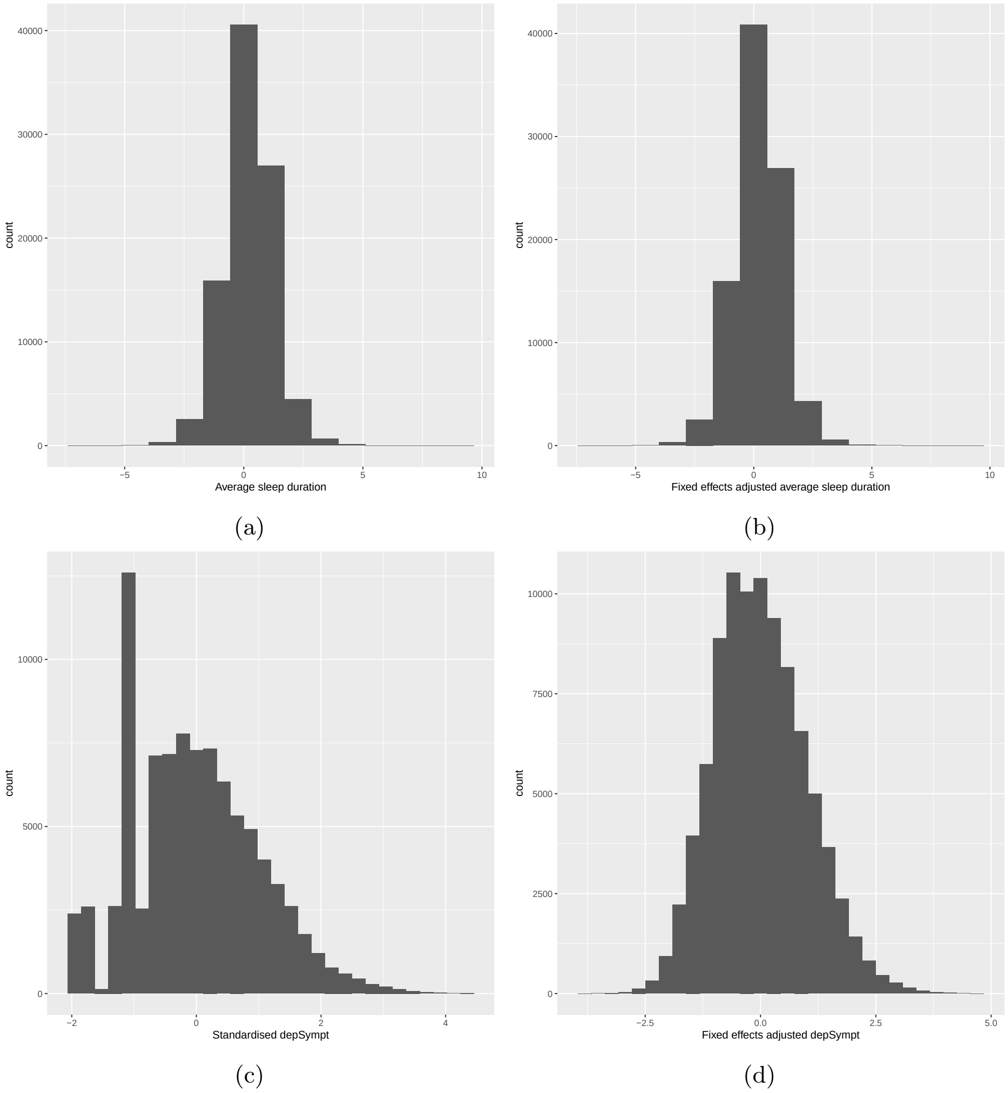

Figure 9: Histogram of standardised: (a) average sleep duration, (b) average sleep duration post fixed effects adjustment, (c) depSympt (Analysis group 1) and (d) depSympt post fixed effects adjustment (for use in interaction analysis with average sleep duration), in the available UK Biobank study population.

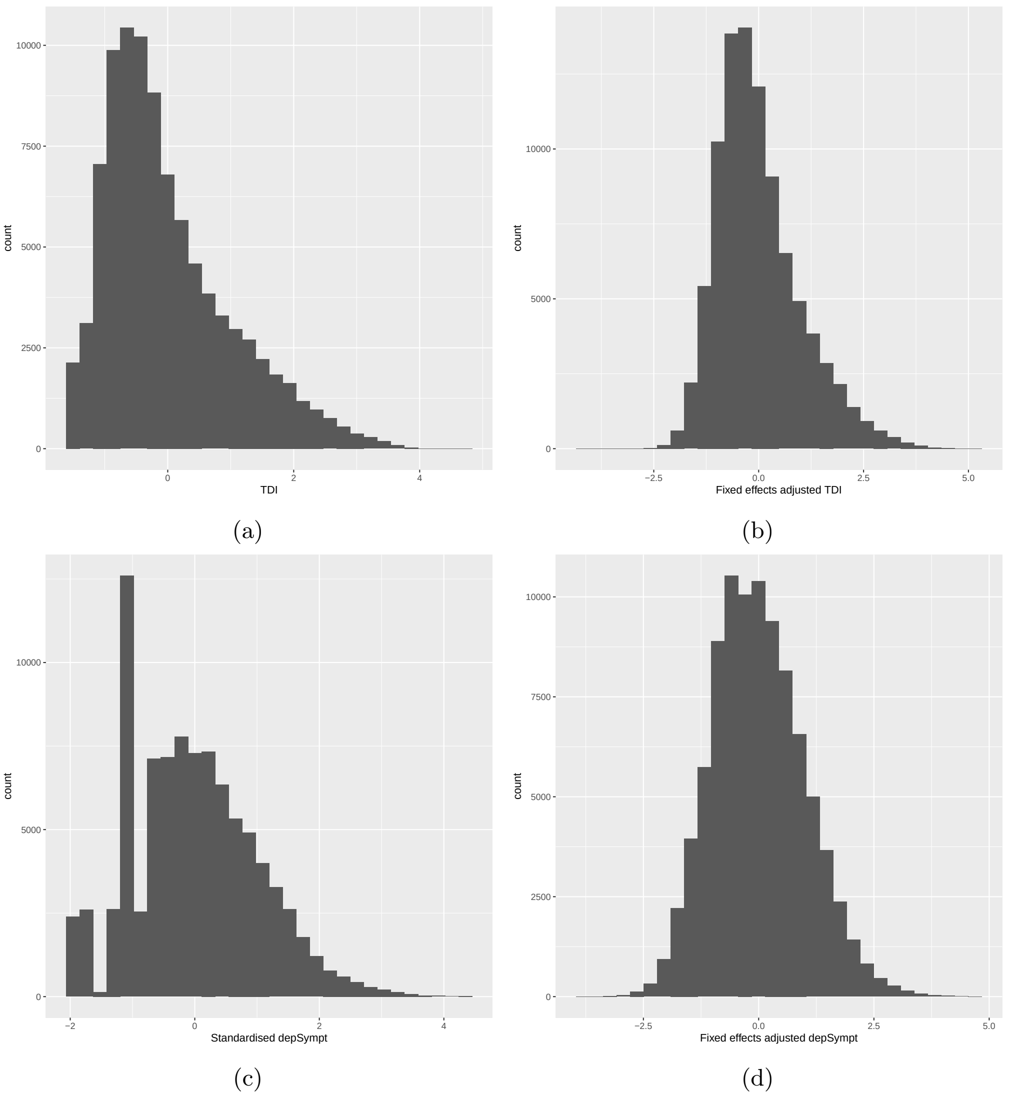

Figure 10: Histogram of standardised: (a) TDI, (b) TDI post fixed effects adjustment, (c) depSympt (Analysis group 1) and (d) depSympt post fixed effects adjustment (for use in interaction analysis with TDI), in the available UK Biobank study population.

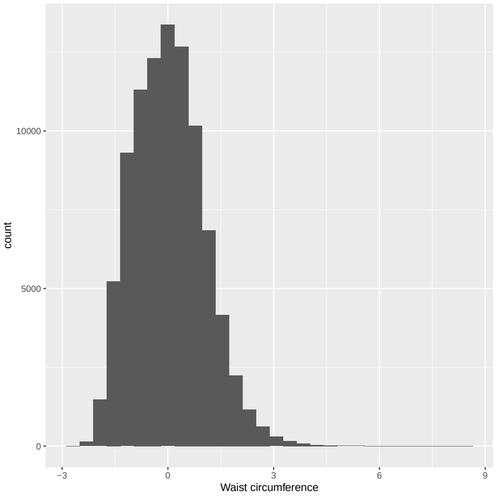

(a)

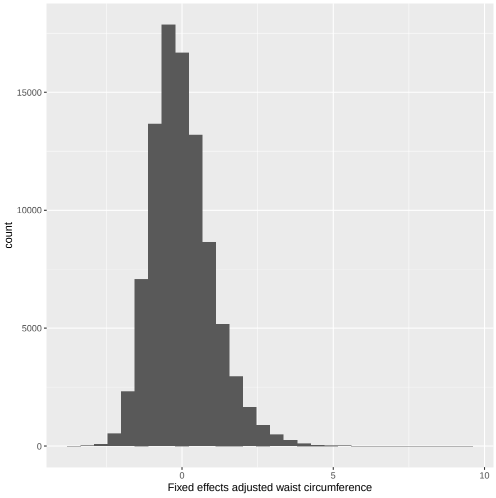

(b)

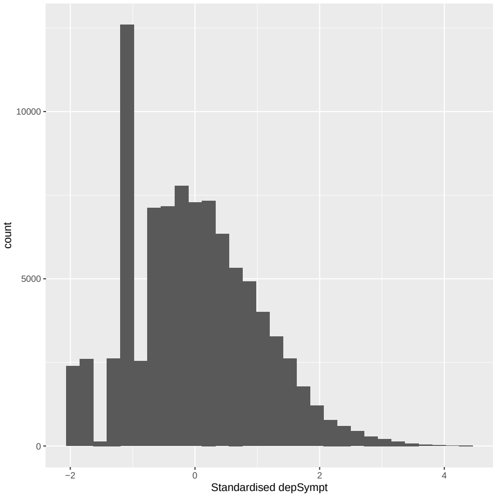

(c)

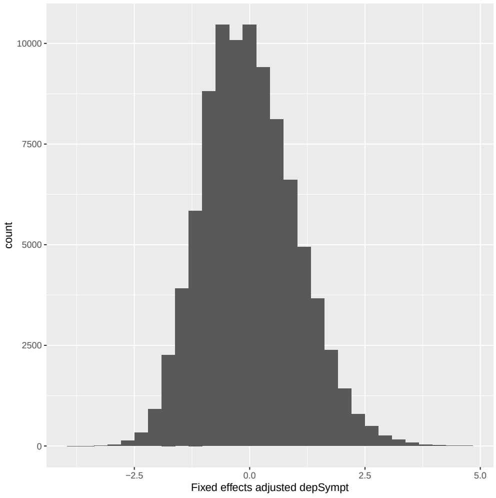

(d)

Figure 11: Histogram of standardised: (a) waist circumference, (b) waist circumference post fixed effects adjustment, (c) depSympt (Analysis group 1) and (d) depSympt post fixed effects adjustment (for use in interaction analysis with waist circumference), in the available UK Biobank study population.

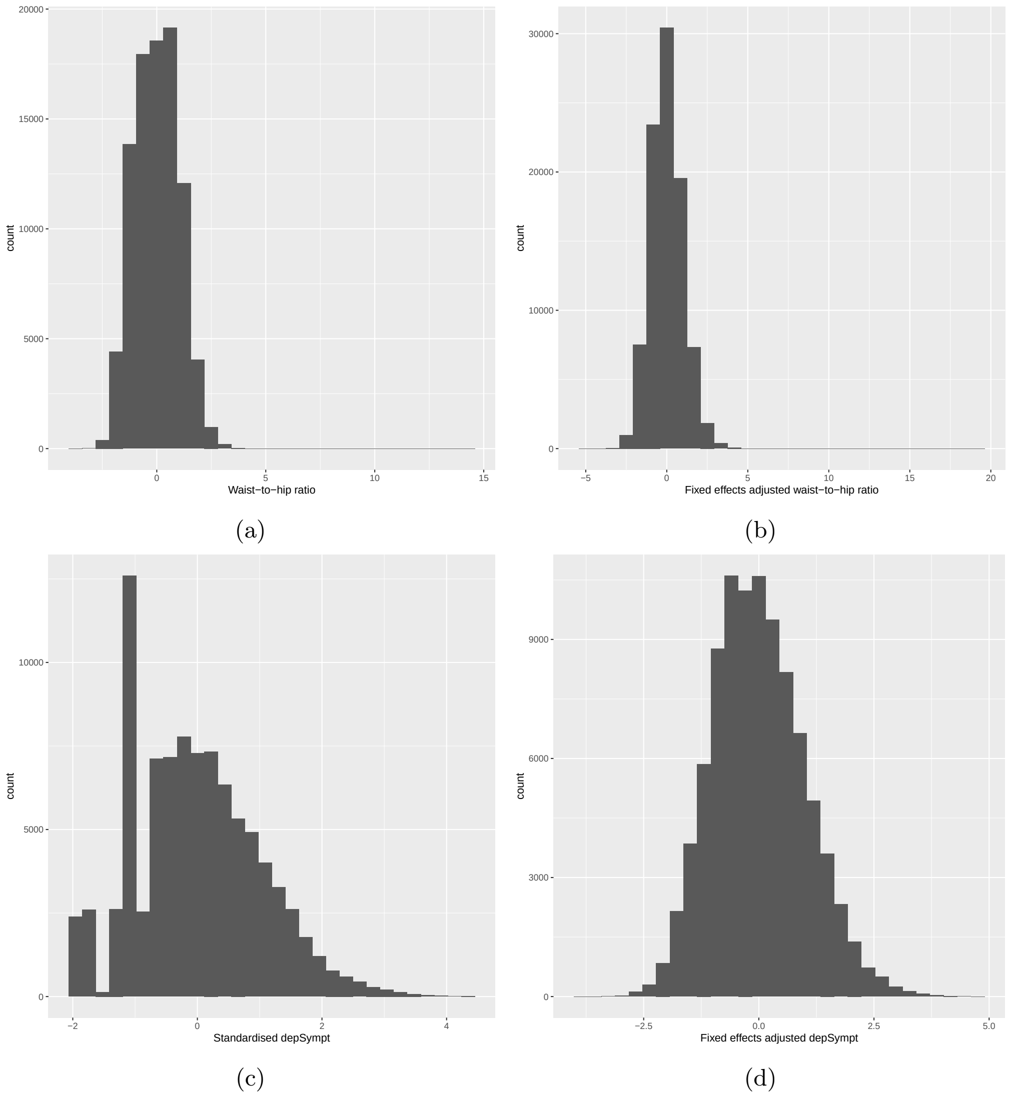

Figure 12: Histogram of standardised: (a) waist to hip ratio, (b) waist to hip ratio post fixed effects adjustment, (c) depSympt (Analysis group 1) and (d) depSympt post fixed effects adjustment (for use in interaction analysis with waist to hip ratio), in the available UK Biobank study population.

3.1.2 Covariates in Analysis group 2

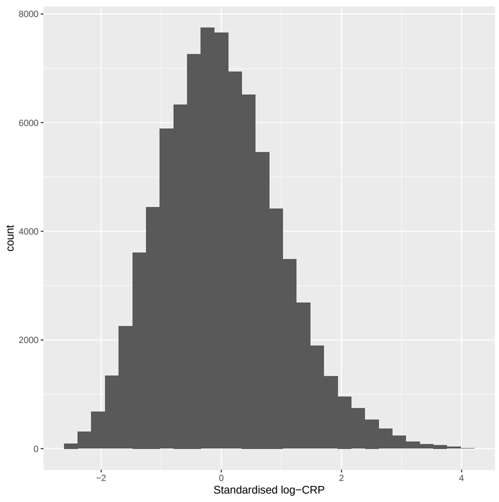

(a)

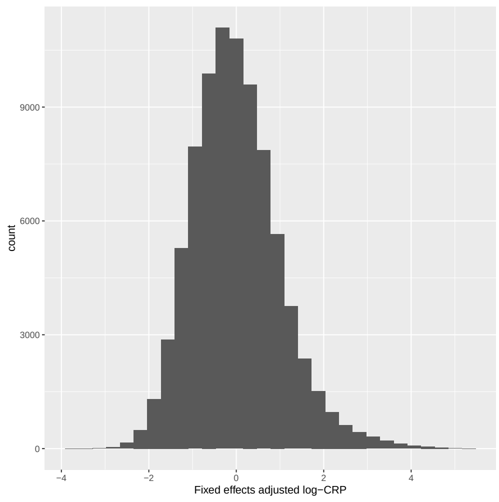

(b)

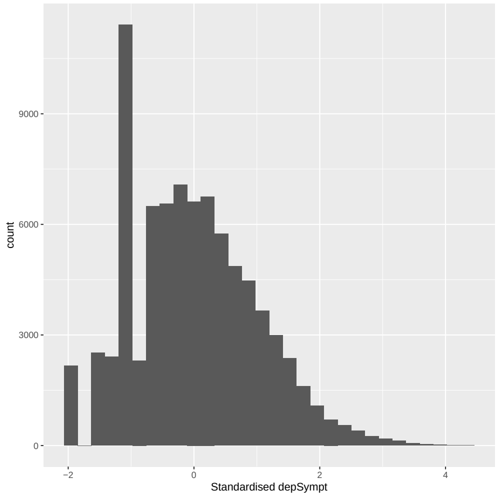

(c)

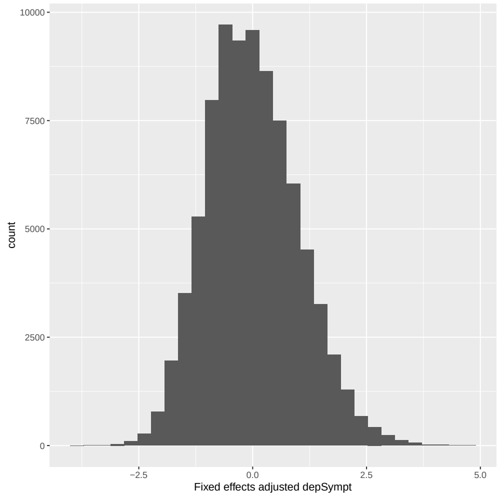

(d)

Figure 13: Histogram of standardised: (a) log-CRP, (b) log-CRP post fixed effects adjustment, (c) depSympt (Analysis group 2) and (d) depSympt post fixed effects adjustment (for use in interaction analysis with all group 2 covariates), in the available UK Biobank study population.

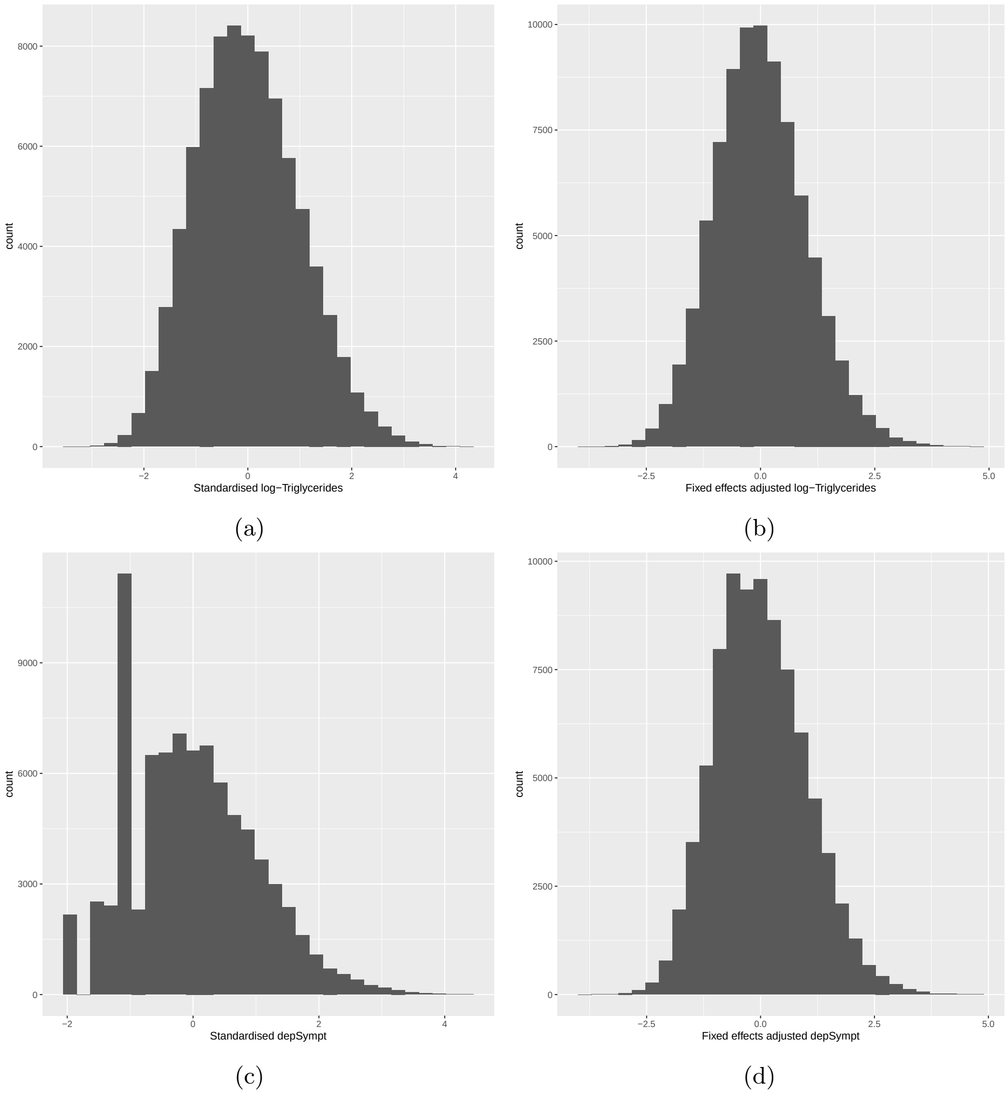

Figure 14: Histogram of standardised: (a) log-Triglycerides, (b) log-Triglycerides post fixed effects adjustment, (c) depSympt (Analysis group 2) and (d) depSympt post fixed effects adjustment (for use in interaction analysis with all group 2 covariates), in the available UK Biobank study population.

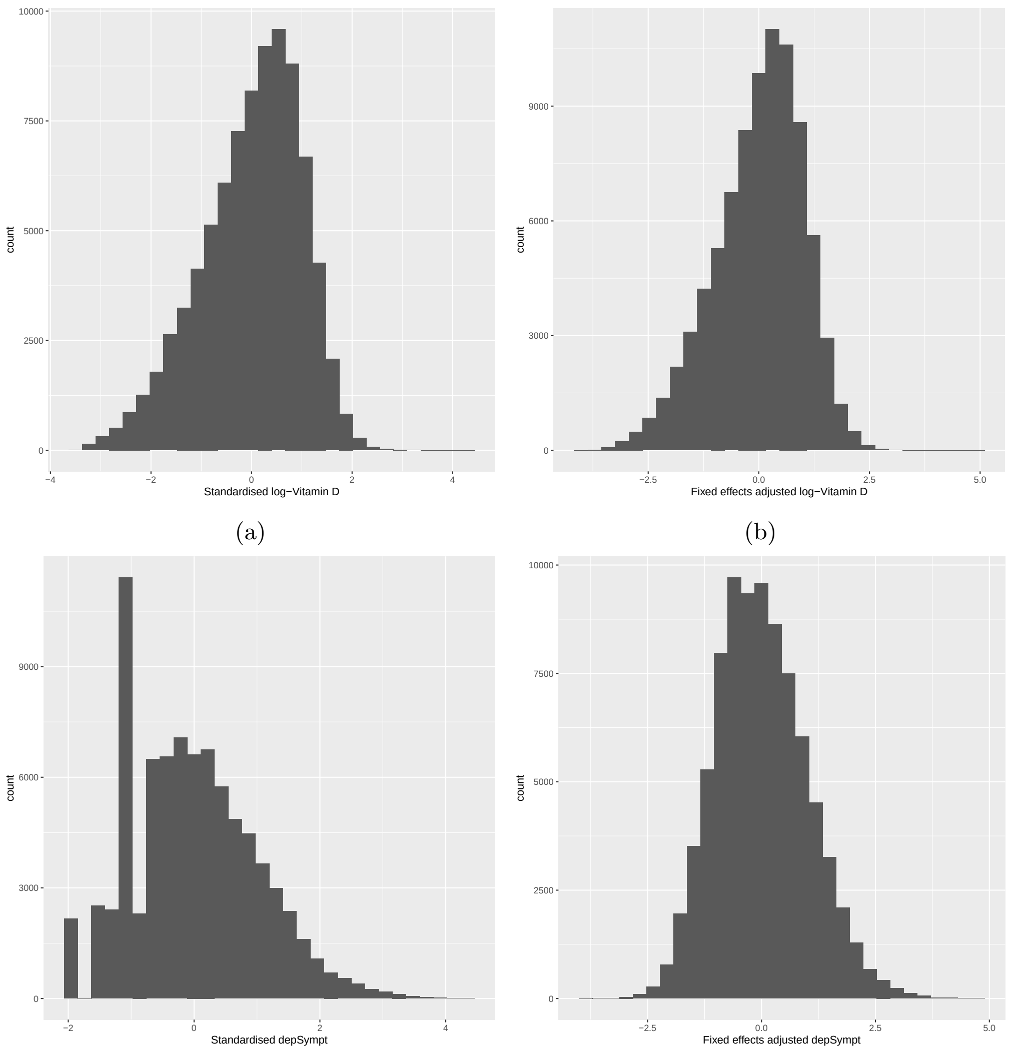

(a) (b) (c) (d)

Figure 15: Histogram of standardised: (a) log-Vitamin D, (b) log-Vitamin D post fixed effects adjustment, (c) depSympt (Analysis group 2) and (d) depSympt post fixed effects adjustment (for use in interaction analysis with all group 2 covariates), in the available UK Biobank study population.

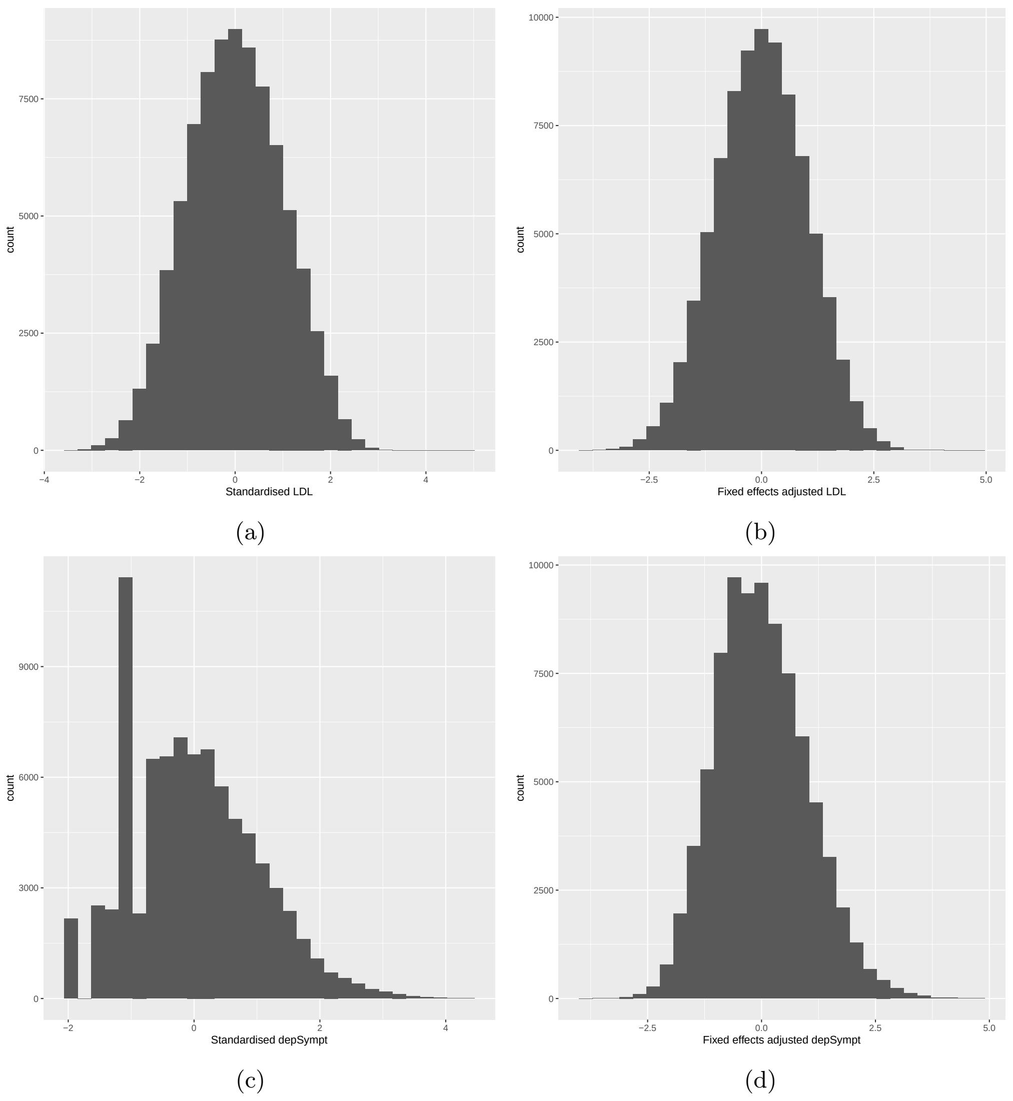

Figure 16: Histogram of standardised: (a) LDL, (b) LDL post fixed effects adjustment, (c) depSympt (Analysis group 2) and (d) depSympt post fixed effects adjustment (for use in interaction analysis with all group 2 covariates), in the available UK Biobank study population.

3.1.3 Covariates in Analysis group 3

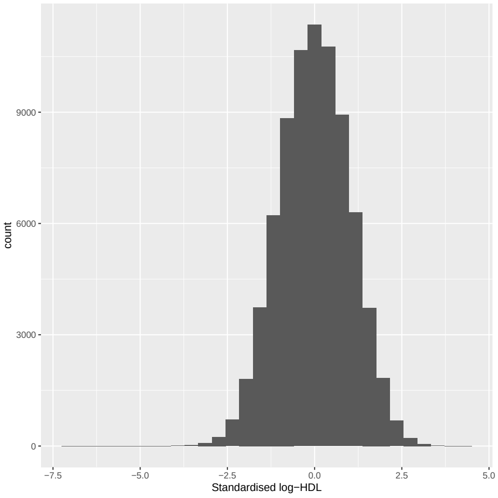

(a)

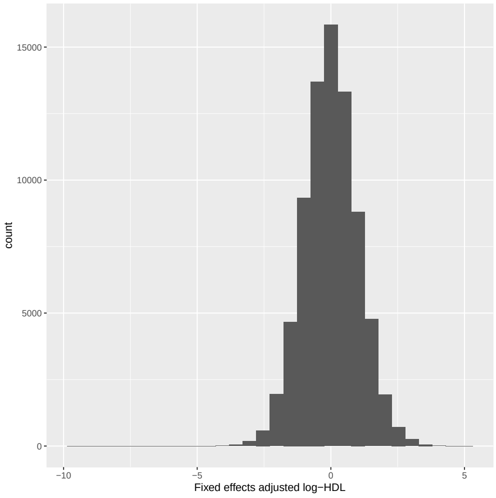

(b)

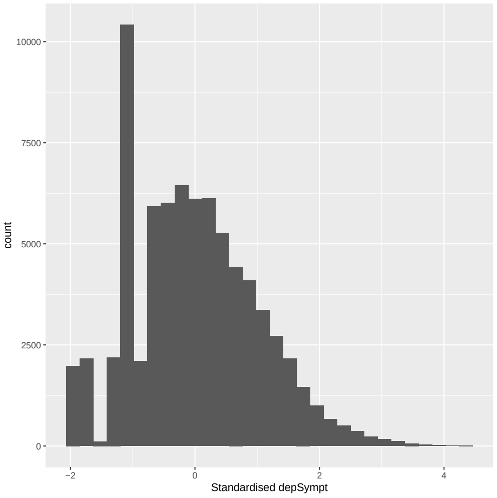

(c)

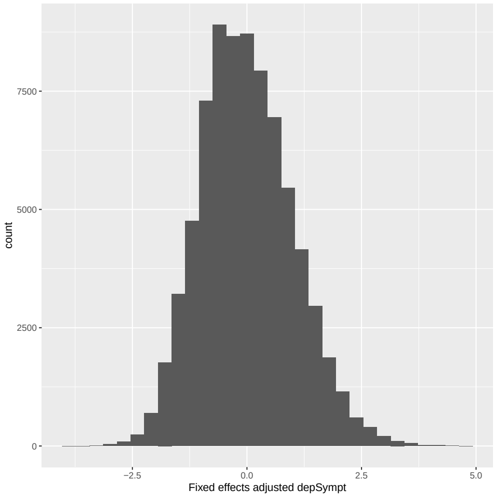

(d)

Figure 17: Histogram of standardised: (a) log-HDL, (b) log-HDL post fixed effects adjustment, (c) depSympt (Analysis group 3) and (d) depSympt post fixed effects adjustment (for use in interaction analysis with log-HDL), in the available UK Biobank study population.

3.1.4 Covariates in Analysis group 4

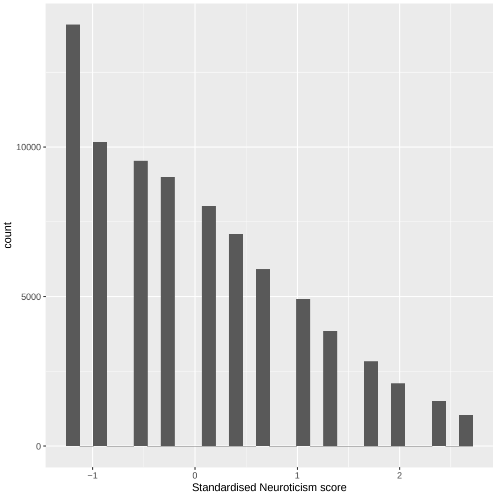

(a)

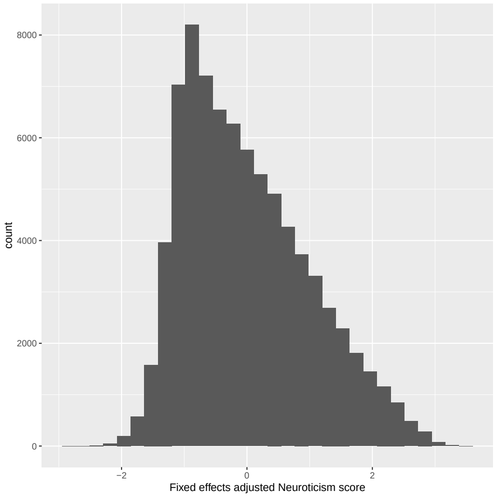

(b)

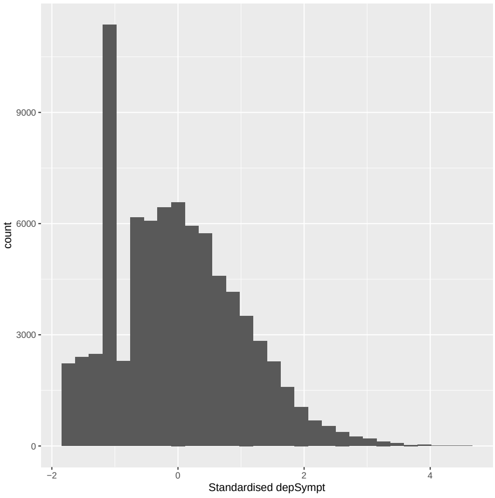

(c)

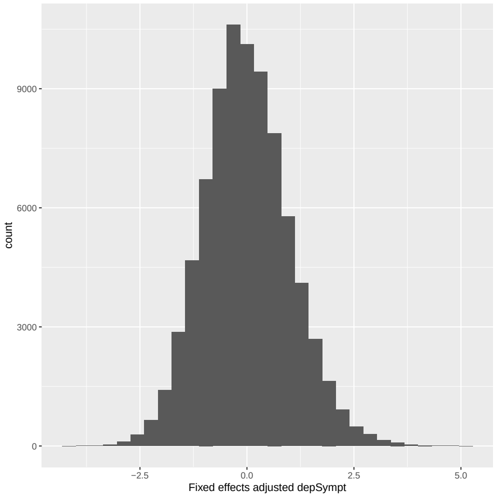

(d)

Figure 18: Histogram of standardised: (a) Neuroticism score, (b) Neuroticism score post fixed effects adjustment, (c) depSympt (Analysis group 4) and (d) depSympt post fixed effects adjustment (for use in interaction analysis with Neuroticism score), in the available UK Biobank study population.

3.1.5 Covariates in Analysis group 5

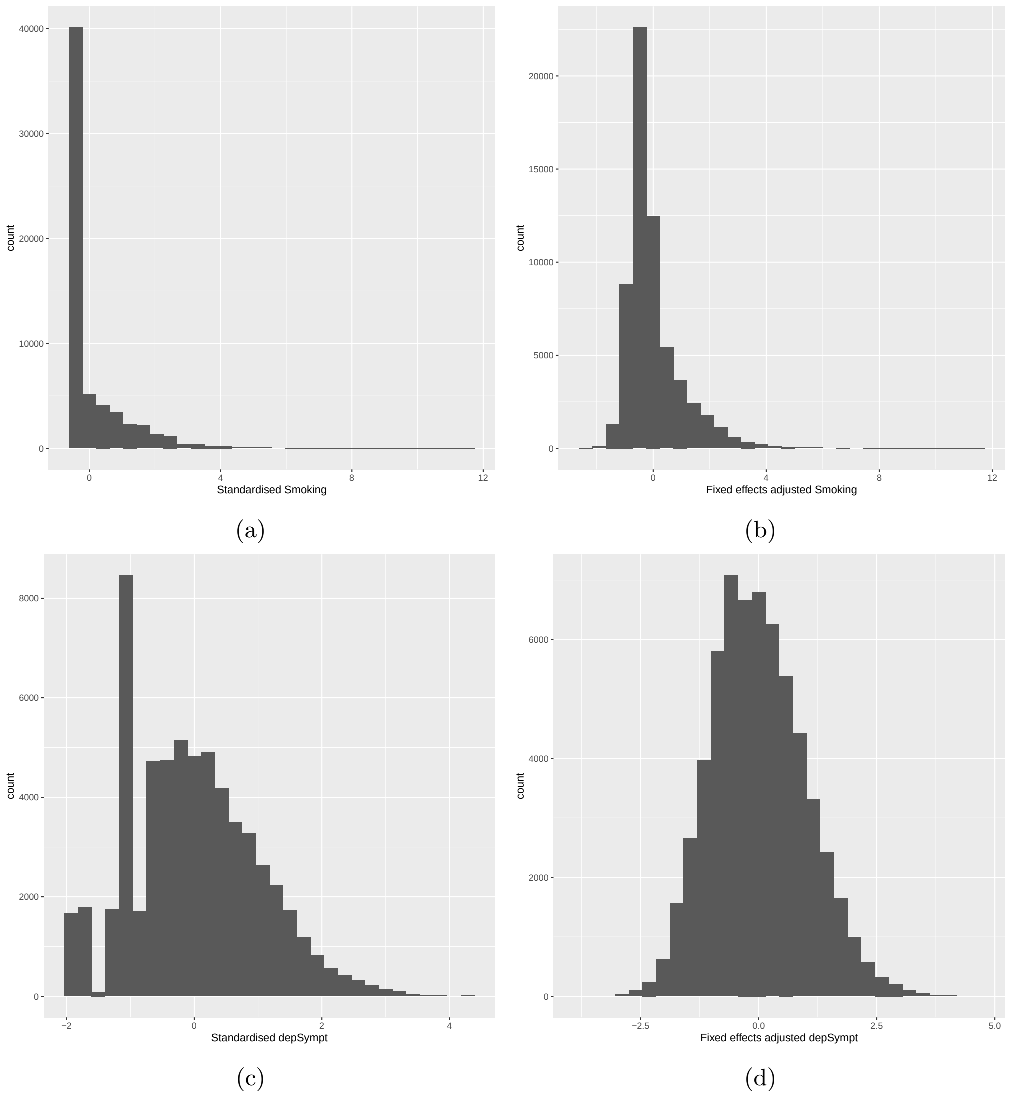

Figure 19: Histogram of standardised: (a) Smoking, (b) Smoking post fixed effects adjustment, (c) depSympt (Analysis group 5) and (d) depSympt post fixed effects adjustment (for use in interaction analysis with Smoking), in the available UK Biobank study population.

3.1.6 Biomarker distribution plots: untransformed compared to log transformed

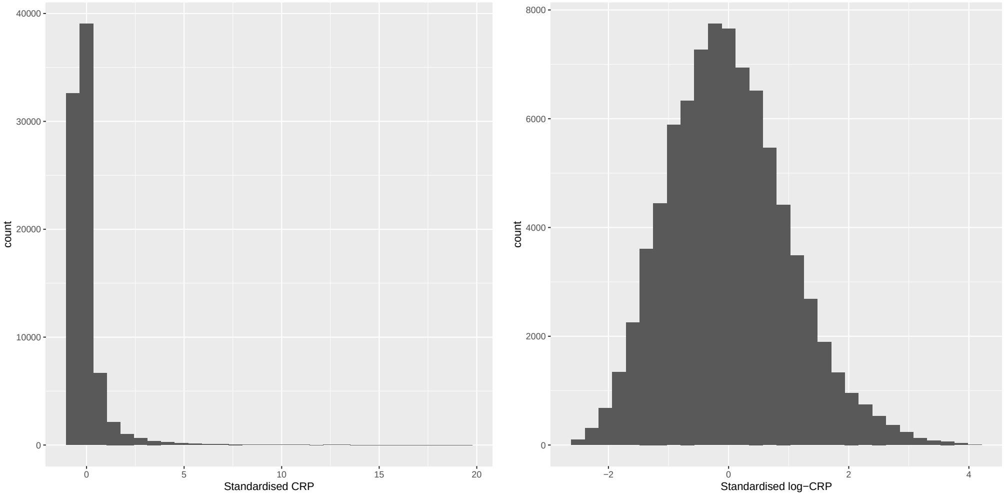

(a) (b)

Figure 20: Histogram of standardised: (a) CRP and (b) log-CRP ($N = 83,489$).

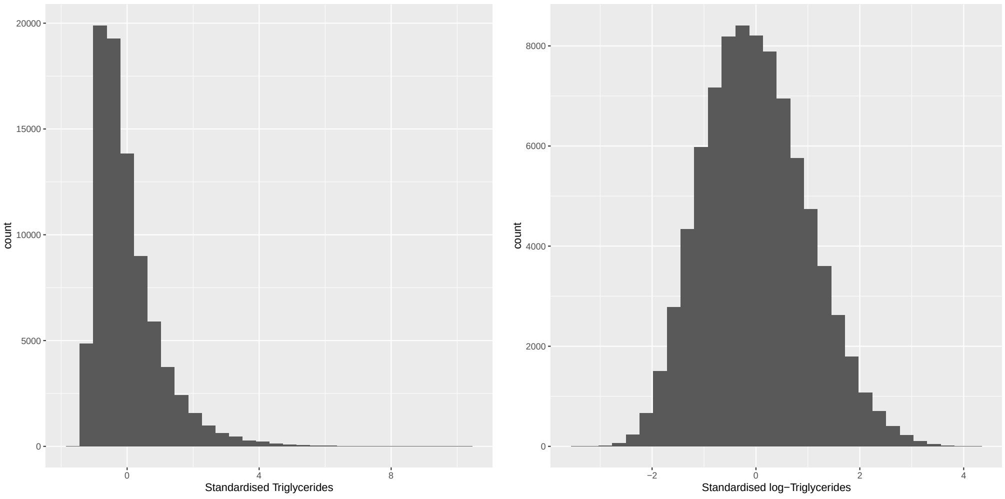

(a) (b)

Figure 21: Histogram of standardised: (a) Triglycerides and (b) log-Triglycerides ($N = 83,489$).

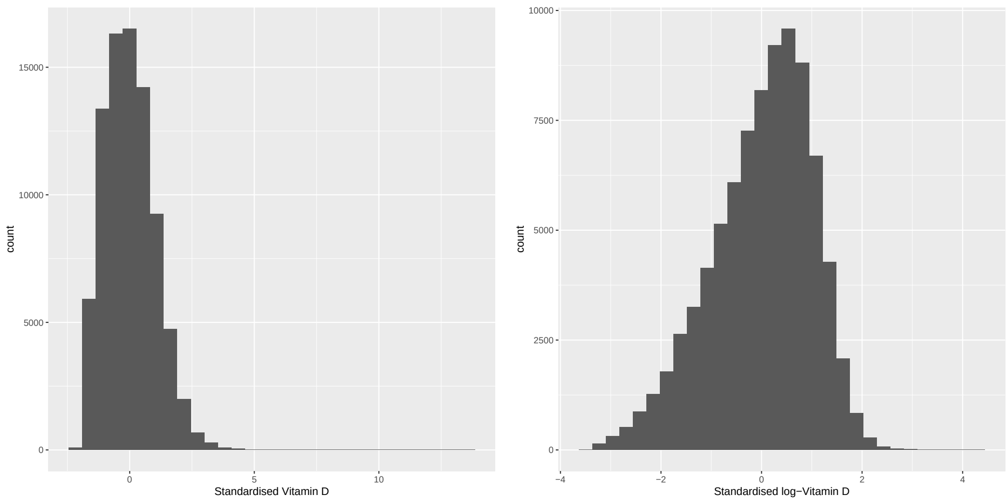

(a) (b)

Figure 22: Histogram of standardised: (a) Vitamin D and (b) log-Vitamin D ($N = 83,489$).

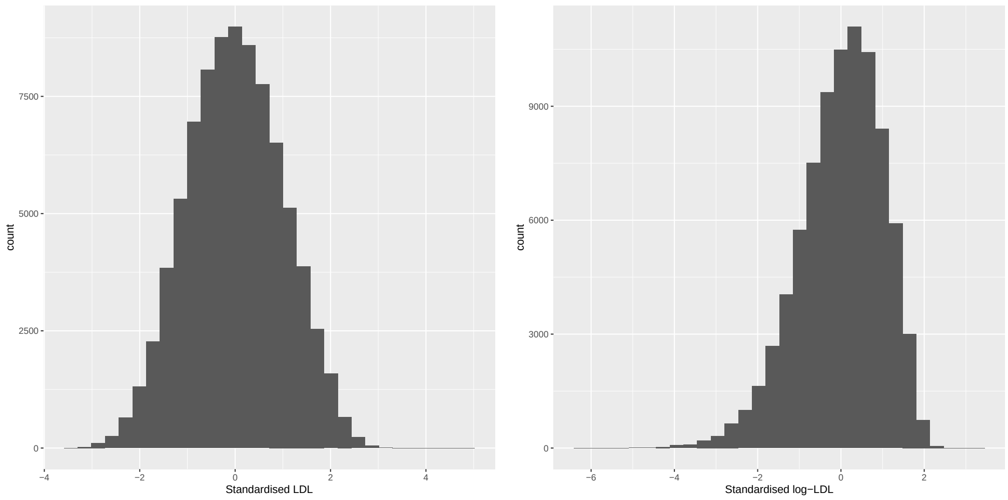

(a) (b)

Figure 23: Histogram of standardised: (a) LDL and (b) log-LDL ($N = 83,489$).

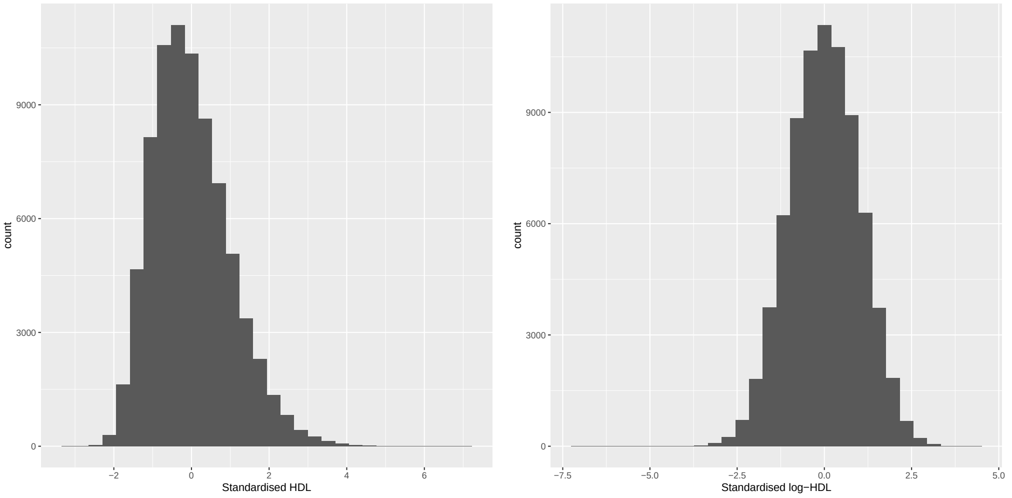

(a) (b)

Figure 24: Histogram of standardised: (a) HDL and (b) log-HDL ($N = 76,246$).

3.2 Fractional polynomial model results

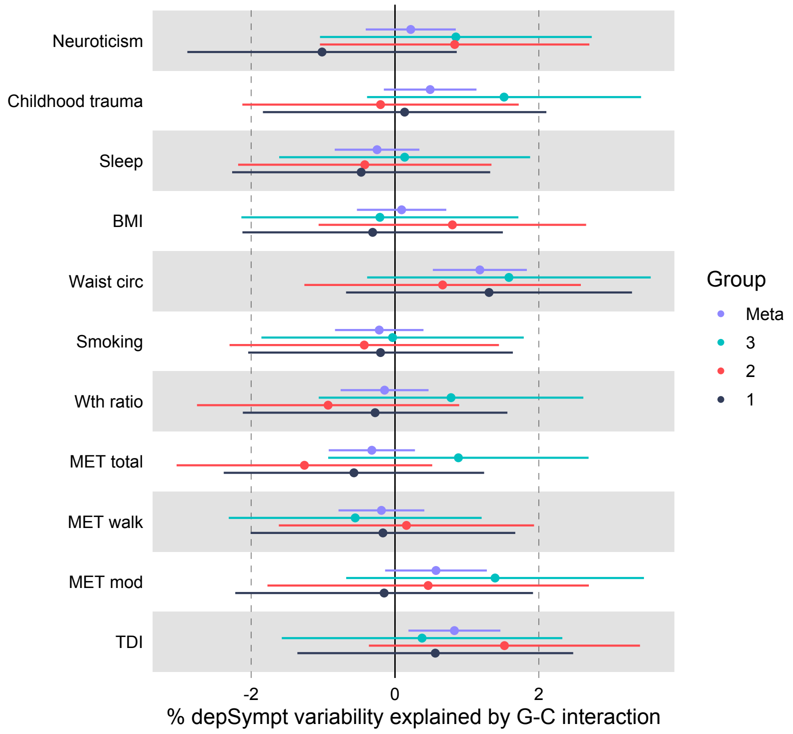

(a) Genotype-Covariate interaction

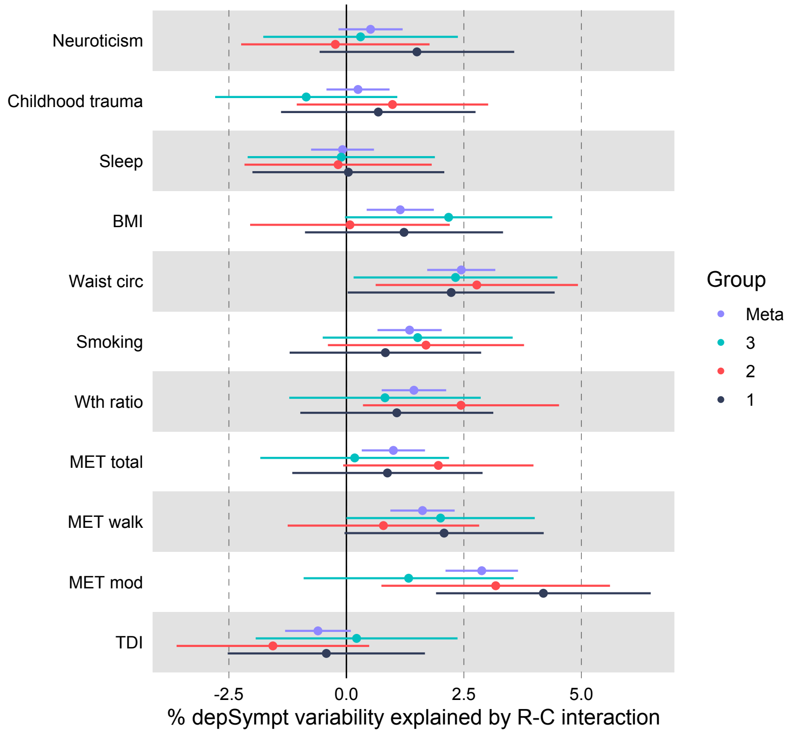

(b) Residual-Covariate interaction

Figure 25: The proportion of variation in depSympt attributable to: (a) a genotype-covariate interaction, and (b) a residual-covariate interaction, with 95% confidence intervals, for all 3 subgroups & the meta-analysis. The fixed effects component of the model is the FP model. Significant variables only.

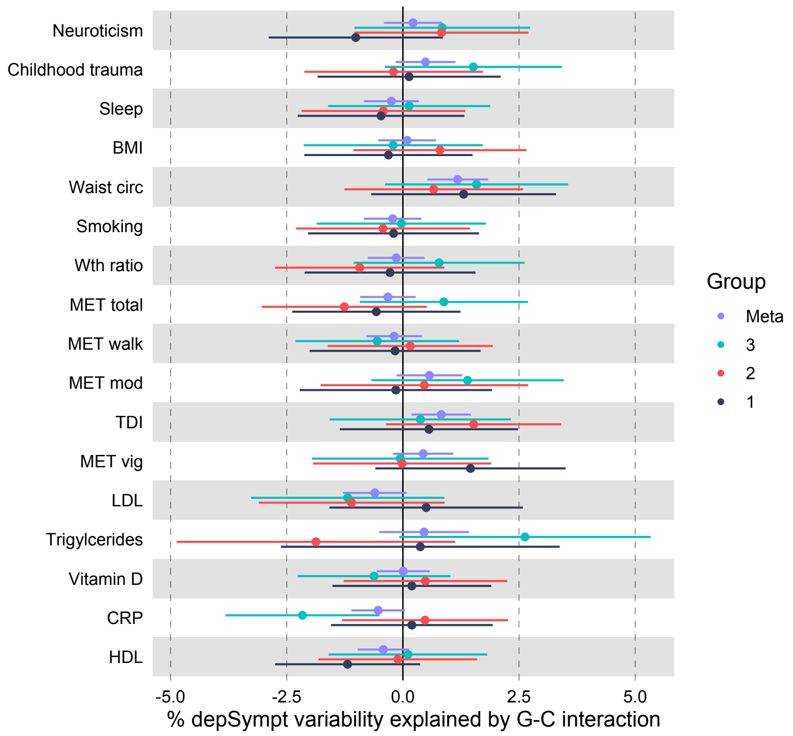

(a) Genotype-Covariate interaction

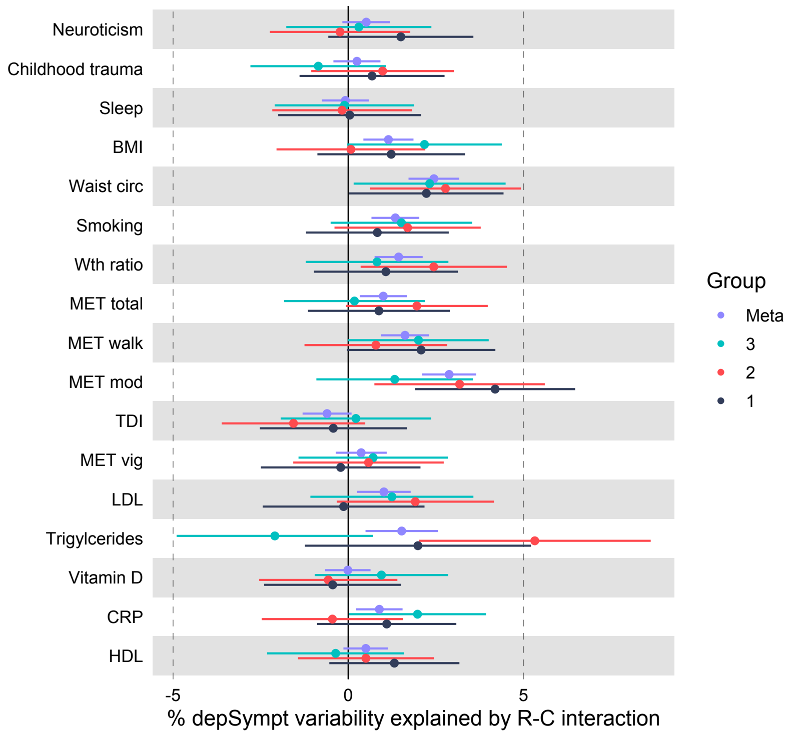

(b) Residual-Covariate interaction

Figure 26: The proportion of variation in depSympt attributable to: (a) a genotype-covariate interaction, and (b) a residual-covariate interaction, with 95% confidence intervals, for all 3 subgroups & the meta-analysis. The fixed effects component of the model is the FP model. All covariate traits.

Plots: Genetic, residual and total variance components of Y by covariate trait value

Significant covariates: Neuroticism score, childhood trauma summary variable, average sleep duration, BMI, waist circumference, smoking, waist to hip ratio, total MET minutes per week (MET (tot)), walking MET minutes per week (MET (walk)), moderate MET minutes per week (MET (mod)) and TDI.

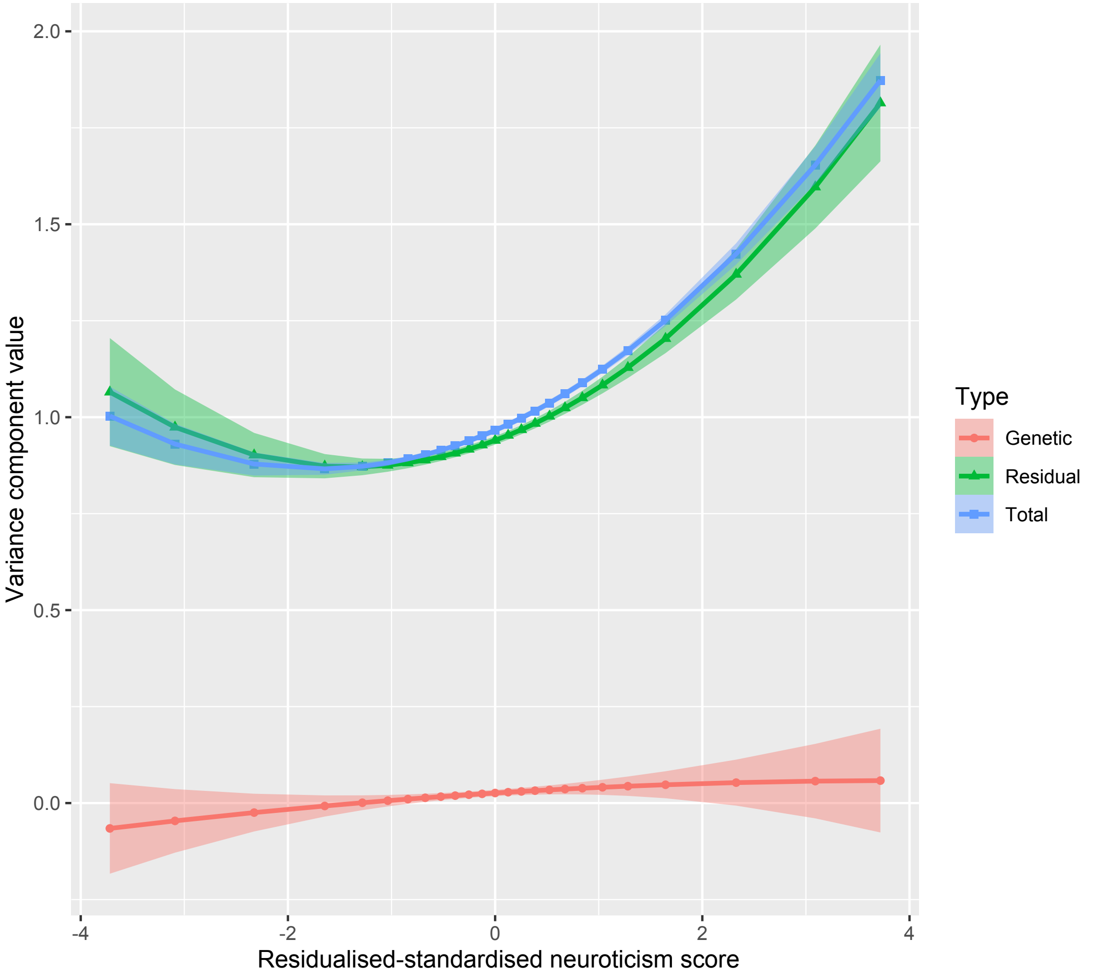

Figure 27: Variance components for Y (residualised depSympt) against residualised and standardised neuroticism score, with 95% confidence intervals.

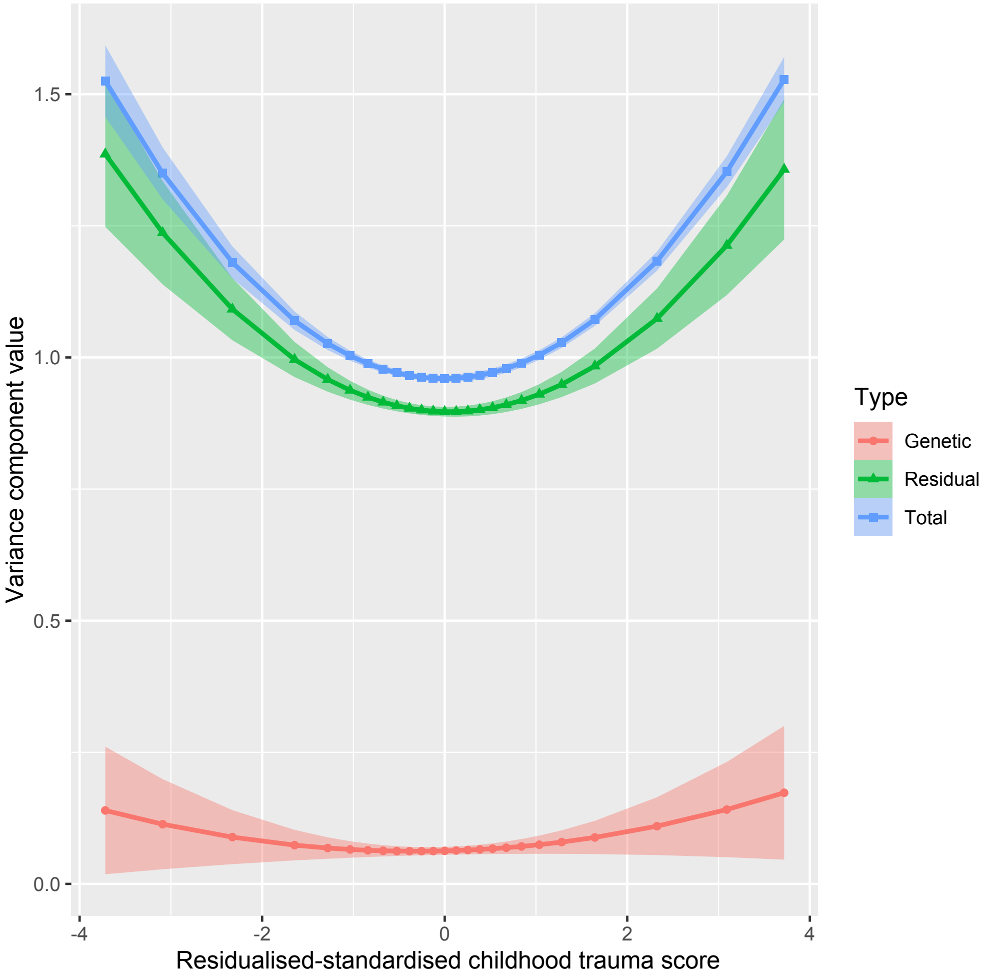

Figure 28: Variance components for Y (residualised depSympt) against residualised and standardised childhood trauma summary variable, with 95% confidence intervals.

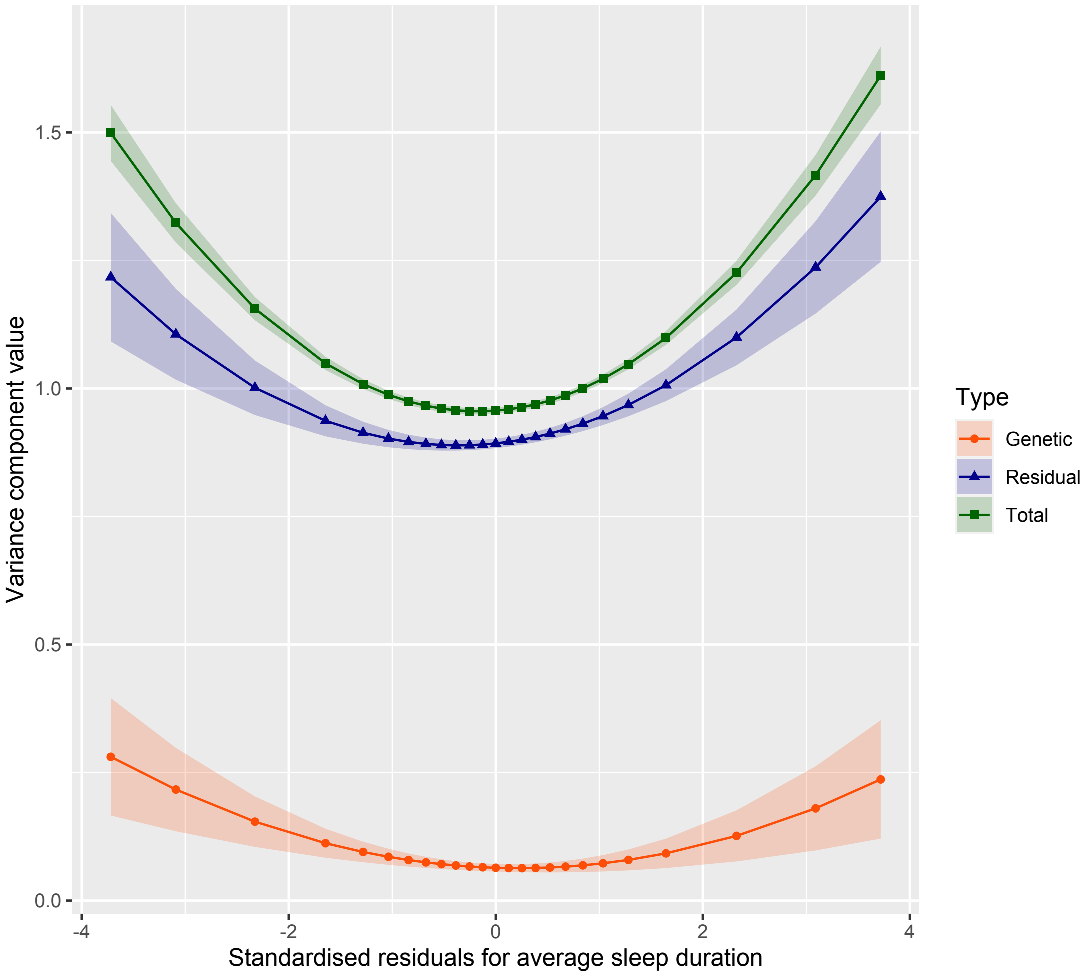

Figure 29: Variance components for Y (residualised depSympt) against residualised and standardised average sleep duration, with 95% confidence intervals.

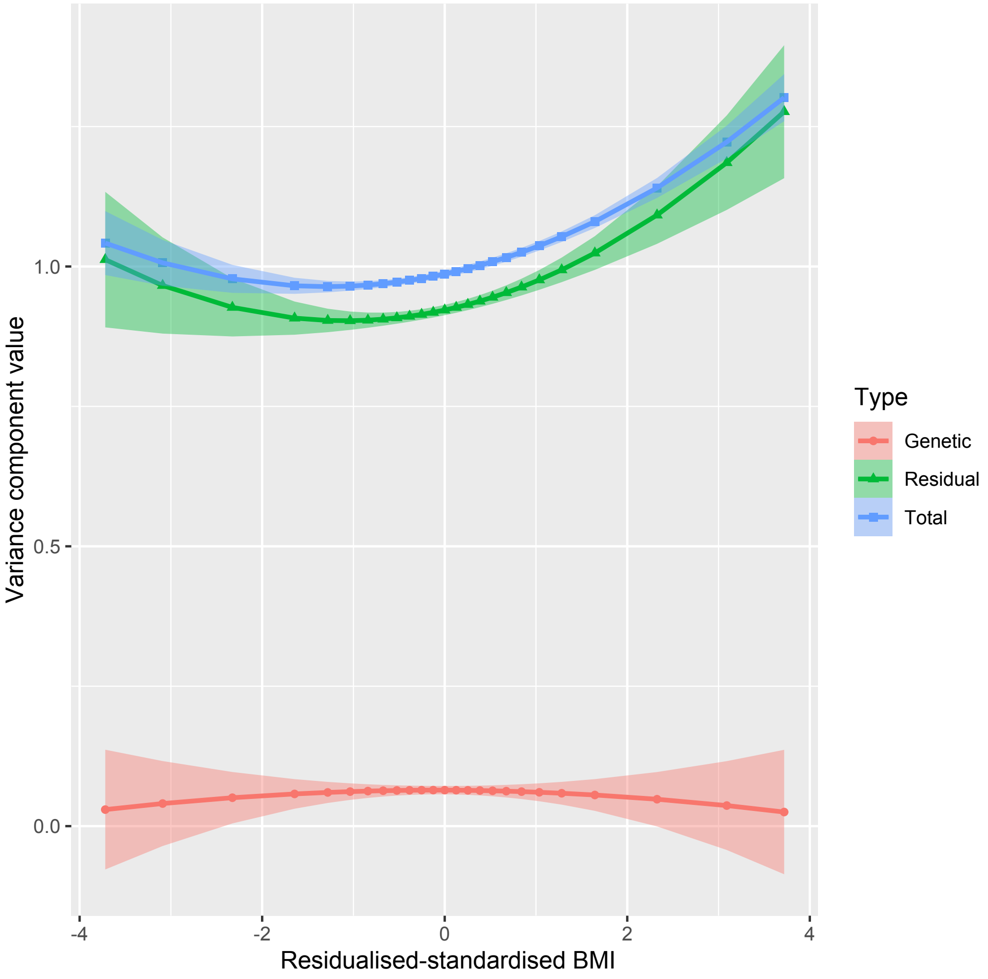

Figure 30: Variance components for Y (residualised depSympt) against residualised and standardised BMI, with 95% confidence intervals.

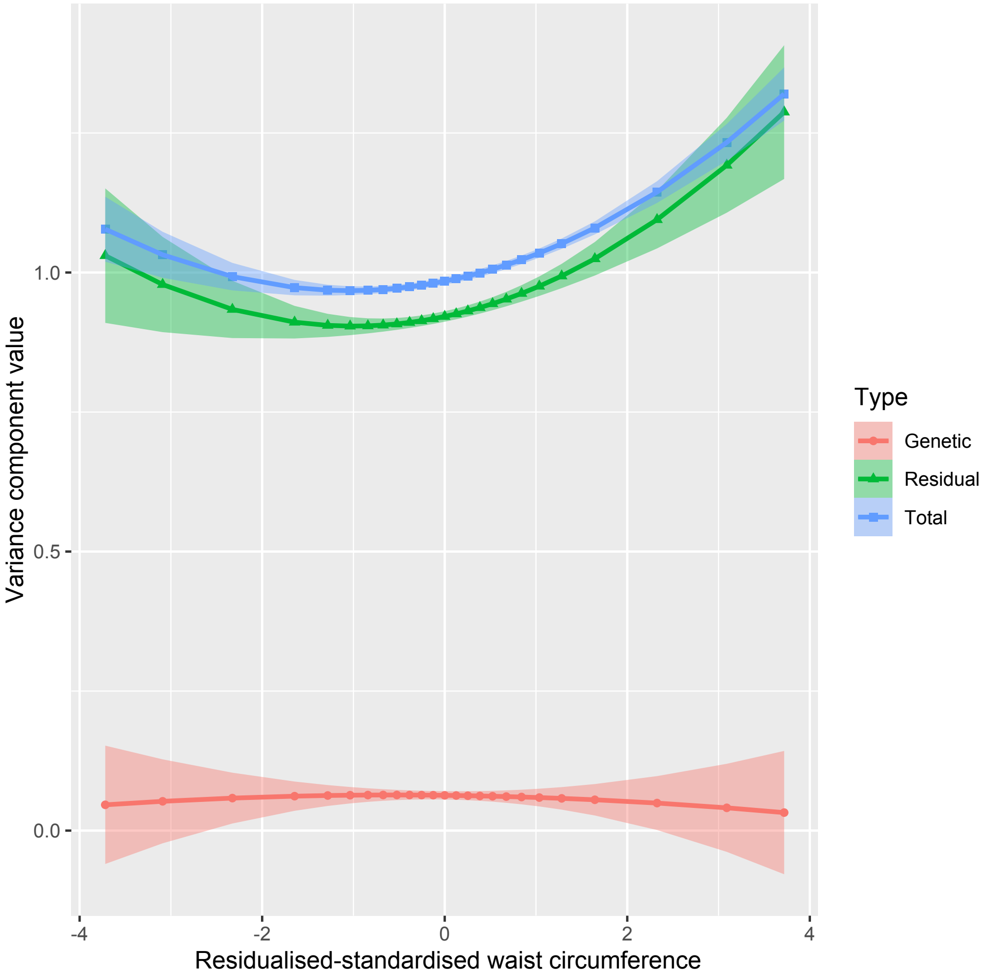

Figure 31: Variance components for Y (residualised depSympt) against residualised and standardised waist circumference, with 95% confidence intervals.

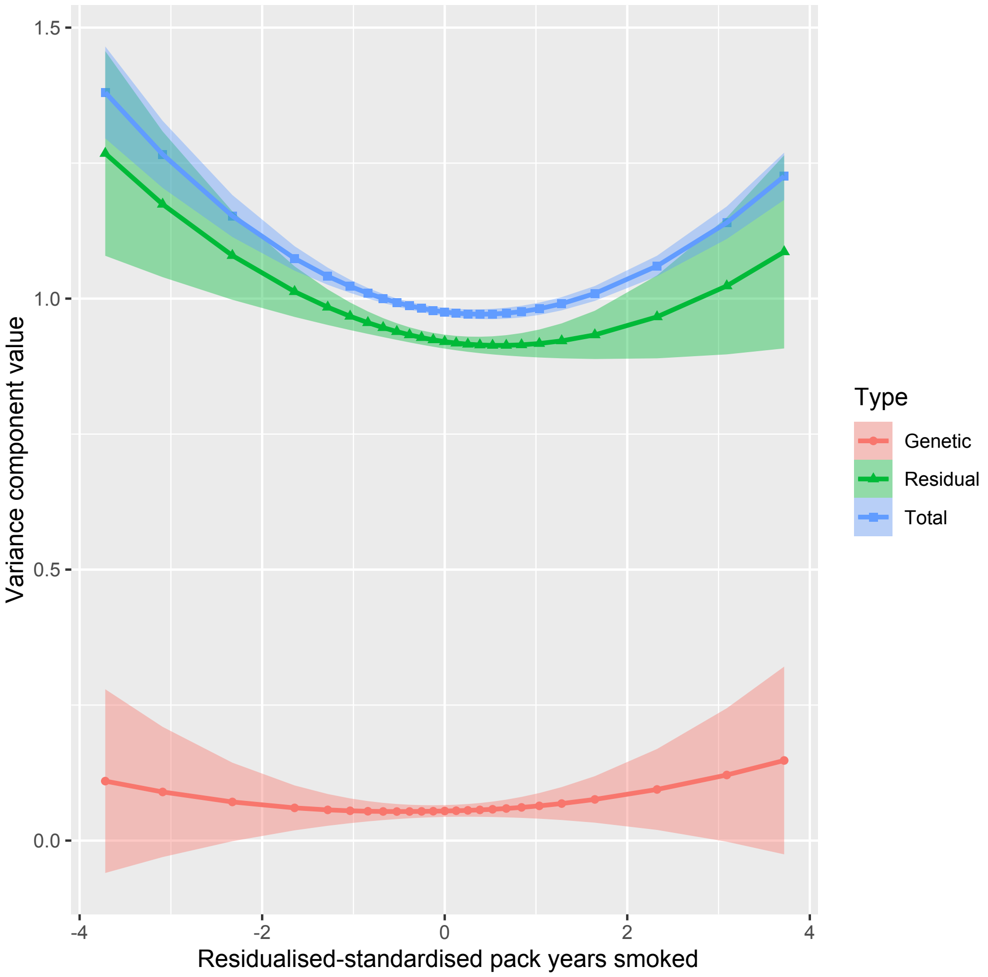

Figure 32: Variance components for Y (residualised depSympt) against residualised and standardised smoking, with 95% confidence intervals.

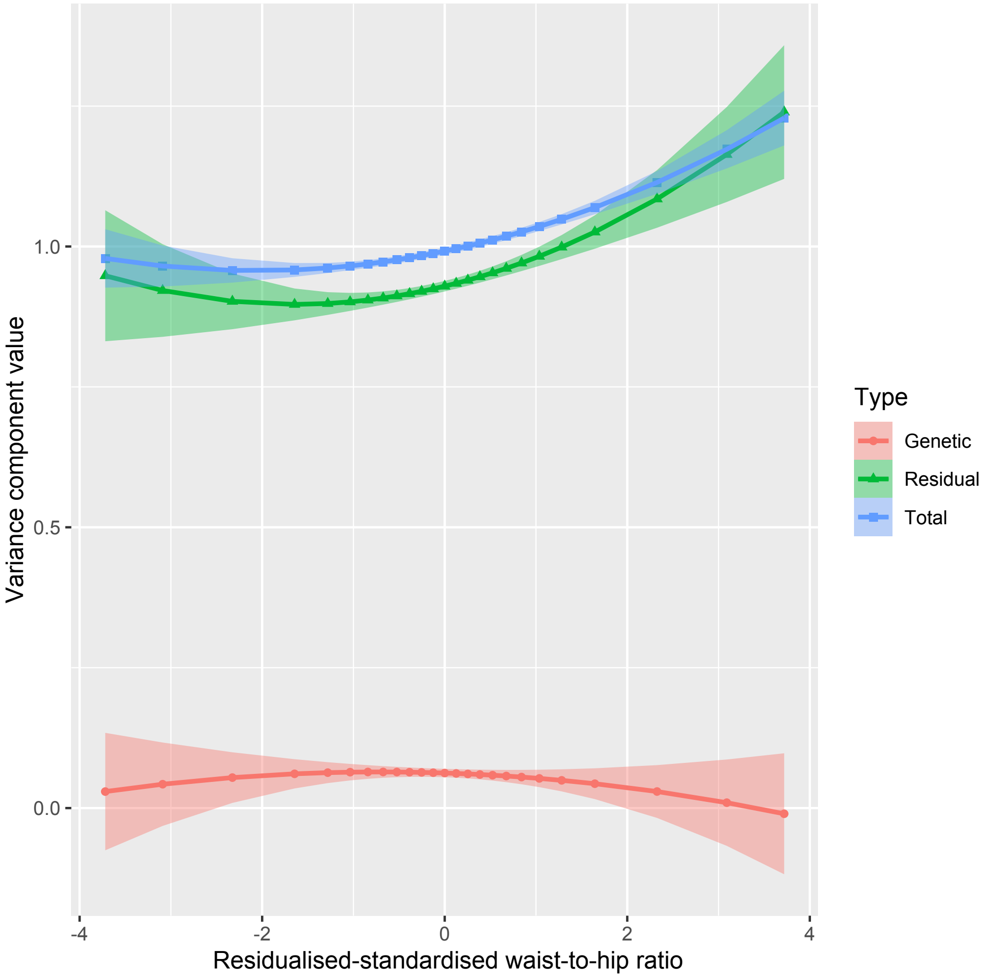

Figure 33: Variance components for Y (residualised depSympt) against residualised and standardised waist to hip ratio, with 95% confidence intervals.

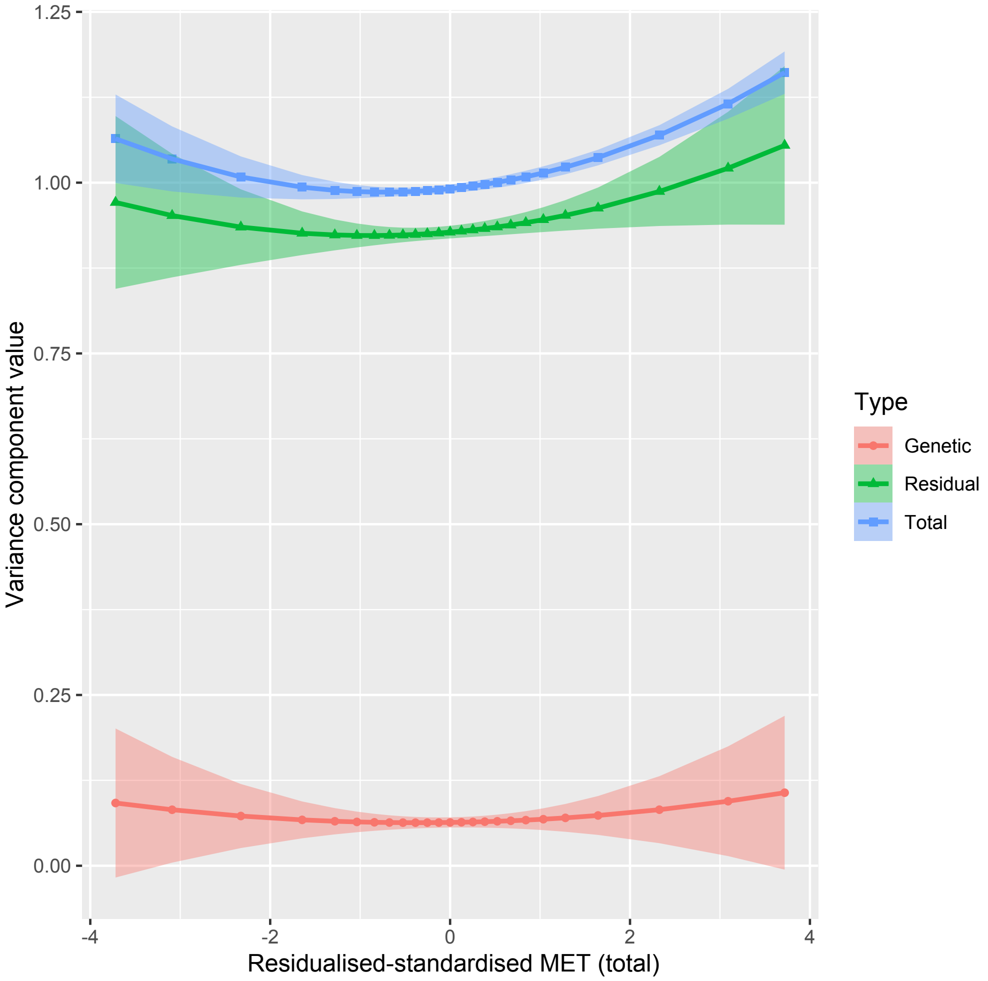

Figure 34: Variance components for Y (residualised depSympt) against residualised and standardised MET (tot), with 95% confidence intervals.

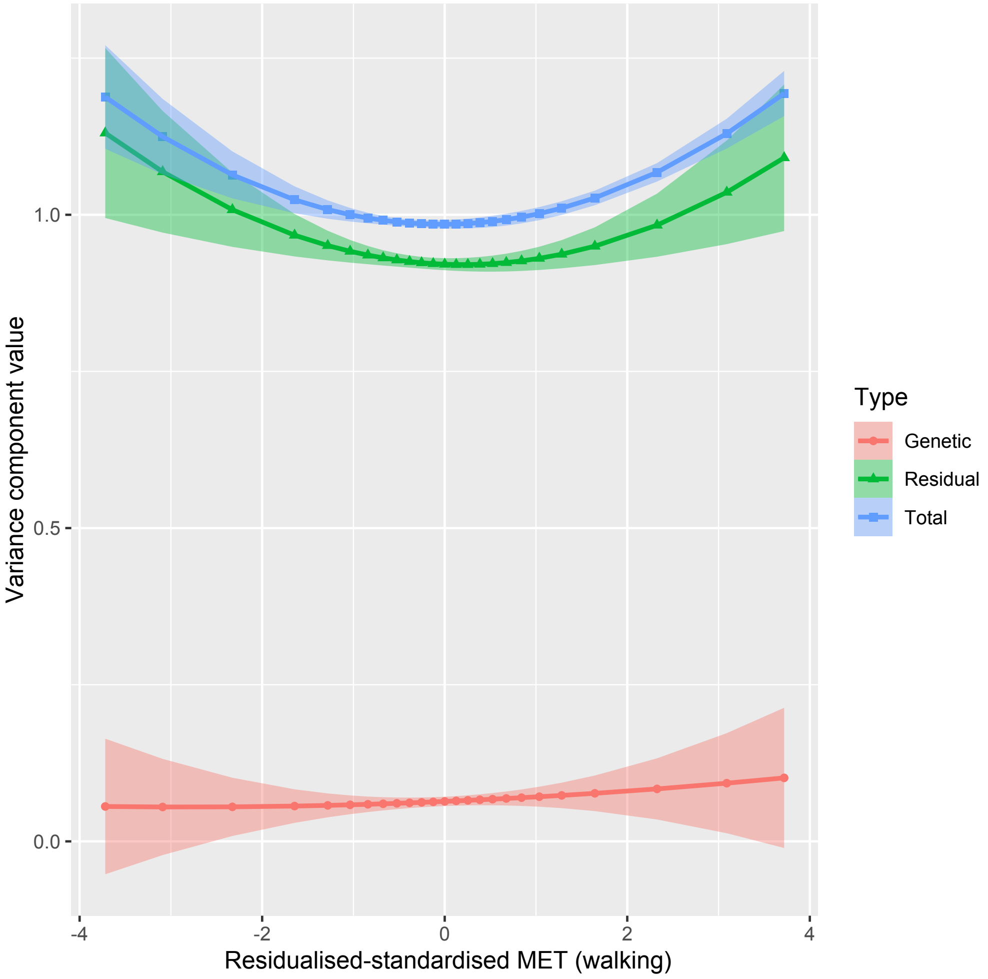

Figure 35: Variance components for Y (residualised depSympt) against residualised and standardised MET (walk), with 95% confidence intervals.

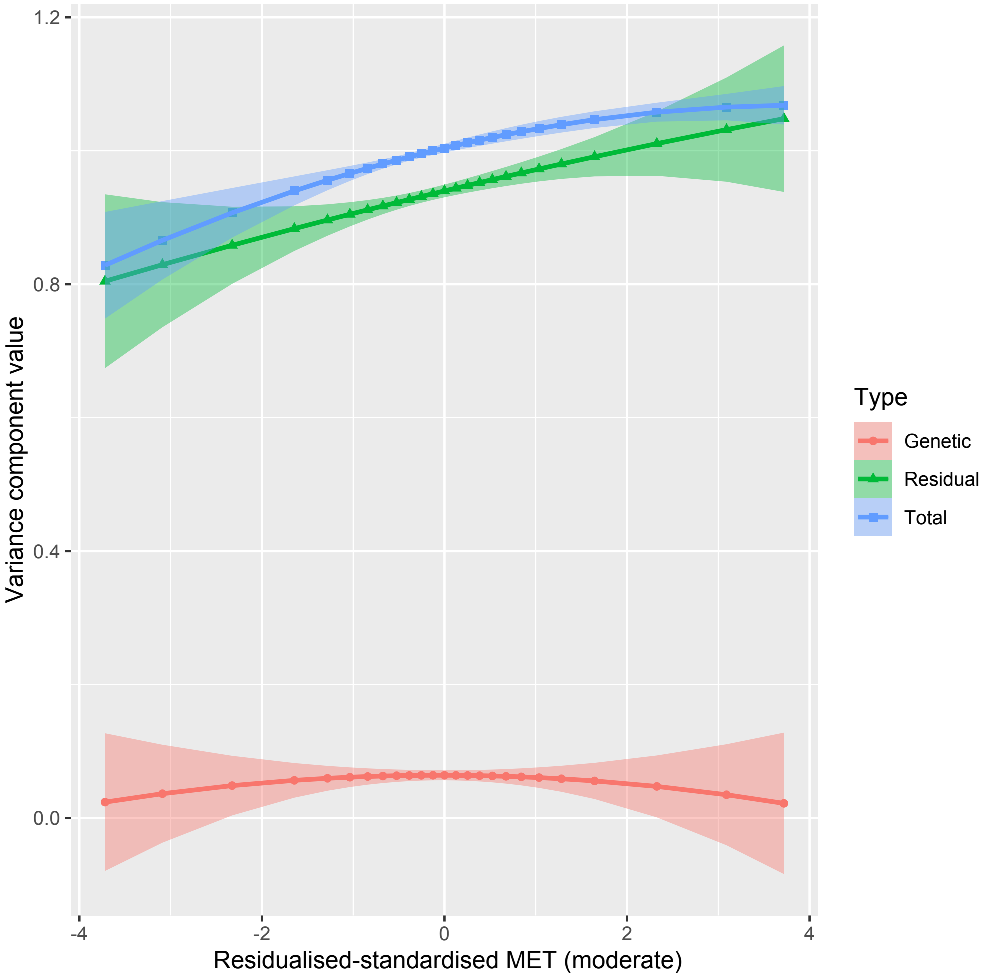

Figure 36: Variance components for Y (residualised depSympt) against residualised and standardised MET (mod), with 95% confidence intervals.

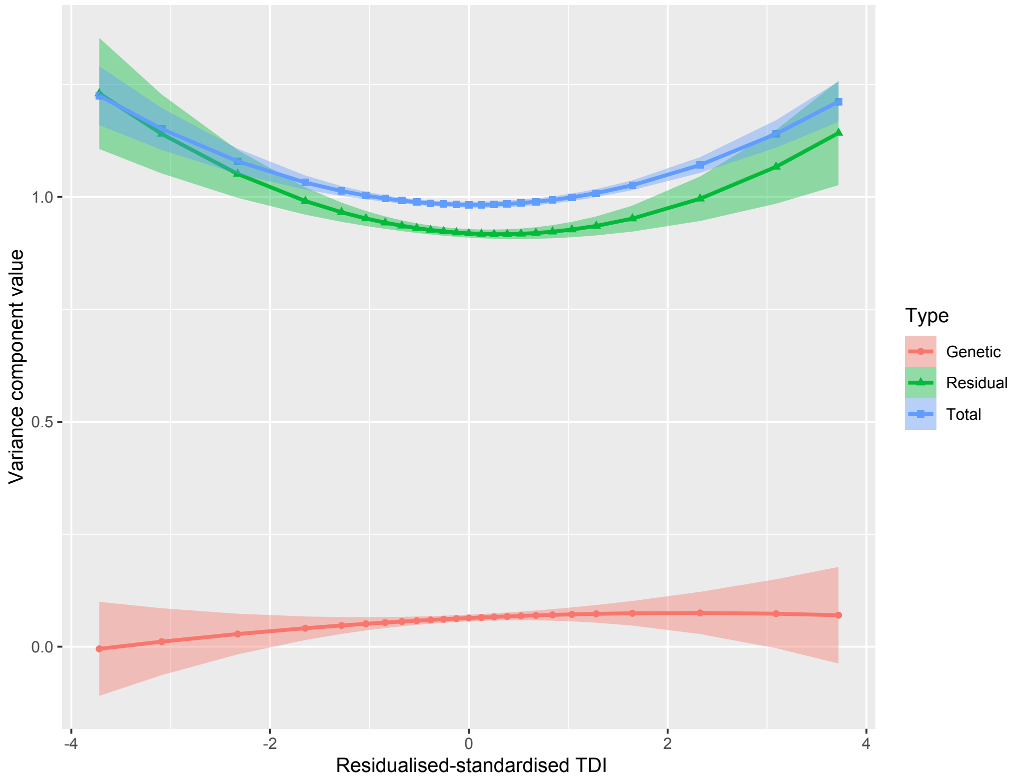

Figure 37: Variance components for Y (residualised depSympt) against residualised and standardised TDI, with 95% confidence intervals.

Heritability plot for sleep

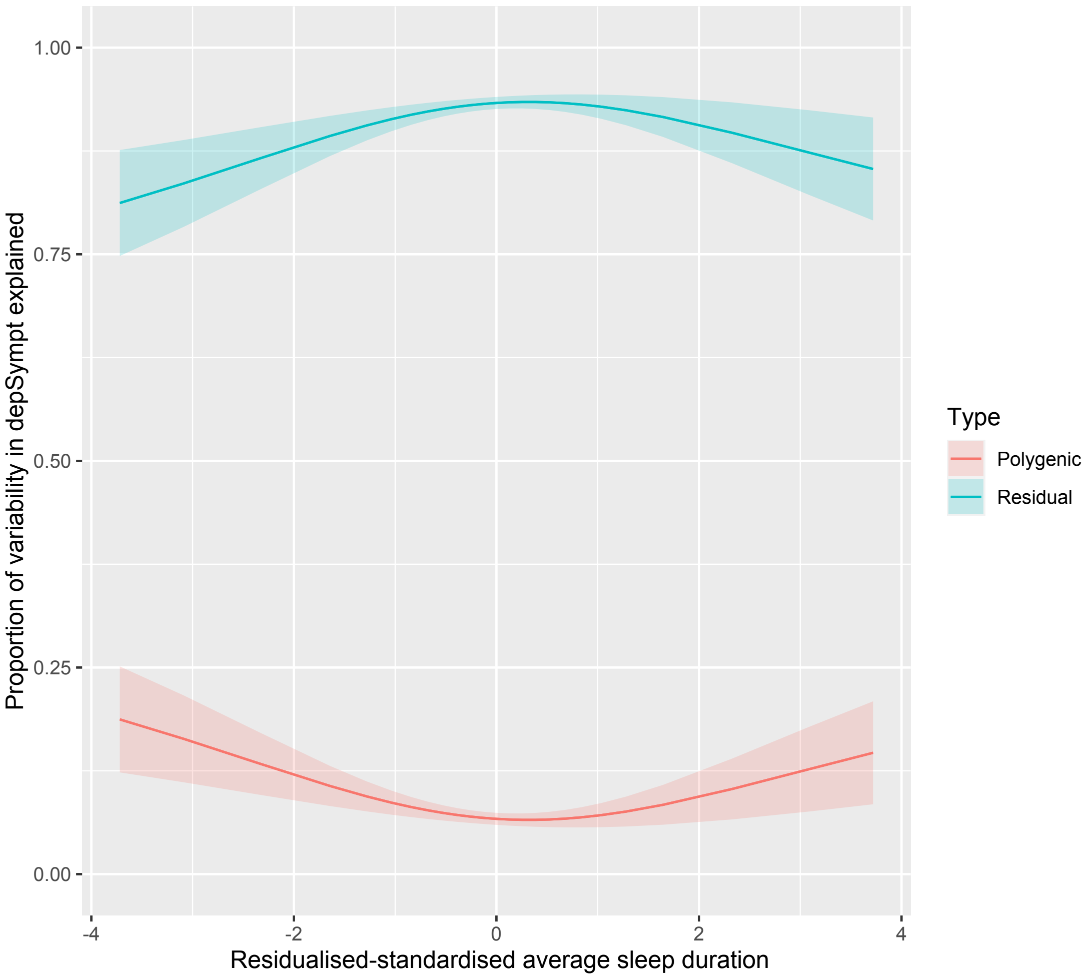

Figure 38: Proportion of the total variance component for Y (residualised depSympt) attributable to the genetic component (heritability) and the residual component, as a function of residualised and standardised average sleep duration, with 95% confidence intervals.

Plots for MET (tot) and MET (mod) exploration

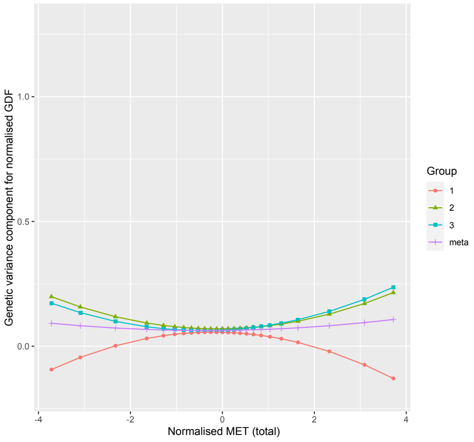

(a) Genetic variance component

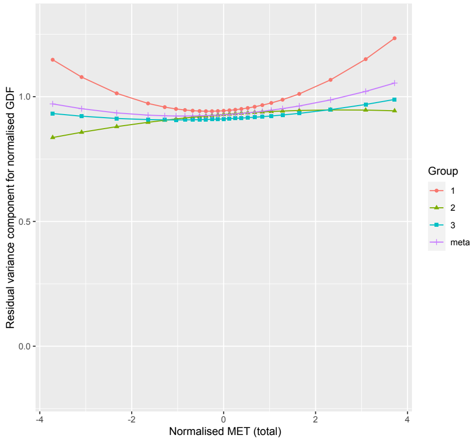

(b) Residual variance component

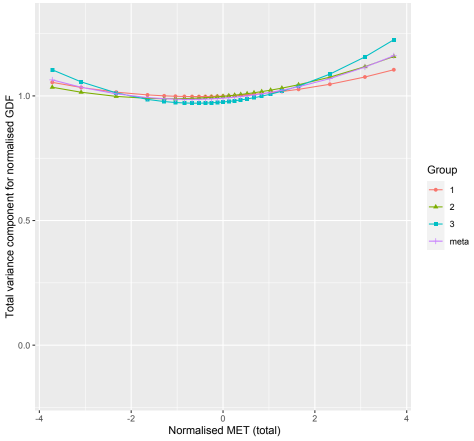

(c) Total variance component

Figure 39: Plots of the variance components for normalised depSympt across normalised MET (total)

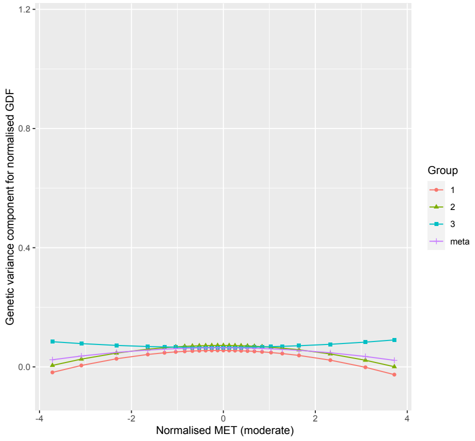

(a) Genetic variance component

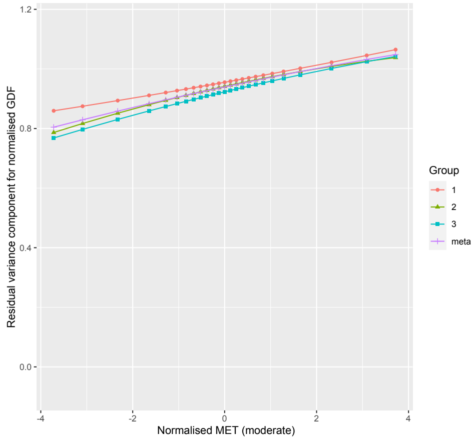

(b) Residual variance component

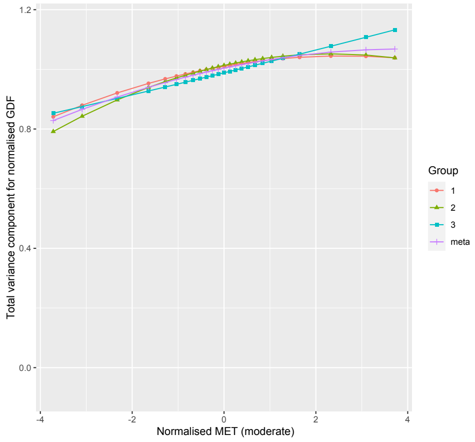

(c) Total variance component

Figure 40: Plots of the variance components for normalised depSympt across normalised MET (moderate)

References

A. Benner and G. Ambler. *mfp: Multivariable Fractional Polynomials.* https://CRAN.R-project.org/package=mfp, 2015.

E. Evangelou and J. P. A. Ioannidis. Meta-analysis methods for genome-wide association studies and beyond. *Nature Reviews Genetics*, 14:379–389, 2013.

J. B. Hall and W. S. Bush. Analysis of heritability using genome-wide data. *Current protocols in human genetics*, 91:1.30.1–1.30.10, 10 2016.

L. V. Hedges and J. L. Vevea. Fixed and random effects models in meta analysis. *Psychological Methods*, 3(4):486–504, 1998.

D. Jarquin, J. Crossa, X. Lacaze, P. Du Cheyron, J. Daucourt, J. Lorgeou, F. Piraux, L. Guerreiro, P. Perez, M. Calus, J. Burgueno, and G. de los Campos. A reaction norm model for genomic selection using high-dimensional genomic and environmental data. *Theoretical and applied genetics*, 127:595–607, 2014.

B. S. Jermy, S. P. Hagenaars, K. P. Glanville, J. R. I. Coleman, D. M. Howard, G. Breen, E. Vassos, and C. M. Lewis. Using major depression polygenic risk scores to explore the depressive symptom continuum. *Psychological Medicine*, pages 1–10, 2020. doi: DOI: 10.1017/S0033291720001828.

S. H. Lee and J. H. J. van der Werf. Mtg2: an efficient algorithm for multivariate linear mixed model analysis based on genomic information. *Bioinformatics (Oxford, England)*, 32(9):1420–1422, 05 2016. doi: 10.1093/bioinformatics/btw012. URL `https://www.ncbi.nlm.nih.gov/pubmed/26755623`.

G. Ni, J. van der Werf, X. Zhou, E. Hyppönen, N. R. Wray, and S. H. Lee. Genotype–covariate correlation and interaction disentangled by a whole-genome multivariate reaction norm model. *Nature Communications*, 10(1):2239, 2019. doi: 10.1038/s41467-019-10128-w. URL `https://doi.org/10.1038/s41467-019-10128-w`.

M. C. Pitharouli, S. P. Hagenaars, K. P. Glanville, J. R. I. Coleman, M. Hotopf, C. M. Lewis, and C. M. Pariante. Depressed patients have elevated c-reactive protein independently of genetic, health and psychosocial factors, in the uk biobank. *American Journal of Psychiatry*, 178(6):522–529, 2021.

R Core Team. *R: A Language and Environment for Statistical Computing.* R Foundation for Statistical Computing, Vienna, Austria, 2020.

P. Royston and D. G. Altman. Regression using fractional polynomials of continuous covariates: parsimonious parametric modelling (with discussion). *Applied Statistics*, 43(3):429–467, 1994.

L. R. Schaeffer. Application of random regression models in animal breeding. *Livestock Production Science*, 86(1):35–45, 2004.

W. Viechtbauer. Conducting meta-analyses in R with the metafor package. *Journal of Statistical Software*, 36(3):1–48, 2010.

Z. Xuan, van der Werf Julius, C. Kristin, N. Guiyan, M. John, H. Elina, and L. S. Hong. Wholegenome approach discovers novel genetic and nongenetic variance components modulated by lifestyle for cardiovascular health. *Journal of the American Heart Association*, 9(8):e015661, 2021/01/12 2020.
